# Widespread use of unconventional targeting signals in mitochondrial ribosome proteins

**DOI:** 10.15252/embj.2021109519

**Published:** 2021-11-17

**Authors:** Yury S Bykov, Tamara Flohr, Felix Boos, Naama Zung, Johannes M Herrmann, Maya Schuldiner

**Affiliations:** ^1^ Department of Molecular Genetics Weizmann Institute of Science Rehovot Israel; ^2^ Division of Cell Biology University of Kaiserslautern Kaiserslautern Germany; ^3^ Present address: Department of Genetics Stanford University Stanford CA USA

**Keywords:** mitochondria, mitochondrial ribosome, mitochondrial targeting signal, targeting, translocation, Evolution & Ecology, Organelles, Translation & Protein Quality

## Abstract

Mitochondrial ribosomes are complex molecular machines indispensable for respiration. Their assembly involves the import of several dozens of mitochondrial ribosomal proteins (MRPs), encoded in the nuclear genome, into the mitochondrial matrix. Proteomic and structural data as well as computational predictions indicate that up to 25% of yeast MRPs do not have a conventional N‐terminal mitochondrial targeting signal (MTS). We experimentally characterized a set of 15 yeast MRPs *in vivo* and found that five use internal MTSs. Further analysis of a conserved model MRP, Mrp17/bS6m, revealed the identity of the internal targeting signal. Similar to conventional MTS‐containing proteins, the internal sequence mediates binding to TOM complexes. The entire sequence of Mrp17 contains positive charges mediating translocation. The fact that these sequence properties could not be reliably predicted by standard methods shows that mitochondrial protein targeting is more versatile than expected. We hypothesize that structural constraints imposed by ribosome assembly interfaces may have disfavored N‐terminal presequences and driven the evolution of internal targeting signals in MRPs.

## Graphical abstract

Possibly due to structural constraints, a subset of cytosolically synthesized mitochondrial matrix proteins is targeted via internal sequences, rather than prepeptides.

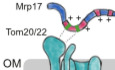

## Introduction

Mitochondria are descendants of ancient bacteria that formed eukaryotic cells together with their archaeal host (Sagan, 1967; Zaremba‐Niedzwiedzka *et al*, 2017; Martijn *et al*, 2018). Since then, mitochondria have lost their autonomy and their reproduction depends entirely on the nuclear genome, which encodes the majority of mitochondrial proteins. However, all mitochondria capable of respiration have retained small vestigial genomes of their own and fully functional gene expression machineries of bacterial origin (Roger *et al*, 2017). Mitochondrial ribosomes (mitoribosomes) are the most complex components of the mitochondrial gene expression system and consist of several RNA molecules and 60 to 80 different proteins (Greber & Ban, 2016; Ott *et al*, 2016). Mitoribosome dysfunction has adverse consequences leading to a broad spectrum of diseases (Boczonadi & Horvath, 2014).

While it took many years to solve the first ribosome structures (Ban *et al*, 2000; Carter *et al*, 2000; Schluenzen *et al*, 2000), the progress in cryo‐electron microscopy is now rapidly revealing the structural details of mitoribosomes of many different taxonomic groups (Amunts *et al*, 2015; Desai *et al*, 2017; Kummer *et al*, 2018; Ramrath *et al*, 2018; Itoh *et al*, 2020; Tobiasson & Amunts, 2020; Waltz *et al*, 2020). The availability of so many structures highlighted an interesting feature of mitoribosomes—their incredible evolutionary diversity (Waltz & Giegé, 2019; Kummer & Ban, 2021). The composition of mitochondrial ribosomes in different eukaryotic lineages underwent dramatic changes caused by multiple losses of RNA segments and mitoribosomal proteins (MRPs) as well as acquisition of new, lineage‐specific RNA segments and MRPs (Smits *et al*, 2007; Desmond *et al*, 2011; Sluis *et al*, 2015; Petrov *et al*, 2019). As a result, mitoribosomes contain a core set of MRPs homologous to the bacterial ribosomal proteins (BRPs) and a variable set of MRPs that can be common for all mitochondrial ribosomes or specific only to certain eukaryotic lineages. In addition, during their evolution, many MRPs acquired significant expansions of their C‐ and N‐termini while retaining structurally conserved domains of their BRP ancestors (Vishwanath *et al*, 2004; Sluis *et al*, 2015; Melnikov *et al*, 2018).

Mitochondrial genomes in many eukaryotic organisms still contain genes for a number of ribosomal proteins, indicating that their successful transfer to the nuclear genome might be less easily feasible than that of many other matrix proteins (Bertgen *et al*, 2020). However, most eukaryotic, and in Metazoa even all, MRPs are nuclear encoded. Thus, similar to the majority of mitochondrial proteins (numbering from around 800 in yeast to around 1500 in mammals), they must be imported from the cytosol (Pagliarini *et al*, 2008; Morgenstern *et al*, 2017; Vögtle *et al*, 2017).

The import of mitochondrial proteins can be conceptually subdivided in two steps: (i) targeting of the newly synthesized mitochondrial protein precursors to the mitochondrial membrane. This can occur either post‐translationally or co‐translationally involving ribosome‐nascent chain complexes. (ii) Translocation of the unfolded precursors through the mitochondrial membrane(s) to deliver them to their final destination within mitochondria (Bykov *et al*, 2020). Effective targeting and translocation are mediated by specialized protein complexes that recognize targeting and translocation signals within precursor protein sequences. Transport through the outer membrane is mediated by the TOM (translocon of the outer membrane) complex and through the inner membrane by TIM23 or TIM22 (translocon of the inner membrane) complexes (reviewed in Neupert & Herrmann, 2007).

Most matrix and inner membrane proteins are synthesized with N‐terminal matrix targeting sequences (MTSs), also called presequences, which are both necessary and sufficient for mitochondrial targeting. MTSs have a characteristic structure that can be predicted computationally (Claros & Vincens, 1996; Emanuelsson *et al*, 2000; Fukasawa *et al*, 2015; Armenteros *et al*, 2019). MTSs are typically between 10 and 60 residues in length and can form an amphipathic α‐helix with one positively charged surface and one hydrophobic surface. On the outer membrane, MTSs are recognized by the receptor subunits of the TOM complex, Tom20 and Tom22, and then threaded through the β‐barrel pore of Tom40. MTS‐containing proteins destined to the matrix are transported through the TIM23 complex that has two pore‐forming subunits Tim23 and Tim17 while some inner membrane proteins without MTS can get inserted via the TIM22 complex (reviewed in Neupert & Herrmann, 2007). In most cases, MTSs are proteolytically removed during protein import, giving rise to mature forms of mitochondrial matrix or inner‐membrane proteins (von Heijne, 1986; Bedwell *et al*, 1989; Vögtle *et al*, 2009).

In contrast to all other proteins of the mitochondrial matrix, many MRPs lack N‐terminal MTSs (Woellhaf *et al*, 2014). In some cases, MRPs use N‐terminal regions that mimic the properties of MTSs but are not cleaved (un‐cleaved MTSs). Such un‐cleaved MTSs are also found in some matrix proteins that are not associated with the ribosome, such as Hsp10 (Poveda‐Huertes *et al*, 2020). Surprisingly, a number of MRPs do not contain any regions that show MTS‐like features and it is unknown how mitochondria recognize and import these proteins. For now, there are only two well characterized examples of MRPs with unconventional MTSs—Mrpl32 (bL32m, by new nomenclature (Ban *et al*, 2014)) and Mrp10 (mS37) whose import path deviates from the canonical matrix‐targeting route (Nolden *et al*, 2005; Bonn *et al*, 2011; Longen *et al*, 2014).

In this work, we studied the mechanisms by which MRPs are targeted and translocated into mitochondria. We systematically examined N‐termini of unconventional MRPs and analyzed them *in silico* and experimentally. We further focused on the biogenesis of Mrp17 (bS6m) as a representative of the unconventional group of MTS‐less MRPs. We discovered a novel mitochondrial matrix targeting region that is displayed in the internal sequence of the protein. This stretch shares properties with mitochondrial targeting sequences such as positive charges for receptor binding and membrane potential‐dependent translocation, but differs in its structural features and position in the protein. The efficient import of Mrp17 shows that the mitochondrial import machinery is much more versatile in its substrate spectrum than expected. More generally, our work shows how structural restrictions favored the generation of unconventional targeting motifs.

## Results

### Mapping unconventional MRP targeting signals

**Figure EV1:**
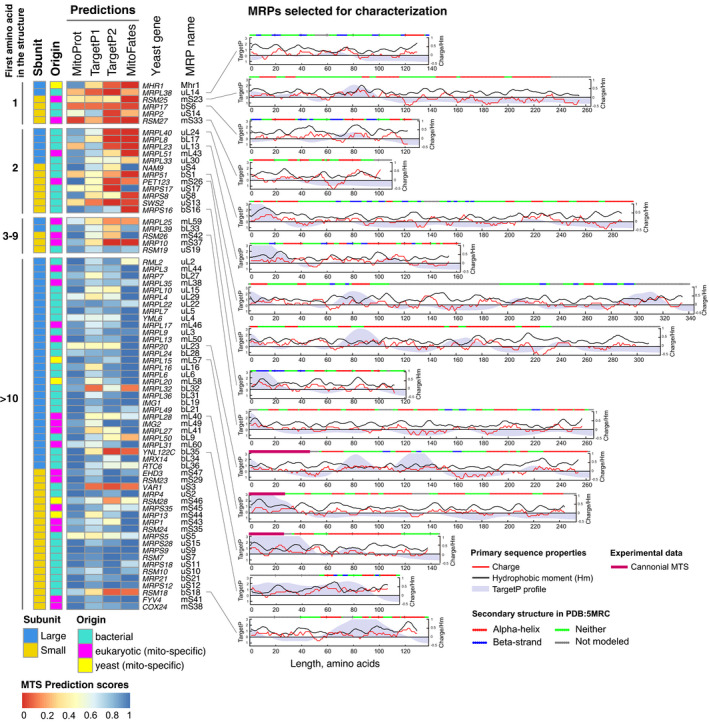
MRPs can be classified according to the presence of their most N‐terminus inside the mitoribosome structure Left—MTS prediction scores for yeast MRPs first sorted in groups by the first amino acid with reported atomic coordinates in the structure PDB:5MRC, then by subunit and then by length with protein origin and subunit noted for each MRP. Right—primary and secondary sequence properties for 15 MRPs selected for further characterization showing a variety of N‐terminal and internal targeting signal predictions, overall positive charge, presence of documented cleavable MTS, and a variety of N‐terminal secondary structures. Universal ribosomal protein nomenclature is used (Ban *et al,* 2014), except for Mhr1 which is a yeast‐specific MRP.

To systematically investigate MRP targeting signals in detail, we compiled all existing data on the maturation of their N‐termini in yeast (Dataset EV1). We used direct N‐terminal sequencing data (Graack *et al*, 1988, 1991; Grohmann *et al*, 1989, 1991; Matsushita *et al*, 1989; Dang & Ellis, 1990; Kitakawa *et al*, 1990, 1997; Boguta *et al*, 1992; Davis *et al*, 1992; Matsushita & Isono, 1993), N‐terminal proteomics (Vögtle *et al*, 2009) and predictions performed by UniProt annotators, as well as by ourselves using MitoFates for cleavage site prediction (Fukasawa *et al*, 2015). Importantly, we also used available structural information (Desai *et al*, 2017). In particular, mitoribosome structures were helpful to identify proteins that do not have a cleavable MTS—such proteins had their N‐termini contained within the structure and hence could not have been cleaved after import into the mitochondrial matrix. We reanalyzed ribosome profiling data on translation initiation in yeast to ascertain that none of these proteins has mis‐annotated translation start sites that might produce an N‐terminal extension accounting for a missing cleavable MTS (Appendix Fig S1). In the yeast mitochondrial ribosome structure (PDB:5MRC), the detectable sequence of six proteins started with amino acid number 1 (Met), that of 12 started with amino acid number 2, five—with amino acids 3–9, and the rest, 50, with amino acid number 10 and more. The number of the first amino acid present in the structure was moderately conserved among the determined mitoribosome structures (Appendix Fig S2) and was not restricted to any particular group of MRPs classified by origin (bacterial, mitochondria‐specific, or yeast‐specific) or position in the structure (Fig EV1, Appendix Fig S2).

**Figure 1:**
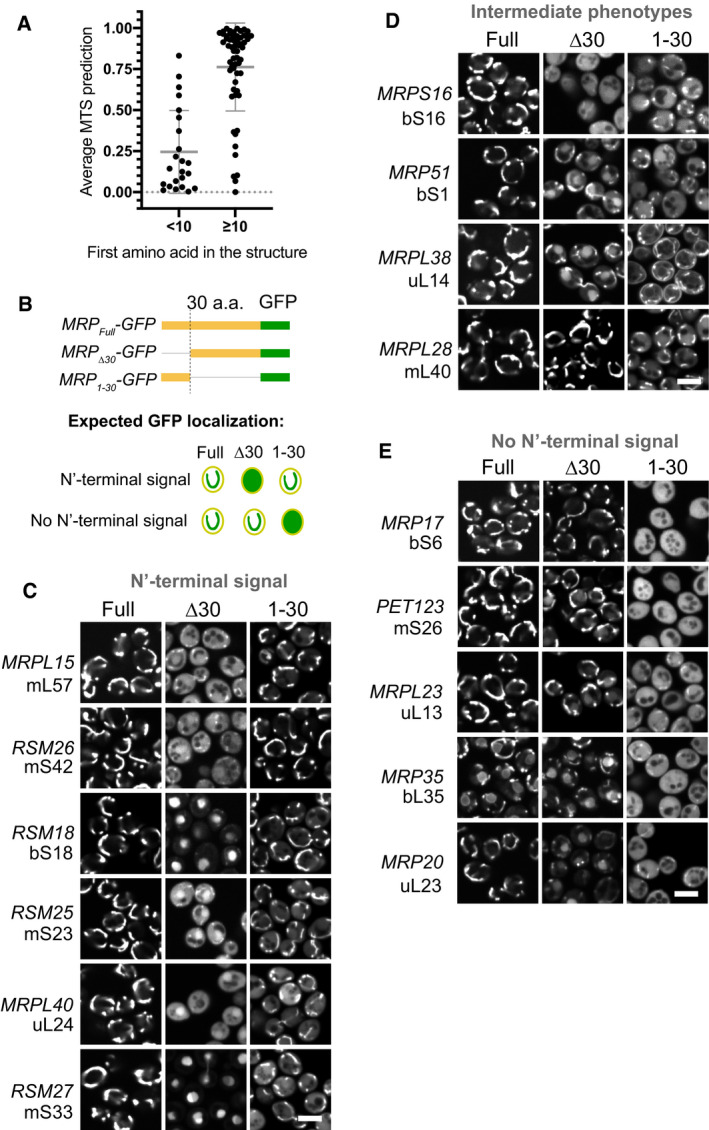
Mitochondrial ribosomal proteins (MRPs) have various types of targeting signals AYeast MRPs having uncleaved N‐termini that can be tracked in the mitoribosome structure (PDB:5MRC) score much lower with MTS prediction algorithms (average of TargetP2 and MitoFates) compared with other MRPs that have their N‐termini cleaved off or are not present in the structure (so might be flexible and outside the mitoribosome body). Mean ± SD is indicated by the bars, no replicates were performed as the values are predictions. See Fig EV1 for more detailed data.BSchematic of MRP truncations used to characterize targeting properties of MRP N‐termini: MRP_Full_ as control, MRP_∆30_ to check if the N‐terminus is necessary, MRP_1–30_ to check if it is sufficient (top) and the schematics of expected GFP localization in case the N‐terminus is MTS‐like (necessary and sufficient) or not (bottom).C–EMicrographs collected in the GFP channel for each truncation (columns) of each studied MRP (rows) grouped by the N‐terminus targeting properties based on theoretical expectation summarized in (B) with the MRPs possessing MTS‐like N‐termini in panel (C), MRPs with intermediate phenotype in panel (D) and MRPs without N‐terminal signal in panel (E), for each MRP a yeast gene name and new nomenclature protein name is shown on the left. Scale bar for all micrographs is 5 µm.

Interestingly, a simple distinction by the first amino acid appearing in the structure separates MRPs into two classes. In the first group are those MRPs that are derived from cleaved precursors (which consistently have high MTS prediction scores). In addition, this group may contain proteins with an uncleavable N‐terminus of a flexible nature which would then be unresolved in the available structures. Some of the latter may have poor mitochondrial targeting scores in prediction algorithms. In the second group are those whose structure starts with amino acid number less than 10. Most of these proteins score very poorly with different software predicting N‐terminal MTS (Figs 1A and EV1). Many MRPs of this group lack conventional, N‐terminal import signals, and their targeting signals are not predicted by available software. Thus, the available structures of mitochondrial ribosomes confirm the previous conclusion that many MRPs are made without N‐terminal MTSs (Woellhaf *et al*, 2014).

Next, we experimentally analyzed the targeting information in the sequences of different MRPs by GFP fusion proteins. To this end, we selected 15 MRPs with different properties (Fig EV1, Dataset EV2). Then we tested whether the N‐terminal 30 residues of these proteins were necessary and/or sufficient for mitochondrial targeting. The length of 30 residues was chosen as it corresponds to the most common size of a cleavable yeast MTS (Vögtle *et al*, 2009). To test this, we expressed each MRP in diploid yeast fused to GFP. To assay if the N‐terminus is necessary, we expressed a truncated version with the first 30 amino acids deleted (MRP_type="InMathematical_Operators">∆30_‐GFP). To test if the N‐terminus is sufficient, we expressed a version with only the first 30 amino acids (MRP_1–30_‐GFP). As a control, we used the full‐length version (MRP_Full_‐GFP; Fig 1B). The distribution of GFP signals was imaged in cells in which mitochondria were stained with MitoTracker Orange (Fig 1C–E, Appendix Fig S3, Dataset EV2). Six proteins (Mrpl15, Rsm26, Rsm18, Rsm25, Mrpl40, and Rsm27) contained targeting information within their N‐termini (Fig 1C); of them, only Mrpl15 (mL57) had high MTS prediction scores consistent with highly confident annotation of a cleavable 29‐amino acid long MTS (Dataset EV1). Other proteins whose N‐termini were able to target GFP to mitochondria had low MTS prediction scores (Dataset EV2) indicating that their N‐terminal signals have distinct properties, not similar to conventional MTSs. For four proteins (Mrps16, Mrp51, Mrpl38, and Mrpl28) neither the N‐terminal 30 residues nor the internal segment on its own were sufficient for targeting, indicating that the necessary targeting information is contained in an N‐terminal segment longer than 30 amino acids or distributed over the whole length of these proteins (Fig 1D). Finally, five proteins (Mrp17, Pet123, Mrpl23, Mrp35, and Mrp20) were targeted to mitochondria independently of their N‐terminal regions indicating that the targeting signals in these proteins are internal (Fig 1E).

**Figure EV2:**
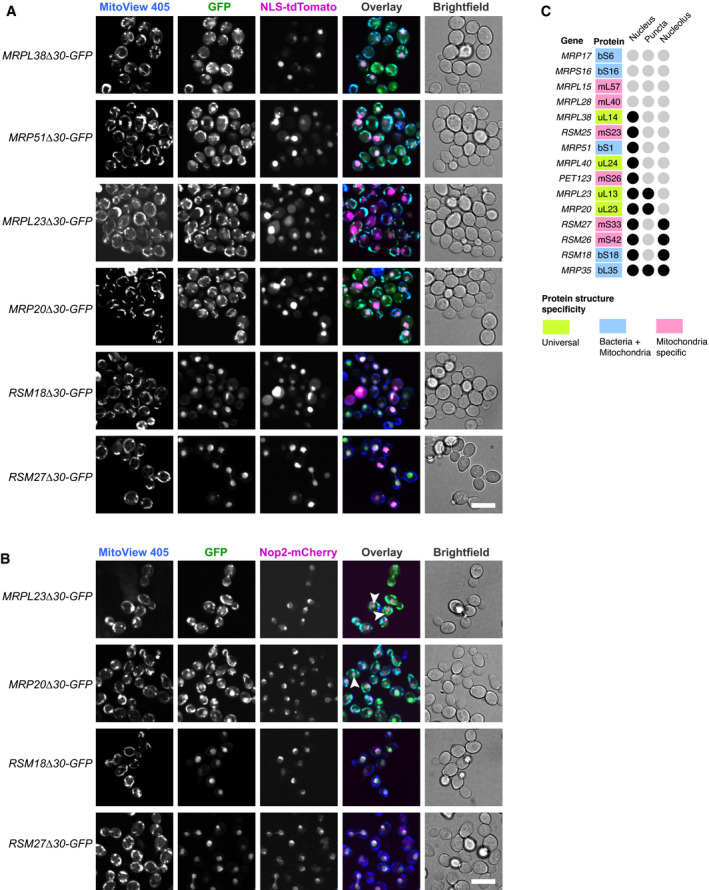
MRPs have N‐termini with various targeting properties MRP‐GFP truncations mistargeted to the nucleus were transformed with NLS (nuclear localization signal)‐tdTomato plasmid, stained with MitoView 405 dye and visualized by fluorescent microscopy. Scale bar is 10 µm.MRP‐GFP truncations mistargeted to the nucleus and enriched in the nucleolus with nucleolar protein Nop2 genomically tagged using mCherry, stained with MitoView 405 dye and visualized by fluorescent microscopy. Observed MRP‐GFP aggregates are highlighted with white arrowheads. Scale bar is 10 µm.Summary of mistargeting locations for one or more truncations of each MRP, if the location is observed for any of the MRP truncations, it is marked with a black circle.

Interestingly, many of the N‐terminally truncated MRP versions accumulated outside mitochondria in the cytosol or, in many cases, in the nucleus and nucleolus (Figs 1C–E and EV2). These observations agree with the recent discovery that mistargeted mitochondrial proteins can accumulate in the nucleus and get degraded in perinuclear puncta (Shakya *et al*, 2021). Despite the mislocalization of several of these forms, none of them resulted in obvious growth defects (Appendix Fig S4).

To summarize, we selected a subset of MRPs with diverse structural and sequence features and characterized the mitochondrial targeting capacity of their N termini. We observed that many of these MRPs contain unconventional targeting signals, often outside of the 30 N‐terminal residues, and apparently scattered over their sequence. One particularly intriguing MRP was Mrp17 (bS6m), a protein of the small subunit of the yeast mitoribosome. Mrp17 lacks any identifiable targeting signal and is present in all mitoribosome structures studied to date with its N‐terminus visualized in all of these structures (Appendix Fig S2). Hence, we chose Mrp17 for further investigation.

### Defining Mrp17 targeting and translocation signals

**Figure EV3:**
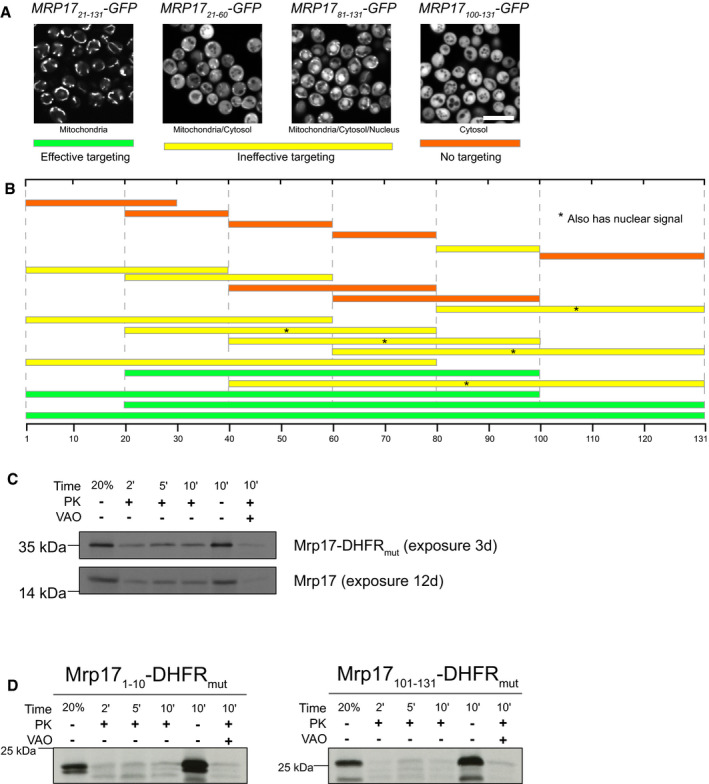
The noncanonical targeting and translocation signal of Mrp17 is located between amino acids 30 and 60 A, B
*In vivo* characterization of mitochondrial targeting capacity of different Mrp17 truncations fused to GFP: (A) GFP localization examples for truncations, and demonstrating effective targeting with only mitochondrial GFP signal (Mrp17_21–131_‐GFP, same data as in Appendix Fig S5); ineffective targeting with mitochondrial GFP signal accompanied by strong cytosolic signal (Mrp17_21–60_‐GFP, same data as in Fig 2A and Appendix Fig S5); ineffective targeting with additional nuclear signal (Mrp17_81–131_‐GFP, same data as in Appendix Fig S5); and no detectable mitochondrial targeting with exclusively cytosolic GFP (Mrp17_100–131_‐GFP, same data as in Appendix Fig S5). Scale bar is 10 µm. (B) localization summary of different Mrp17 truncations fused to GFP and colored according to the color‐code for effective, ineffective, and no targeting introduced in panel (A), truncations additionally targeted to the nucleus are marked with asterisks.CMrp17‐DHFR_mut_ is translocated into isolated mitochondria at the same rate as WT Mrp17 but gives better signal in the autoradiograph.D
*In vitro* import assays for additional truncations of Mrp17 fused to DHFR_mut_ not shown in Fig 2B, import was performed as described in the legend for Fig 2.

**Figure 2:**
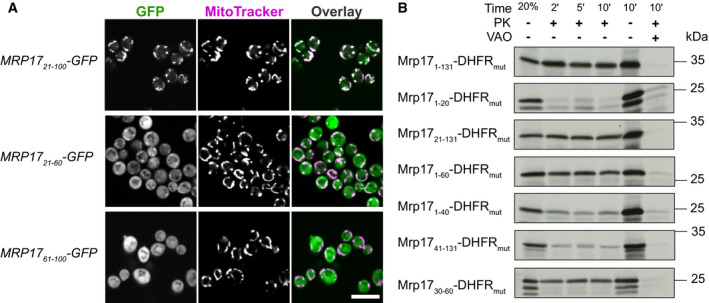
The noncanonical targeting and translocation signal of Mrp17 is located between amino acids 30 and 60 *In vivo* characterization of mitochondrial targeting capacity of different Mrp17 truncations fused to GFP visualized by fluorescent microscopy with MitoTracker Orange straining. Scale bar for all micrographs is 10 µm.Characterization of Mrp17 translocation signal using an *in vitro* import assay: shown are autoradiographs of full‐length Mrp17 or its truncations fused to DHFR_mut_, translated *in vitro* with radiolabeled amino‐acids, incubated with isolated yeast mitochondria for 2, 5, or 10 min, treated with proteinase K (PK) to remove nonimported proteins and visualized by 16% SDS–PAGE/autoradiography. As a negative control, mitochondria were treated with valinomycin, antimycin, and oligomycin (VAO) that eliminate membrane potential. For comparison, 20% of the protein used per import reaction was loaded on the first lane.

To investigate the unconventional targeting signals of Mrp17 in more detail, we created a systematic set of Mrp17 truncations fused to GFP and expressed them in diploid yeast (Fig EV3, Appendix Fig S5). We observed that the internal fragment of Mrp17 between amino acids 20 and 100 was the minimal fragment able to target GFP to mitochondria similarly to full‐length Mrp17 (131 amino acids) without producing cytosolic background signal (Fig 2A). This indicates that similarly to the N‐terminus, the C‐terminus is dispensable for targeting. Splitting this fragment in two halves showed that the N‐terminal part (Mrp17_21–60_) was still able to target GFP to mitochondria although with significant cytosolic background while the C‐terminal part (Mrp17_61–100_) was cytosolic (Fig 2A). We conclude that, *in vivo,* Mrp17 region 21–60 is necessary for mitochondrial targeting but is not sufficient for efficient targeting, which is promoted by additional signals distributed over the whole length of the protein (Fig EV3A and B).

The microscopic analysis does not allow us to discriminate between targeting to the mitochondrial surface from complete translocation into the matrix and is affected by truncated MRP stability *in vivo*. To elucidate the translocation efficiency of different Mrp17 regions, we used *in vitro* import assays into isolated yeast mitochondria. Since Mrp17 is very small and many fragments lacked methionine residues that are necessary for radiolabeling, we fused Mrp17 to an unfolded mutant of the mouse dihydrofolate reductase —DHFR_mut_ (Vestweber & Schatz, 1988). The full‐length Mrp17‐DHFR_mut_ fusion was effectively imported into isolated yeast mitochondria at the same rate as untagged Mrp17 but gave much stronger signals in autoradiography (Figs 2B and EV3C). In agreement with the targeting experiments performed *in vivo*, the short N‐terminal region of Mrp17 was neither necessary nor sufficient for efficient translocation (Mrp17_21–131_, Mrp17_1–20_ in Fig 2B). The first 60 amino acids of Mrp17 were sufficient for translocation narrowing down the import signal to the N‐terminal half of the protein (Mrp17_1–60_ in Fig 2B). Leaving only the first 40 amino acids or removing them from the N‐terminus reduced the translocation speed indicating that regions 20–40 and 40–60 are equally important parts of the signal (Mrp17_1–40_ and Mrp17_41–131_ in Fig 2B). Finally, we narrowed down the Mrp17 region containing the translocation signal to amino acids 30–60 (Fig 2B, bottom; Fig EV3D). However, similar to the results of *in vivo* experiments, even short fragments of Mrp17 outside this region retained some residual translocation capacity (Fig 2B).

To summarize, we determined that the main mitochondrial targeting and translocation signal of Mrp17 is positioned between amino acids 30 and 60. However, there exist additional signals that improve mitochondrial targeting efficiency or stability *in vivo*. These additional signals reside in the C‐terminal half of Mrp17. This again indicates, that the mitochondrial targeting regions are scattered over the Mrp17 sequence, and for this protein, the most N‐terminal region is irrelevant for efficient mitochondrial import.

### Characterizing features of the Mrp17 targeting and translocation signal

**Figure 3:**
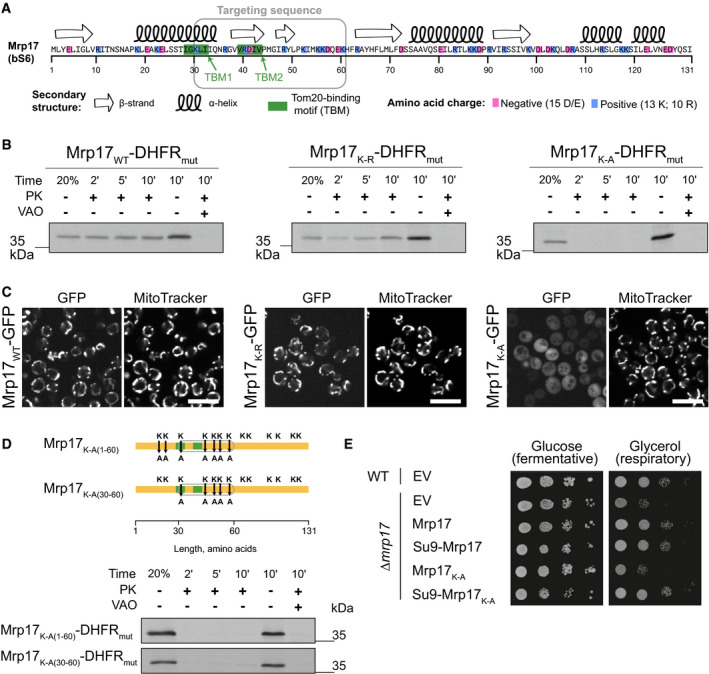
Positive charge and Tom20‐binding motifs are important features of the Mrp17 targeting signal Primary sequence of Mrp17 highlighting charged amino‐acids, Tom20‐binding motifs (TBM1, TBM2) and secondary structure (from PDB:5MRC).
*In vitro* mitochondria translocation capacity of WT Mrp17, Mrp17_K‐R_ with all lysines (K) substituted with arginines (R), and Mrp17_K‐A_ with all lysines (K) substituted with alanines (A) fused to DHFR_mut_. Import was performed as described in Fig 2 legend.
*In vivo* mitochondrial targeting capability of WT Mrp17, Mrp17_K‐R_, and Mrp17_K‐A_ fused to GFP. Scale bar is 10 µm.Substitution of lysines with alanines in the regions 1–60 or 30–60 of Mrp17 abolishes mitochondrial import capacity: (top) schematics of substitution positions, TBMs are in green, targeting signal outlined in grey, substitutions are denoted by arrows; (bottom) *in vitro* translocation of the mutants.Mrp17 variants rescue *∆mrp17* strain growth defect on respiratory media: the indicated variants or empty vector (EV) were introduced in WT yeast and then the genomic *MRP17* was disrupted by knock out, the resulting mutants were serially diluted 10× and spotted on media containing glucose or glycerol as a sole carbon source.

**Figure EV4:**
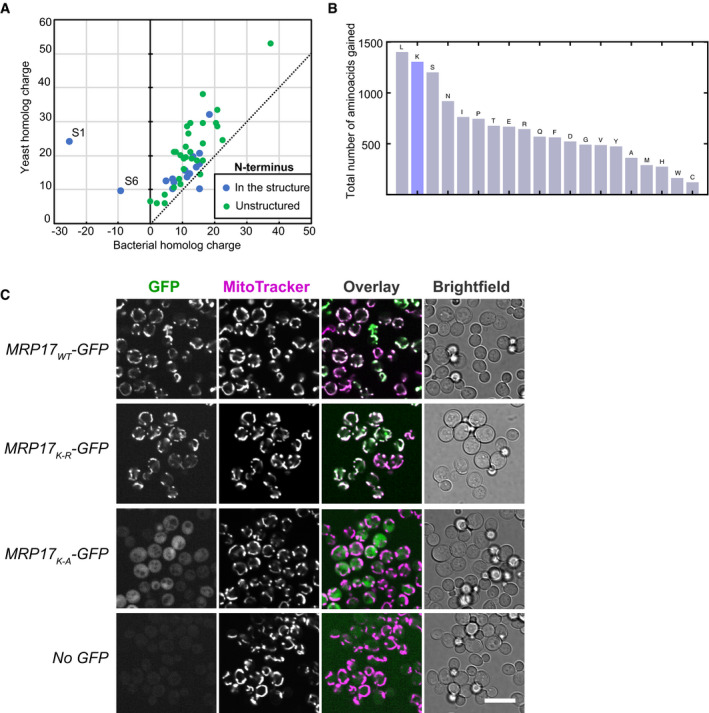
Mrp17 sequence features important for targeting and translocation to mitochondria Ribosomal proteins are positively charged and mitochondrial proteins acquired even more positive net charge compared with their bacterial homologs.Total amino acid gain of MRPs (calculated as the difference between total count of each amino acid in all yeast MRPs, including mitochondria‐specific, and all bacterial RPs) compared with bacterial RPs shows over‐representation of lysines (K).Lysines in Mrp17 are not important for mitochondrial targeting and can be substituted with arginines, same micrographs for constructs MRP17_WT_‐GFP, MRP17_K‐R_‐GFP, and MRP17_K‐A_‐GFP as in Fig 3C shown in all channels beside micrographs of yeast not expressing any GFP (bottom row) as control for autofluorescence relative to cytosolic signal. All micrographs in the GFP channel are shown at the same contrast and brightness for comparison; Scale bar is 10 µm.

Next, we analyzed the unconventional internal targeting region of Mrp17 located between residues 30 and 60 in more detail. Standard prediction algorithms do not find an MTS‐like sequence in this region (Fig EV1). The Mrp17 structure mostly contains β‐strands in this region and only a part of a helical stretch (Fig 3A). Mrp17 is generally rich in positive charges (its pI is 10.5). While a high content of positive charges is a general feature of ribosomal proteins that interact with negatively charged mRNA, during evolution, the content of positive charges in MRPs (and particularly their lysine content) was further increased. This suggests that positive charges might play a role beyond their relevance for neutralizing the negative charges of ribosomal RNA (Fig EV4A and B).

To find out whether mitochondrial targeting and import require the positive charges and if so, whether there is a specific dependence on lysines, we constructed mutants of Mrp17 with all lysines substituted with arginines thus maintaining the charge (Mrp17_K‐R_) or all lysines substituted for alanines thus decreasing the charge (Mrp17_K‐A_). The Mrp17_K‐R_ mutant fused to DHFR_mut_ was efficiently imported into isolated mitochondria, while the Mrp17_K‐A_ mutant was not (Fig 3B). Similarly to the results obtained *in vitro*, Mrp17_K‐R_ fused to GFP and expressed in yeast colocalized with mitochondria as the wild‐type Mrp17 while Mrp17_K‐A_‐GFP remained cytosolic (Figs 3C and EV4C). Thus, the positive charge is an important feature of the Mrp17 targeting and translocation signal.

To check if the presence of positive charges is specifically required in the translocation signal, we constructed Mrp17 mutants where lysines were substituted with alanines only in the region of the first 60 amino acids (Mrp17_K‐A(1–60)_) or only within amino acids 30–60 (Mrp17_K‐A(30–60)_). These mutants fused to DHFR_mut_ were also not imported indicating that the positive charges indeed must be positioned in the targeting signal region (Fig 3D).

To check if Mrp17 lysines also play other important functions, we expressed Mrp17_K‐A_ and its variants in *∆mrp17* cells. Consistent with the import defect, Mrp17_K‐A_ was not able to rescue *∆mrp17* yeast growth on respiratory media. However, when we restored the mutant import by fusing it with strong cleavable MTS from *Neurospora crassa* ATP‐synthase subunit 9 (Su9), the resulting fusion Su9‐Mrp17_K‐A_ was able to substitute for WT Mrp17 (Fig 3E). This shows that lysines are important for correct import of Mrp17 but are dispensable for ribosome function. Another interesting implication of this result is that Mrp17 N‐terminus can be adjusted to harbor a cleavable MTS without perturbing mitoribosome function.

In summary, by using mutagenesis, we determined that positive charge, most critically between amino‐acids 30 and 60, is important for Mrp17 targeting and translocation but not for its function.

### Mrp17 uses a similar translocation route into mitochondria as MTS containing proteins

**Figure 4:**
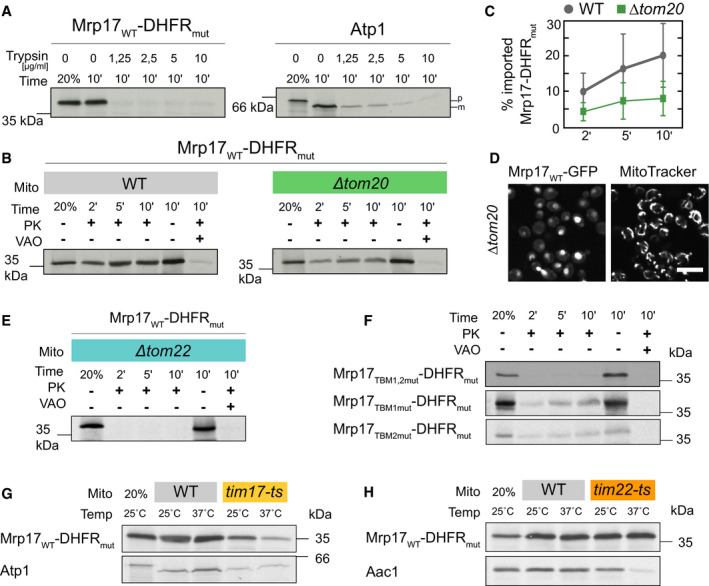
Mrp17 uses a similar translocation route into mitochondria as MTS containing proteins AThe *in vitro* import of Mrp17‐DHFR_mut_ is sensitive to the elimination of outer mitochondrial membrane proteins by trypsinization (left), more so than the model import substrate Atp1 (right). Mitochondria were incubated with the indicated concentrations of trypsin and the import assay was performed as described in the legend for Fig 2. Proteinase K (PK) was not added to the sample without mitochondria.B
*In vitro* translocation of Mrp17‐DHFR_mut_ into mitochondria isolated from WT and *∆tom20* yeast showing reduced translocation in *∆tom20* background.CQuantification of the experiment in panel (B) repeated three times, whiskers correspond to minimum and maximum values.DExpression of Mrp17‐GFP in *∆tom20* yeast shows mislocalization to the nucleus compared with WT cells (Full micrographs and WT control shown in Appendix Fig S6A). Scale bar is 10 µm.EImport of Mrp17‐DHFR_mut_ into purified *∆tom22* mitochondria, control import into WT mitochondria is shown on Appendix Fig S6B.FSubstitution of both Tom20‐binding motifs (TBM) with alanines (TBM1,2mut) abolishes Mrp17 mitochondrial import capacity while substituting of a single motif (TBM1mut, TBM2mut) reduces import.G, HImport of Mrp17‐DHFR_mut_ and control proteins Atp1 and Aac1 into WT, *tim17‐ts* (G), and *tim22‐ts* (H) mitochondria. Import was performed at indicated temperatures, proteinase K was added to all samples except the loading control in the first lane of each autoradiograph (20% of protein amount used for each import reaction). The full autoradiograph for Aac1 with molecular weight markers is shown in Appendix Fig S6D. Source data are available online for this figure.

Since Mrp17 lacks the typical features and targeting signals of matrix proteins, we wondered how this protein was first recognized by import receptors on mitochondrial surface and later imported. To test the receptor requirement for the mitochondrial import of Mrp17, we treated isolated yeast mitochondria with trypsin that removes all the import receptors of the TOM complex from the mitochondrial surface but spares membrane‐embedded TOM subunits (Ohba & Schatz, 1987). Trypsin treatment strongly inhibits the import of MTS containing matrix proteins such as Atp1 (Fig 4A) but does not affect the import of most intermembrane space (IMS) proteins (Lutz *et al*, 2003; Gornicka *et al*, 2014). The import of Mrp17‐DHFR_mut_ was even more sensitive to trypsin treatment than the import of the well‐studied MTS‐containing protein Atp1 (Fig 4A). Thus, Mrp17 import into the mitochondrial matrix strongly depends on the presence of the TOM receptors.

To determine the dependence of Mrp17 import on individual receptors, we purified mitochondria from yeast lacking Tom20 (∆*tom20*), the main receptor for recognition of canonical MTSs. Mrp17‐DHFR_mut_ import into *∆tom20* mitochondria was strongly reduced (Fig 4B and C) indicating that Tom20 is involved in the import process. Consistent with this result, Mrp17‐GFP expressed in *∆tom20* cells was mislocalized to the nucleus (Fig 4D, Appendix Fig S6A). Deletion of Tom22, another receptor that takes part in MTS recognition, also abolished Mrp17‐DHFR_mut_ import into isolated mitochondria (Fig 4E, Appendix Fig S6B). However, this import defect can also be explained by the general defect in TOM complex assembly which requires the transmembrane domain of Tom22 (Bausewein *et al*, 2017). Deletion of Tom70 and its lowly expressed paralog, Tom71, had no effect on the import of Mrp17‐DHFR_mut_ (Appendix Fig S6C), consistent with the absence of internal MTS‐like sequences (iMTS‐Ls, (Backes *et al*, 2018)) in the protein (Fig EV1).

Since the structural basis of MTS recognition by Tom20 is well studied it is possible to predict Tom20‐binding motifs (TBM) in precursors. TBMs comprise a hydrophobic amino acid followed by two amino acids and ending with two hydrophobic ones (Muto *et al*, 2001; Obita *et al*, 2003). We found that Mrp17 contains two such predicted Tom20‐binding motifs (TBD1 and TBD2, Fig 3A) in the area required for targeting and translocation. To test whether the requirement for Tom20‐binding motifs might be direct, we constructed a mutant where each amino acid of either one (Mrp17_TBM1mut_, Mrp17_TBM2mut_) or both (Mrp17_TBM1,2mut_) Tom20‐binding motifs was substituted with alanine. While mutation of individual binding sites reduced the import efficiency, the mutation of both fully abrogated Mrp17 import (Fig 4F). Thus, these two short Tom20‐binding sites (residues 28–32 and 39–44) provide Mrp17 the property to bind the mitochondrial surface and to be recognized by the import machinery.

Next, we used the temperature‐sensitive mutants *tim17‐ts* and *tim22‐ts* that reduce the protein import along either of the two TIM pathways—the TIM23 and TIM22 pathways, respectively. Mrp17 import into *tim17‐ts* mitochondria was reduced, whereas its import into *tim22‐ts* mitochondria was not affected showing that Mrp17 uses the TIM23 pathway, similar to canonical MTS‐containing proteins (Fig 4G and H).

To summarize, Mrp17 has an internal targeting signal that shares some properties such as positive charge with a regular MTS. Indeed, its import pathway is similar to MTS‐containing proteins suggesting that mitochondrial components recognize it as a *bona fide* MTS yet even state‐of‐the‐art prediction algorithms do not, suggesting that we lack information on certain MTS characteristics.

### Evolution of Mrp17 targeting propensity

**Figure 5:**
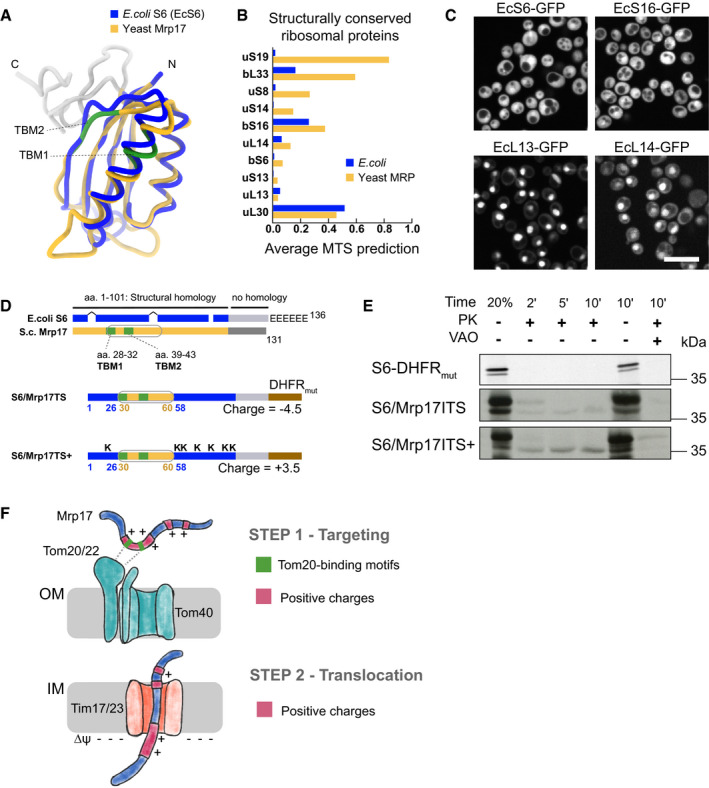
Bacterial homologs of MRPs have no mitochondrial import predisposition AStructural alignment of the yeast Mrp17 (from PDB:5MRC) and its *Escherichia coli* homolog ribosomal protein S6 (from PDB: 6WD0) viewed from its cytosol‐facing side, highlighting regions of structural homology (blue and yellow) and nonhomologous C‐terminal regions (grey), Tom20‐binding domains in Mrp17 are highlighted in green, schematic summaries of such structural alignments for all MRPs and their bacterial homologs are shown in Appendix Fig S7.BAverage MTS prediction scores (MitoFates and TargetP2) for structurally conserved yeast MRPs and their bacterial homologs sorted by the difference between bacteria and mitochondria.CExpression of structurally conserved bacterial RPs fused to GFP in yeast cells (Full micrographs in al channels are shown on Fig EV5). Scale bar is 10 µm.DSchematic summary of structural alignment of bacterial S6 and yeast Mrp17 shown in panel (A) highlighting internal targeting signal (grey outline), additional features of Mrp17 structure (two TBMs), and six glutamate residues (E's) at the C‐terminus of bacterial S6 (top); schematic of chimeric constructs of EcS6: S6/Mrp17ITS with Mrp17 amino acids 30–60 incorporated instead of the homologous region of S6 and fused to DHFR_mut_ (middle), S6/Mrp17ITS^+^ that is similar to S6/Mrp17ITS but has seven amino acids mutated to lysines (bottom).EChimeric variants of S6 depicted in panel D imported into isolated yeast mitochondria. Lanes are labeled as described in Fig 2 legend.FMrp17 targeting and translocation model that requires Tom20‐binding motifs and positive charges for targeting (STEP 1) and relies on ubiquitous positive charges for translocation (STEP 2).

Unlike many other core MRPs that significantly extended their structures with insertions and N/C‐terminal expansions, Mrp17 maintained the overall structure of its bacterial ancestors (Fig 5A, Appendix Fig S7). This means that in the course of evolution, the acquisition of an N‐terminal MTS was either complex so that the Mrp17 targeting signal had a higher chance to be accommodated within the existing “bacterial” structure, or the ribosomal protein was already predisposed for mitochondrial import. Since RNA‐binding regions show similarity to MTSs by both requiring positively charged and hydrophobic amino acids, import predisposition was an appealing hypothesis.

**Figure EV5:**
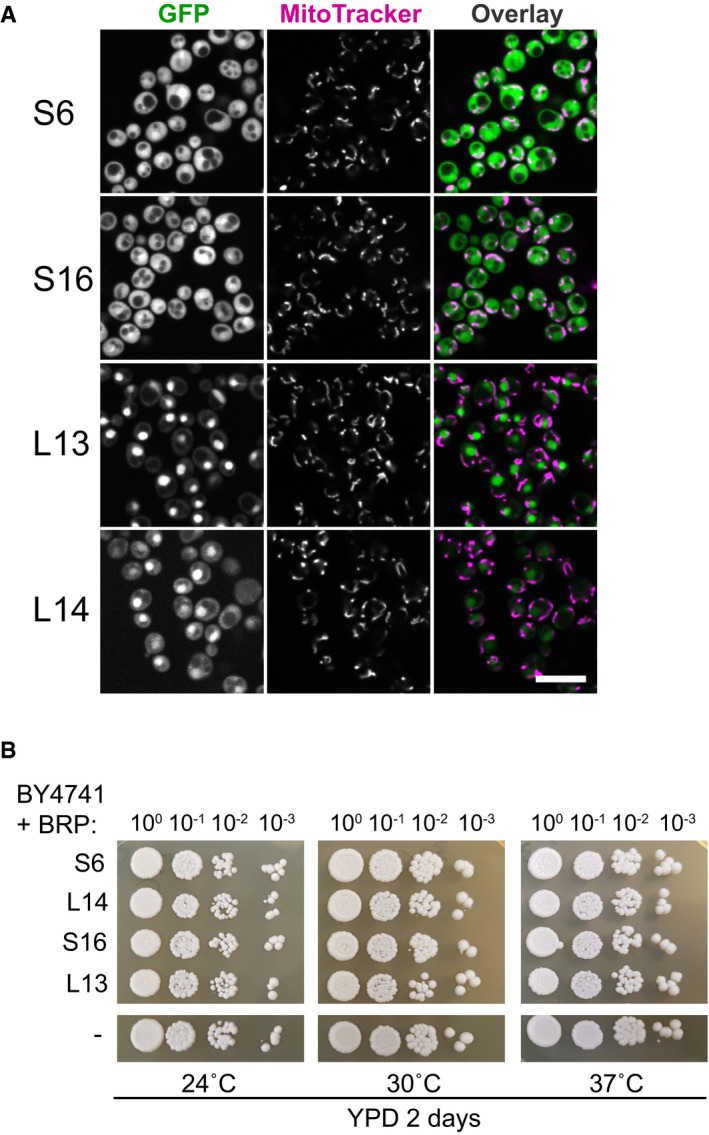
Comparison of Mrp17 and its bacterial homolog Expression of bacterial homologs of MRPs in yeast, same micrographs as in Fig 5C shown in all channels.Drop dilution growth assay for all the strains from panel A and WT control performed on rich fermentative media at different temperatures.

To investigate mitochondrial import predisposition, we compared average MTS prediction scores of Mrp17 (and other structurally conserved MRPs) to their closest bacterial homologs with known structure, that is, *Escherichia coli* ribosomal proteins (Figs 5B, Appendix Fig S7). Two out of the 10 structurally conserved proteins that lacked cleavable MTS indeed had much higher MTS prediction scores in mitoribosomes compared with bacterial ribosome meaning that uncleavable MTS‐like signals were developed at their N‐termini. For other proteins, both homologs had equally low scores (< 0.5) indicating that some sequence properties of bacterial and mitoribosomal proteins are similar. Next, we tested if these bacterial proteins already have properties that allow them to be imported into mitochondria *in vivo* by expressing them as GFP fusions in yeast (Figs 5C and EV5A). None of the bacterial proteins localized to mitochondria showing that these BRPs do not have an intrinsic mitochondrial targeting capacity and that targeting signals had to have been incorporated into conserved MRP structures in the course of evolution. Interestingly, two of the proteins (EcL13 and EcL14) had an intrinsic nuclear targeting capacity which was tolerated by the cells (Fig EV5B).

We next used the S6 sequence to test which features need to be added to make this protein import‐competent (Fig 5D). First, we integrated the internal targeting & translocation signal (ITS) of Mrp17, that is, its residues 30–60 into the homologous region of S6. The resulting construct S6/Mrp17ITS was imported into mitochondria, albeit with low efficiency (Fig 5E). This indeed shows that the internal Mrp17 segment between residues 30 and 60 serves as a matrix‐targeting signal that is sufficient to promote protein import. Next, we added lysine residues in the regions flanking the 30–60 stretch at positions at which these positive charges are found in Mrp17. This construct, S6/Mrp17ITS+, was now imported with improved efficiency (albeit not as efficiently as Mrp17; Fig 5E). Adding only Tom20‐binding motifs even in combinations with other small structural features of Mrp17 did not promote S6 targeting to mitochondria *in vivo* emphasizing the importance of the overall protein charge for targeting and translocation (Appendix Fig S8). Thus, the generation of the internal unconventional targeting signal in Mrp17 and the addition of lysines apparently were crucial steps in its BRP‐to‐MRP transition.

We suggest that both Tom20‐binding motifs and their flanking positive charges are important components of the Mrp17 ITS, while overall positive charge of the protein can be the driver of efficient translocation to the mitochondrial matrix (Fig 5F).

## Discussion

In this work, we collected the available information on the MTSs of yeast MRPs from proteomic and structural studies as well as MTS prediction algorithms. Using *in vivo* structure‐function assays, we found that some MRPs possessed internal targeting signals that can be poorly predicted. We characterized the internal targeting signal of Mrp17 in more detail and found that it shares some features, as well as its import pathway, with regular MTS‐containing proteins.

Why would Mrp17 and other MRPs rely on internal signals instead of evolving a canonical N‐terminal MTS? For Mrp17 and other MRPs that originated from bacterial ancestors and have not acquired any additional sequence extensions—presequences may have been simply not needed as these BRPs already possessed mitochondrial import capacity due to their positive charge and hydrophobicity that are needed to bind ribosomal RNA. Such intrinsic import capacity was indeed shown for some *E. coli* proteins (Lucattini *et al*, 2004). However, in our work even strongly positively charged *E. coli* ribosomal proteins S12, S16, L13, and L14, although structurally conserved with their MRP homologs, were not targeted to mitochondria when expressed in yeast suggesting no initial targeting predisposition (Fig 5C). Instead, we propose that the N‐termini of these proteins were so conserved that the chance of them being extended with a cleavable MTS by the process of incremental sequence evolution was very low. So proteins, such as Mrp17, ended up accommodating a targeting signal elsewhere in their sequences.

**Figure 6:**
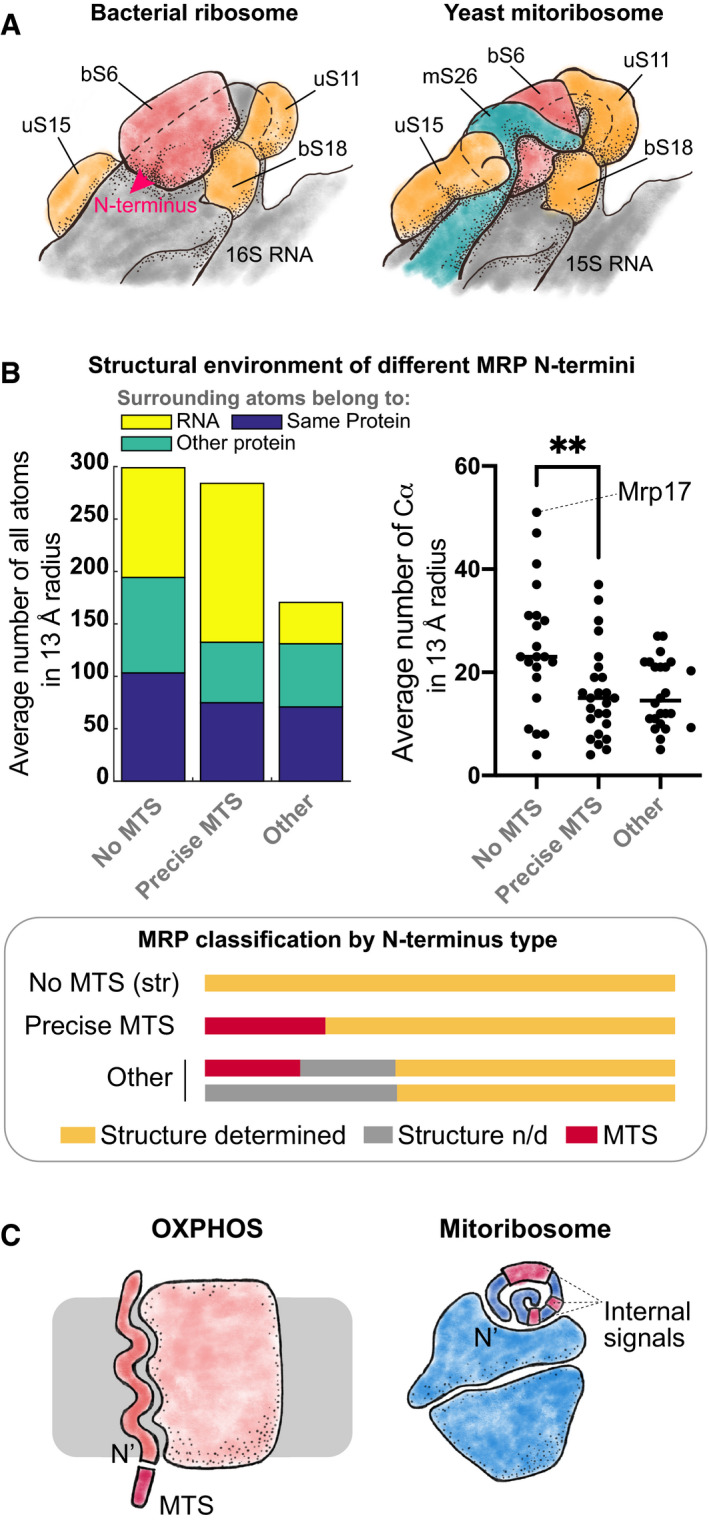
Structural constraints for the evolution of N‐terminal mitochondrial targeting signals ASchematic depiction of the S6 protein (red) in the context of bacterial ribosome (after PDB:6WD0, left) and yeast mitochondrial ribosome (after PDB:5MRC, right) highlighting the position of the N‐terminus of S6 (red arrow, only shown on the left image). Proteins homologous between bacterial and yeast structures are shown in orange, mitochondria‐specific protein mS26 in teal and rRNA in grey.BOn the right: MRP N‐termini structural environment (solvent exposure): average number of atoms belonging to the same protein, other protein chains or RNA in the 13 Å radius from the first structured amino acid Cα atom, plotted separately for proteins with their most N‐terminus appearing in the structure vs proteins with precisely cleaved MTS, and other proteins which have a longer unstructured extension (see the classification legend on the bottom) calculated based on PDB:5MRC; on the right: same as on the left but only coordination number (number of protein Cα atoms in the 13 Å radius (Hamelryck, 2005)) is calculated, ** *P*‐value < 0.01 (Mann–Whitney test), absolute values were measured without replicates; on the bottom: MRPs were divided in three groups depending on relative position of the MTS and structured residues: “No MTS (str)”—structure starts at the very N‐terminus without a cleavable MTS, “Precise MTS”—MTS is cleaved off 1—2 amino acids before the structure starts, “Other”—unstructured uncleaved amino acids present before the structure start.COXPHOS complex components usually have their N‐termini free to develop an MTS (left). In contrast, mitoribosomes often employ the MRP N‐termini in binding interfaces which puts structural constraints on the development of N‐terminal MTS and promotes the evolution of internal targeting sequences that can also take part in ribosome assembly (right).

Why would the evolution of an N‐terminal MTS be such an evolutionary barrier for Mrp17? The N‐terminus of Mrp17 is tightly positioned on the contact with neighboring proteins and this interface is conserved between yeast and humans (Fig 6A, Appendix Fig S9A). If this interface arose before the final maturation of the mitochondrial protein import system, it would create challenges for the evolution of an N‐terminal targeting signal. Such an engagement of the N‐terminus in a structural interface can still be kept with a cleavable MTS since the deletion of Mrp17 can be rescued with Su9‐Mrp17 construct (Fig 3E). Indeed, some MRPs have evolved in that way (Appendix Fig S7). We hypothesize that for Mrp17, evolution took a different path to avoid an intermediate evolutionary step which would have created a sub‐optimally cleaved N‐terminus with few extra amino acids that could not be accommodated in the structure. Instead, Mrp17 developed internal targeting signals and remodeled the charge of the whole protein which was favored by its RNA‐binding nature. Interestingly, the main signal identified in our work was not positioned in structurally distinct areas that are different between Mrp17 and EcS6 (Fig 5A and D; loop expansions, C‐terminal region) but mostly distributed between amino acids 30 and 60 which form a lot of new protein‐protein contacts within the mitoribosome compared with the bacterial ribosome (Appendix Fig S9A and B). So, the Mrp17‐targeting signal might have co‐evolved with protein‐protein interactions which is similar to the evolution of nucleolar‐targeting signals in cytosolic ribosomal proteins (Melnikov *et al*, 2015).

Can structural constraints also explain why other MRPs did not develop cleavable MTSs? To assay that, we divided all MRPs into three groups according to the relative position of their MTS cleavage site (if present) and the first structured residue (Fig 6B, legend on the bottom). We hypothesized that despite being involved in structure formation, the proteins that developed an MTS precisely cleaved off before the first structured residue might have N‐termini that are less buried in the structure, which could provide some flexibility for MTS cleavage evolution compared with the proteins that failed to develop an MTS and have a deeply buried N‐terminus, like Mrp17. However, this was not the case and these two groups had their N‐termini equally deeply buried in the structure (Fig 6B, left, “No MTS” and “Precise MTS” groups). The validity of this measurement was confirmed by the fact that the MRP N‐termini with few more unstructured residues before the structure start are indeed more often positioned on the ribosome surface (Fig 6C, “Other” group). Thus, there is no strict limitation for a buried N‐terminus to develop an MTS.

Interestingly, we found that the N‐termini without a cleavable MTS had more protein and less RNA around them in the ribosome structure with Mrp17 being an extreme example (Fig 6B, right). This suggests that unlike protein‐protein contacts, protein‐RNA contacts can be more permissible to the change in the protein component that will allow the adjustment of the N‐terminus for protease cleavage. Our observation agrees with the study of sequence conservation of ribosomal proteins that revealed that amino acids on the protein‐RNA interfaces are indeed less conserved than amino acids on the protein‐protein interfaces (Pilla & Bahadur, 2019). Supposedly, RNA‐protein interfaces are the most ancient and important for ribosome assembly, so their smaller conservation compared with protein‐protein interfaces might seem counterintuitive (Fox, 2010). However, this could be explained by the nature of protein‐RNA binding that often relies on positively charged and hydrophobic amino‐acids while the fine shape of the protein surface is not so important. On the other hand, protein‐protein interfaces strongly rely on the exact match of surface shapes which makes amino‐acid substitutions much more detrimental for the interface integrity.

Still, involvement in protein‐protein contacts does not fully explain the absence of a cleavable MTS in some less deeply buried MRPs (Fig 6C) indicating that other factors might be at play. Some possible explanations can be that a new MRP is recruited to the ribosome with its targeting signal already positioned elsewhere in its sequence. Another option is that a recruited protein has a normal cleavable MTS but after recruitment, the MTS loses its cleavage site and becomes an integral part of the structure still positioned on the ribosome surface. It is also possible that in some MRPs the flexible N‐terminal extensions play other important roles incompatible with N‐terminal targeting signal properties. In the future, more systematic analysis of mitoribosome structures from different organisms can shed light on the complex interplay between the evolution of mitoribosome assembly on the one side and the targeting signals of its components on the other.

To summarize, we suggest that at least some of the noncanonical targeting signals can develop in MRPs not only to fulfill certain functions, such as in the case of Mrp10 and Mrpl32 (Bonn *et al*, 2011; Longen *et al*, 2014), but also simply under the pressure of structural constraints imposed by mitoribosome assembly. It seems that unlike OXPHOS complexes, which also underwent complex evolution in mitochondria (Sluis *et al*, 2015), protein components of mitoribosome more often use their N‐termini to establish important interactions and thus could not have so easily developed a cleavable MTS (Fig 6C). Instead, they developed multiple internal targeting signals of different strengths such as those found in Mrp17. This might have been relatively easy for ribosomal proteins that either already contain a lot of positive charges, or can easily increase their content due to abundant protein‐RNA contacts. An alternative strategy would be to place a targeting signal on the C‐terminus. For now, the only known matrix protein with a C‐terminal targeting signal is Hmi1 that also cannot tolerate an N‐terminal extension (Lee *et al*, 1999). Such diversity of targeting signals highlights the need for better targeting signal prediction algorithms.

In this work, we have shown that many MRPs have unconventional, non‐cleavable, targeting signals that can use the same import pathway as a regular MTS. This demonstrates an incredible versatility of the mitochondrial import system that can accommodate such a range of substrates and poses the question: “what is the mechanistic basis of balancing such versatility with the specificity of protein import process?”

## Materials and Methods

### Yeast strains and plasmids

All yeast strains used in this study are listed in Appendix Table S1. Strains for fluorescent protein expression and imaging were constructed on the BY4741 haploid or BY4743 diploid background (Brachmann *et al*, 1998), except for Mrp17‐GFP expression in *∆tom20* which originated from a W303 background. For mitochondria purification, we used W303 background. Growth rescue of *∆mrp17* yeast was performed in YPH499 background using sheltered disruption—the tested *MRP17* gene version was first introduced on a plasmid (Appendix Table S2) and then genomic copy of *MRP17* was knocked out. Correct introduction of the knock out cassette instead of genomic *MRP17* was confirmed by PCR. All strains were constructed using standard LiAc/ssDNA/PEG‐based transformation protocol (Gietz & Woods, 2006). For transformation, we used the standard plasmids for PCR‐based tagging and knock outs (Longtine *et al*, 1998; Janke *et al*, 2004) and plasmids generated in this study (Appendix Table S2). Mutant versions of Mrp17 genes and genes for BRPs optimized for expression in yeast were ordered from GeneWiz or Genescript. When strains were constructed by genomic integration, primers were designed using Primers4Yeast (http://wws.weizmann.ac.il/Primers‐4‐Yeast) (Yofe and Schuldiner, 2014) or manually if the gene was truncated. Primers used in this study are listed in Appendix Table S3.

### Yeast growth

Yeast cells were grown on either liquid media or solid media that contained 2.2% agar. When only antibiotic resistance selection was used yeast cells were grown on YPD media (2% peptone, 1% yeast extract, 2% glucose) supplemented with nourseothricin (Tivan Biotech) to 0.2 g/l. For auxotrophic selections, yeast were grown in SD media (0.67% yeast nitrogen base without amino acids and with ammonium sulfate, 2% glucose, and OMM amino acid mix (Hanscho *et al*, 2012)) if necessary supplemented with the same amount of antibiotic.

### Growth assays

For growth assays with plate reader, the strains were inoculated in 96‐well plate 1 day before the experiment start if grown in fermentative media (YPD, see above) or 2 days before the experiment if grown in respiratory media YPGlycerol (2% peptone, 1% yeast extract, 2% glycerol) and incubated at 30°C with 500 rpm shaking in automated Liconic incubator. On the day of experiment, the saturated cultures were diluted 1:50 in fresh media using EVO Freedom liquid handler (Tecan) and incubated at 30°C with shaking. The optical density measurements at 600 nm were taken every 30 min with the SPARK plate reader (Tecan). Each assay was repeated two times (Appendix Fig S4).

For drop dilution assay, the yeast cultures in the phase of exponential growth (OD_600_ ~ 0.6) were sedimented and diluted in fresh YEP media to the OD = 0.1. This suspension was serially diluted 10× and 2.5 µl of each dilution was spotted on the agar plate using a multichannel pipette. The plates were incubated for 2 days at 24°, 30°, and 37°C and imaged using smartphone camera.

### Analysis of translation start site using ribosome profiling

To check for possible mis‐annotation of the translation start sites of MRPs in the *Saccharomyces* genome database, we reanalyzed data from a ribosome profiling study that was specifically designed to detect translation initiation sites (preprint: Knöringer *et al* (2021); data available at GEO with the accession number GSE172017). Briefly, ribosome profiling libraries of yeast cells (YPH499) were prepared as described (Stein *et al*, 2019) with the following modification: In one replicate, 100 µg/ml of cycloheximide was added to the yeast culture 2 min before harvesting and lysis, while in the other replicate, cells were not in contact with cycloheximide prior to cell lysis. Cycloheximide inhibits translation elongation, but not translation initiation, which results in an enrichment of ribosome footprints at translation initiation sites in CHX‐treated samples compared with untreated samples. Ribosome footprints were sequenced and aligned to the yeast genome. Footprint densities along the annotated open reading frames of MRP genes were analyzed and the translation start site was reannotated based on the following criteria: (i) the presence of contiguous ribosome footprints in all samples (previously annotated introns were taken into consideration); (ii) the presence of an enrichment of footprints in the CHX‐treated sample at the very 5′ position of the suspected ORF; (iii) the presence of an AUG codon at the suspected start site.

### Fluorescence microscopy

The day before the experiment yeast were grown to saturation. On the day of experiment, the saturated culture was diluted 1:50 in SD media without selection or with just auxotrophic selections. Cells were grown for 4 h and applied on glass‐bottom plates coated with concanavalin A and left for 20 min to adhere. If mitochondria needed to be visualized, the solution was removed from the wells and 50 nM MitoTracker Orange CMTMRos (ThermoFisher #M7510) or 500 nM MitoView 405 (Biotium #70070) diluted in imaging media (SD media with complete set of amino acids but without riboflavin) was placed in the wells for 10 min. Imaging was performed in fresh imaging media. Cells were imaged using VisiScope Confocal Cell Explorer system consisting of Olympus IX83 microscope, Zeiss Yokogawa spinning disk scanning unit equipped with PCO‐Edge sCMOS camera controlled by VisiView sofware. Images were recorded with 488 nm laser illumination for GFP channel, 561 nm laser illumination for MitoTracker Orange, 405 nm laser for MitoView 405, and 60× oil objective was used. Micrographs were cropped, and slightly adjusted for brightness and contrast using Fiji (Schindelin *et al*, 2012).

### Data analysis

The data on the first amino acid in the mitoribosome structures were extracted directly from the mmCIF files of PDB entries 6WD0 (Loveland *et al*, 2020), 5MRC (Desai *et al*, 2017), 6YWS, 6YW5 (Itoh *et al*, 2020), 6NU2 (Koripella *et al*, 2019), 6GAW (Kummer *et al*, 2018), 6XYW (Waltz *et al*, 2020), 6HIV (Ramrath *et al*, 2018), 6ZP1 (Tobiasson & Amunts, 2020) using PDBeCIF (https://pypi.org/project/PDBeCif/). The number of atoms around each Cα atom were calculated from this data using Python (McKinney, 2010).

The cleavage site in MRP N‐termini was annotated from the following sources with priority as listed: (i) original publication with N‐terminal sequencing as cited by UniProt (Graack *et al*, 1988, 1991; Grohmann *et al*, 1989, 1991; Matsushita *et al*, 1989; Dang & Ellis, 1990; Kitakawa *et al*, 1990, 1997; Boguta *et al*, 1992; Davis *et al*, 1992; Matsushita & Isono, 1993), (ii) high‐throughput N‐terminal proteomic dataset (Vögtle *et al*, 2009), (iii) cleavage site prediction by UniProt, (iv) cleavage cite prediction using MitoFates (Fukasawa *et al*, 2015). If the cleavage site annotation did not agree with the structural data (cleavage annotated after the amino acid actually present in the structure), we took the annotation from the source of next priority. If none of the annotations agreed with the structure, the N‐terminus cleavage site was marked as “NA” (Dataset EV1).

Mitochondrial targeting sequence prediction scores were calculated using MitoProt (Claros & Vincens, 1996), TargetP1 (Emanuelsson *et al*, 2000), TargetP2 (Armenteros *et al*, 2019), and MitoFates (Fukasawa *et al*, 2015) as described. The profiles and propensities of iMTSLs were calculated as described (Backes *et al*, 2018; Boos *et al*, 2018) using a web‐server (http://imlp.bio.uni‐kl.de/). Protein charge and hydrophobic moments were calculated using EMBOSS suite (Rice, 2000).

Structures were visualized using ChimeraX (Goddard *et al*, 2018), and electrostatic potentials using Chimera (Pettersen *et al*, 2004). Structural alignments were performed using FATCAT (Li *et al*, 2020), sequence alignment was performed and visualized using UniPro UGENE (Okonechnikov *et al*, 2012).

All other data analysis was performed using MATLAB (MathWorks). Plots were produced using MATLAB, Microsoft Excel, R (R Core Team, 2020), and GraphPad Prism.

### Miscellaneous

The following procedures were carried out as published before: isolation of mitochondria and *in vitro* import experiments (Peleh *et al*, 2015).

## Author contributions

YSB conceptualization, investigation, visualization, writing—original draft; TF conceptualization, investigation, visualization, writing—review and editing; FB investigation, visualization, NZ investigation; JMH conceptualization, funding acquisition, supervision, writing—original draft; MS conceptualization, funding acquisition, supervision, writing—original draft; YSB and TF contributed equally to this work.

## Conflict of interest

The authors declare that they have no conflict of interest.

## Supporting information



Appendix

Expanded View Figures PDF

Dataset EV1

Dataset EV2

Source Data for Figure 4

## Data Availability

Ribosome profiling: available at GEO, accession GSE172017 https://www.ncbi.nlm.nih.gov/geo/query/acc.cgi?acc=GSE172017
