# Supplementary material for: Widespread use of unconventional targeting signals in mitochondrial ribosome proteins
Source: EMBO J. 2021 Nov 17;41(1):e109519. doi: 10.15252/embj.2021109519 (PMC8724765; doi:10.15252/embj.2021109519)
Supplement: Supplementary file 1 — Appendix [file EMBJ-41-e109519-s001.pdf]

Appendix

Mitochondrial ribosomal proteins developed unconventional mitochondrial targeting signals due to structural constraints

Yury S. Bykov, Tamara Flohr, Felix Boos, Naama Zung, Johannes M. Herrmann, and Maya Schuldiner

Contents

Appendix Figure S1	2
Appendix Figure S2	3
Appendix Figure S3	4
Appendix Figure S4	6
Appendix Figure S5	7
Appendix Figure S6	9
Appendix Figure S7	10
Appendix Figure S8	12
Appendix Figure S9	14
Appendix Table S1	16
Appendix Table S2	19
Appendix Table S3	21

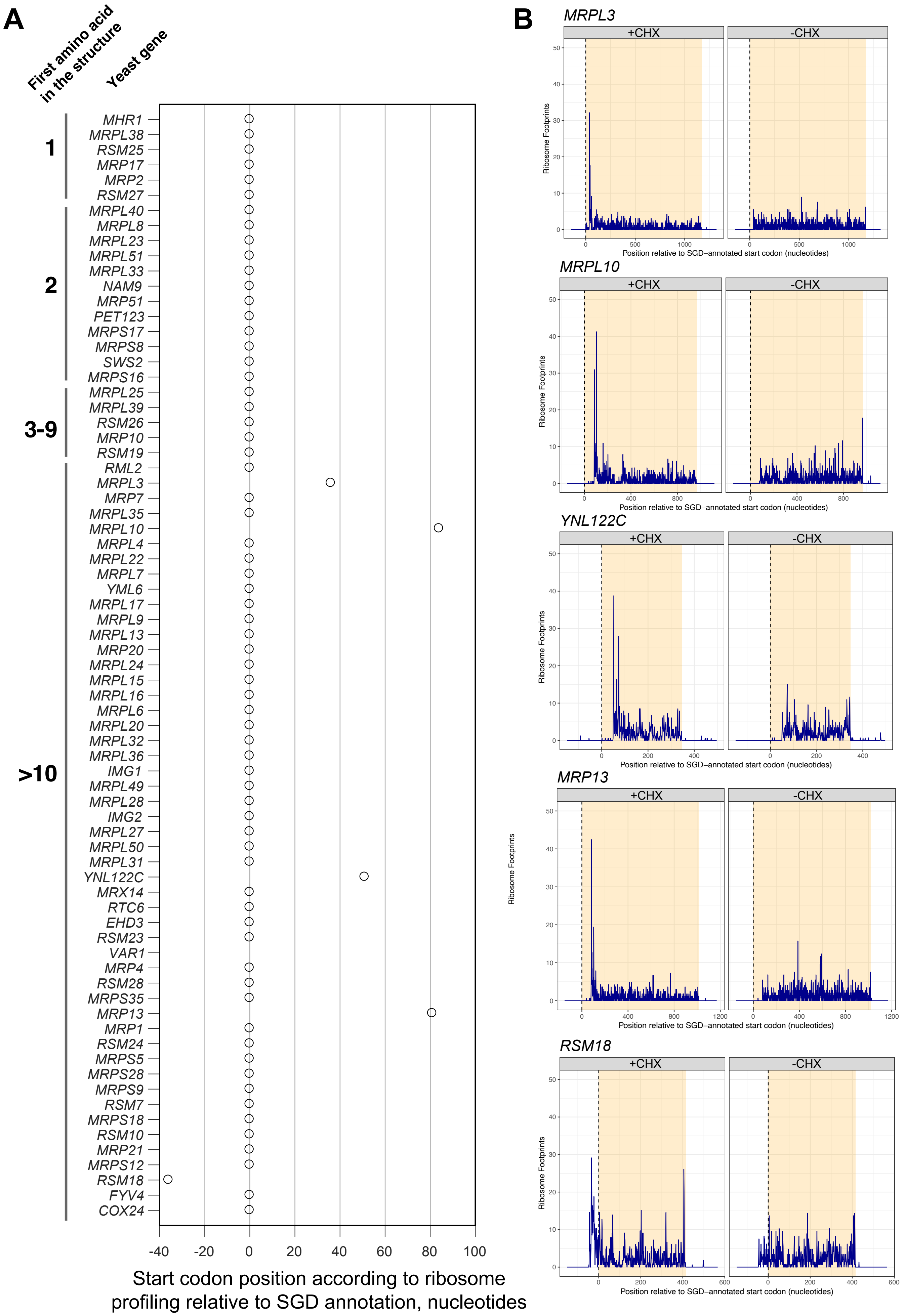

Appendix Figure S1. RiboSeq reveals no mis-annotated upstream translation starts that can account for the missing N-terminal signals. (A) For each MRP the translation start site measured by RiboSeq is plotted relative to Saccharomyces Genome Database annotation, revealing that only one protein has an N-terminal extension while 4 others have shorter N-termini, all of the misannotated proteins are anyways already reported to have an MTS (Table S1); no MRPs with their N-termini in the structure (first structured amino acid <10) had misannotated translation starts. (B) Ribosome footprints for each gene with misannotated start codons in the presence of cycloheximide (+CHX, left) and without cycloheximide (-CHX, right), plotted relative to the gene annotation in SGD (shaded in yellow).

Protein	Group	Bacteria	Fungi		Mammals		Plant	Kinetoplastida	Alveolata
		E.coli	S.cerevisiea	N.crassa	H.sapiens	S.scrofa	B.oleracea	T.brucei	T.termophyla
uL1	u/b	3	NA	59	NA	NA	NA	NA	NA
uL2	u/b	1	61	54	61	61	56	NA	1
uL3	u/b	1	21	63	45	42	64	43	32
uL4	u/b	1	29	62	45	45	89	18	22
uL5	u/b	1	19	44	NA	NA	3	NA	1
uL6	u/b	1	17	52	NA	NA	2	NA	1
bL9	u/b	1	10	52	53	53	44	9	12
uL10	u/b	1	NA	54	30	29	3	4	1
uL11	u/b	1	NA	12	18	17	15	14	1
bL12	u/b	NA	NA	NA	NA	10	NA	NA	12
uL13 (boxed)	u/b	1	2	1	2	2	15	11	28
uL14 (boxed)	u/b	1	1	1	31	31	47	NA	1
uL15	u/b	2	58	47	10	9	65	10	33
uL16	u/b	1	38	61	47	30	37	25	1
bL17	u/b	1	2	2	9	9	4	40	2
uL18	u/b	2	NA	NA	42	38	8	NA	NA
bL19	u/b	1	16	41	74	53	101	8	1
bL20	u/b	1	NA	NA	10	10	1	10	2
bL21	u/b	1	44	85	49	55	110	56	30
uL22	u/b	1	85	43	47	NA	103	2	23
uL23 (boxed)	u/b	1	55	13	2	2	4	61	6
uL24 (boxed)	u/b	1	2	1	15	11	10	1	45
bL25	u/b	1	NA	NA	NA	NA	34	NA	26
bL27	u/b	7	35	67	34	39	55	27	38
bL28	u/b	1	22	42	2	2	41	10	23
uL29	u/b	1	15	44	63	65	31	9	21
uL30	u/b	1	2	2	35	35	17	51	NA
bL31	u/b	NA	20	48	34	35	32	2	NA
bL32	u/b	1	72	64	79	79	87	26	NA
bL33	u/b	3	6	7	14	13	8	36	6
bL34	u/b	1	60	95	47	50	NA	NA	NA
bL35 (boxed)	u/b	1	54	NA	94	94	110	40	50
bL36	u/b	1	56	79	67	63	2	59	1
mL37	e	NA	NA	NA	31	30	NA	NA	NA
mL38	e	NA	20	83	27	27	NA	41	NA
mL39	e	NA	NA	NA	36	30	NA	NA	NA
mL40 (boxed)	e	NA	34	248	83	83	145	33	23
mL41	e	NA	17	9	15	14	22	47	11
mL42	e	NA	NA	NA	35	35	NA	51	NA
mL43	e	NA	2	2	2	2	4	2	2
mL44	e	NA	67	55	31	31	NA	NA	NA
mL45	e	NA	NA	NA	117	38	NA	NA	NA
mL46	e	NA	23	82	43	43	26	18	66
mL48	e	NA	NA	NA	48	48	NA	NA	NA
mL49	e	NA	41	82	38	34	NA	9	1
mL50	e	NA	38	112	56	51	NA	NA	NA
mL51	e	NA	NA	NA	32	32	NA	NA	NA
mL52	e	NA	NA	NA	24	23	NA	19	NA
mL53	e	NA	NA	1	13	2	5	37	59
mL54	e	NA	NA	77	114	71	NA	NA	40
mL57 (boxed)	e	NA	29	61	NA	NA	NA	NA	NA
mL58	e	NA	19	41	NA	NA	NA	NA	NA
mL59	e	NA	3	2	25	25	40	27	NA
mL60	e	NA	13	13	NA	NA	23	NA	NA
mL61	e	NA	NA	NA	NA	NA	NA	NA	NA
MHR1	e	NA	1	33	NA	NA	NA	NA	NA
mL62	e	NA	NA	NA	38	38	NA	NA	NA
mL63	e	NA	NA	NA	9	9	NA	17	NA
mL65	e	NA	NA	NA	41	40	NA	NA	NA
mL66	e	NA	NA	NA	35	35	NA	NA	NA

Protein	Group	Bacteria	Fungi		Mammals		Plant	Kinetoplastida	Alveolata
		E.coli	S.cerevisiea	N.crassa	H.sapiens	S.scrofa	B.oleracea	T.brucei	T.termophyla
bS1 (boxed)	u/b	NA	2	6	77	NA	NA	NA	NA
uS2	u/b	1	128	107	58	55	7	NA	1
uS3	u/b	1	30	17	36	36	3	4	1
uS4	u/b	1	2	1	NA	NA	18	NA	1
uS5	u/b	9	14	56	88	88	297	10	16
bS6 (boxed)	u/b	1	1	1	2	2	2	2	2
uS7	u/b	1	87	87	35	35	8	NA	1
uS8	u/b	1	2	2	NA	NA	2	10	NA
uS9	u/b	3	35	69	71	70	306	1	23
uS10	u/b	5	16	81	61	50	124	10	1
uS11	u/b	12	70	253	59	60	191	10	13
uS12	u/b	1	29	45	31	31	2	54	5
uS13	u/b	1	2	2	NA	NA	33	NA	3
uS14	u/b	1	1	2	28	28	69	10	1
uS15	u/b	1	34	38	66	65	316	69	18
bS16 (boxed)	u/b	1	2	2	10	NA	2	9	1
uS17	u/b	3	2	6	5	2	4	10	2
bS18 (boxed)	u/b	9	40	108	47	47	164	7	27
uS19	u/b	2	9	9	NA	NA	128	31	1
bS21	u/b	3	86	149	2	2	NA	10	1
mS22	e	NA	NA	NA	67	88	NA	10	NA
mS23 (boxed)	e	NA	1	2	2	2	6	9	1
mS25	e	NA	NA	NA	2	2	NA	NA	NA
mS26 (boxed)	e	NA	2	47	27	27	43	10	25
mS27	e	NA	42	40	36	68	NA	NA	NA
mS29	e	NA	50	57	51	45	128	9	22
mS31	e	NA	NA	NA	276	NA	NA	NA	182
mS33 (boxed)	e	NA	1	2	5	4	12	11	2
mS34	e	NA	NA	NA	6	1	50	10	10
mS35	e	NA	47	42	52	51	289	30	12
mS37	e	NA	5	2	2	2	30	20	3
mS38	e	NA	78	312	128	128	NA	141	NA
mS39	e	NA	NA	NA	56	55	NA	NA	NA
mS40	e	NA	NA	NA	52	49	NA	NA	NA
mS41	e	NA	30	43	NA	NA	41	10	24
mS42 (boxed)	e	NA	8	43	NA	NA	NA	9	NA
mS43	e	NA	18	NA	NA	NA	NA	9	NA
mS45	e	NA	27	66	NA	NA	143	NA	18
mS46	e	NA	197	223	NA	NA	NA	NA	NA
mS47	e	NA	36	42	NA	NA	42	26	24

First amino acid in the structure

- 1 to 9
- 10 to 14
- 15 and more
- Protein absent from the species or the structure

Protein origin

- u/b Universal or specific for bacterial and and mitochondrial ribosomes
- e Specific for mitochondria (any eukaryotic lineage)
- Proteins selected for experimental analysis

Appendix Figure S2. The position of the first amino acids of MRPs found in structures of different mitoribosomes are moderately conserved. The first amino acid numbers were extracted directly from the PDB entries 6WDO (*Escherichia coli*), 5MRC (*Saccharomyces cerevisiae*), 6YWS (large subunit *Neurospora crassa*), 6YW5 (small subunit *N. crassa*), 6NU2 (*Homo sapiens*), 6GAW (*Sus scrofa*), 6XYW (*Brassica oleracea*), 6HIV (*Trypanosoma brucei*), 6ZP1 (*Tetrahymena thermophila*). Only MRPs occurring in two or more structures are shown. MRPs selected for further investigation are highlighted with red rectangles.

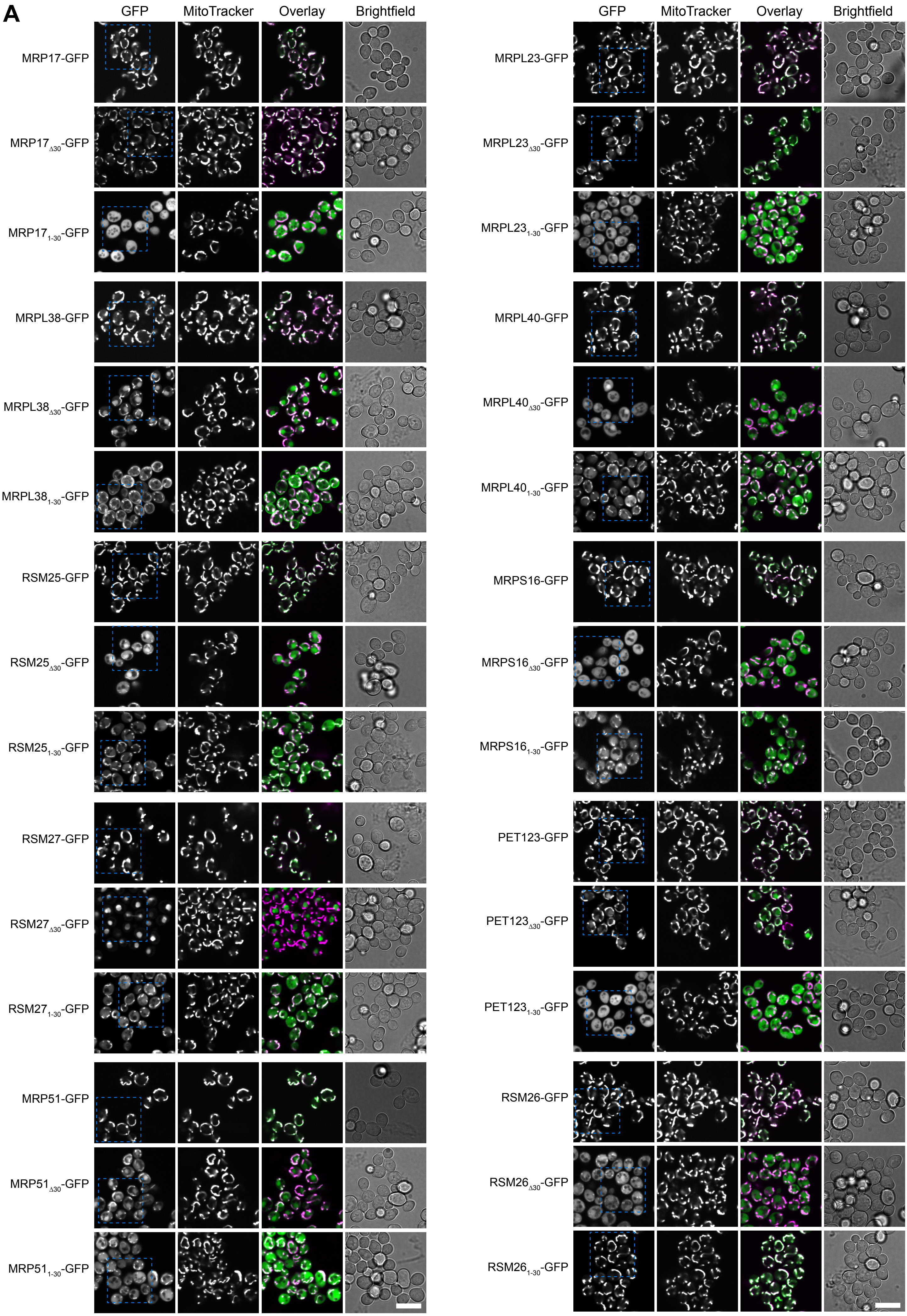

Appendix Figure S3 - continues on the next page

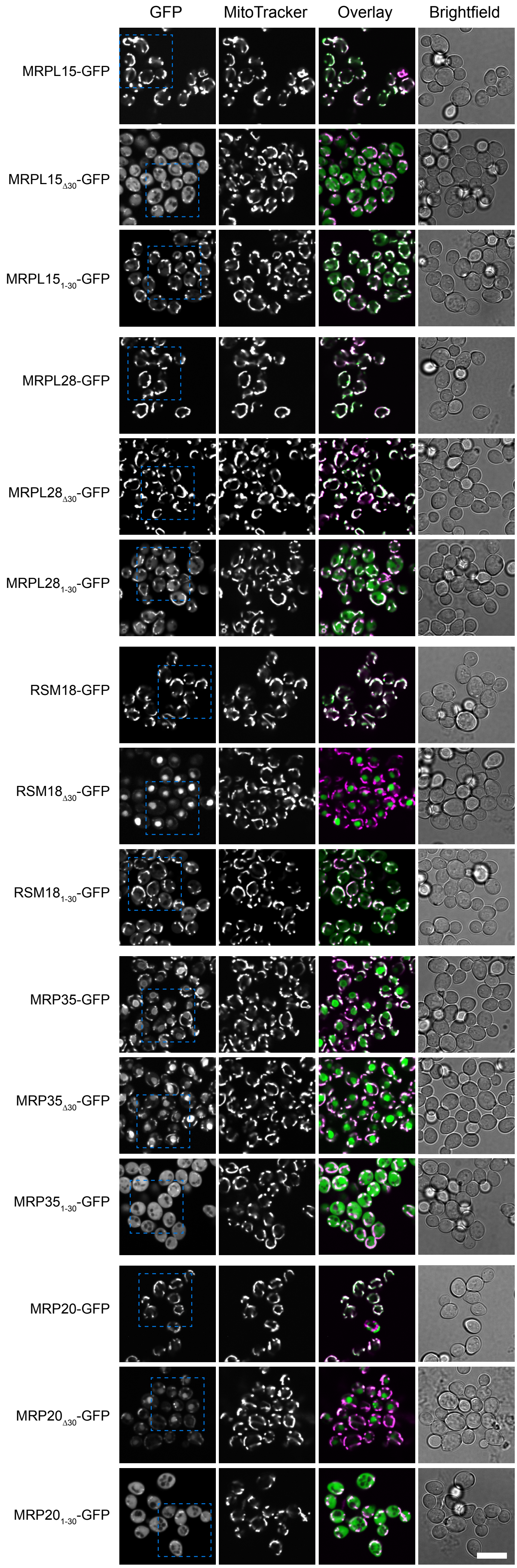

Appendix Figure S3. MRPs have N-termini with various targeting properties. Full set of micrographs in all channels for yeast strains shown in Fig. 1C-E. The regions identical to panels in Fig. 1C-E are shown with dashed blue line (rotated 90˚ relative to Fig. 1).

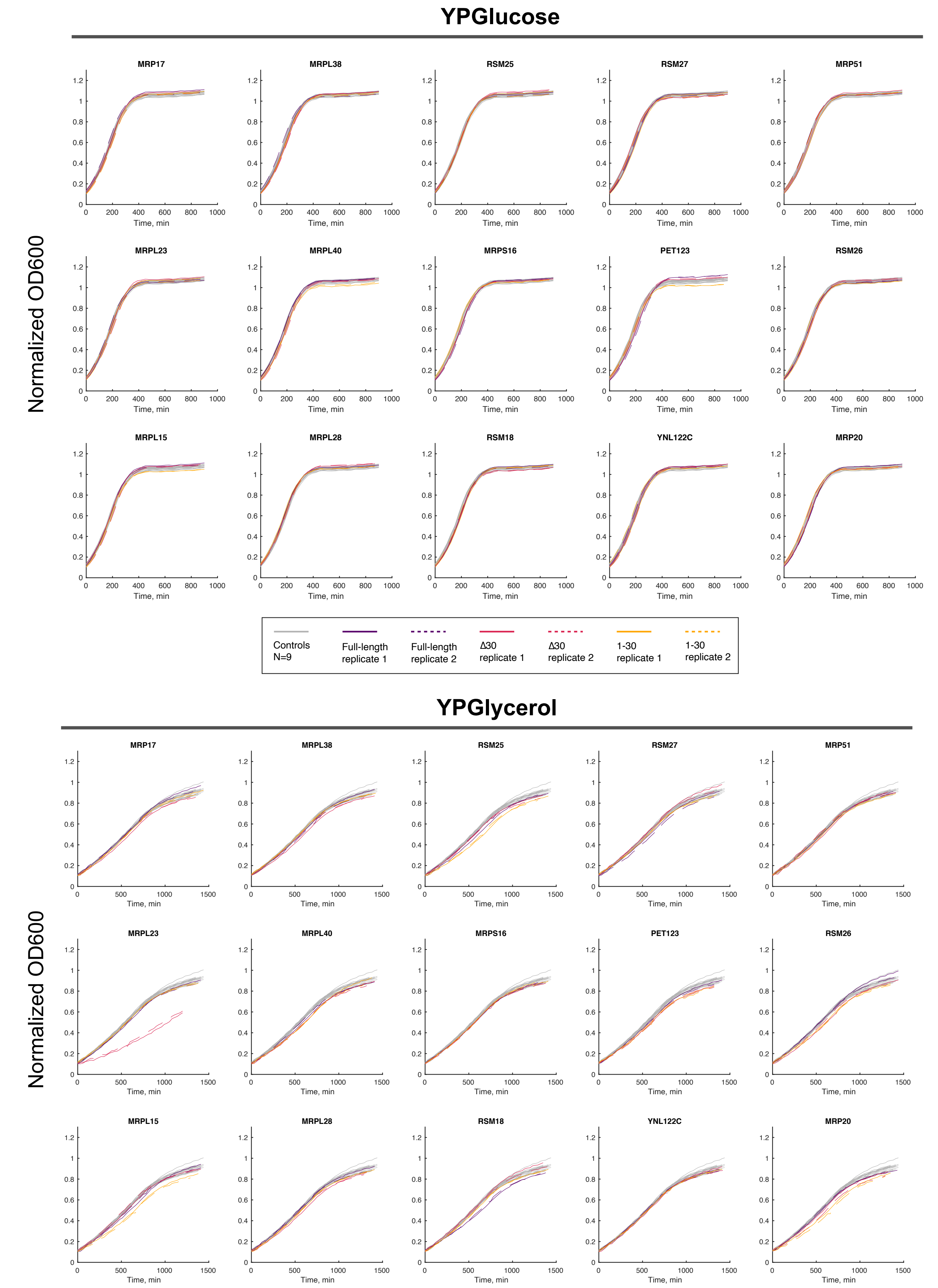

Appendix Figure S4. MRP truncations in the genomic locus of one allele in diploid yeast strains have no dominant negative growth defects in fermentative media and rare defects in respiratory media despite having non-mitochondrial localizations (see Figure 1). Growth curves in fermentative (YPDextrose) and respiratory media (YPGlycerol) for yeast expressing each of the MRP truncations (in 2 replicates) are plotted besides WT controls not expressing any construct (grey lines); Curves are normalized by subtracting the minimal value of each to compensate for the background Optical Density (OD) and then by plotting all curves only from the timepoint when the normalized OD reached 0.1 to compensate for different lag-times before the exponential phase; thus only the amplitudes and slopes of the growth curves can be compared but not the lag-times which are mostly attributed to inaccuracies of preparing the starting culture dilution.

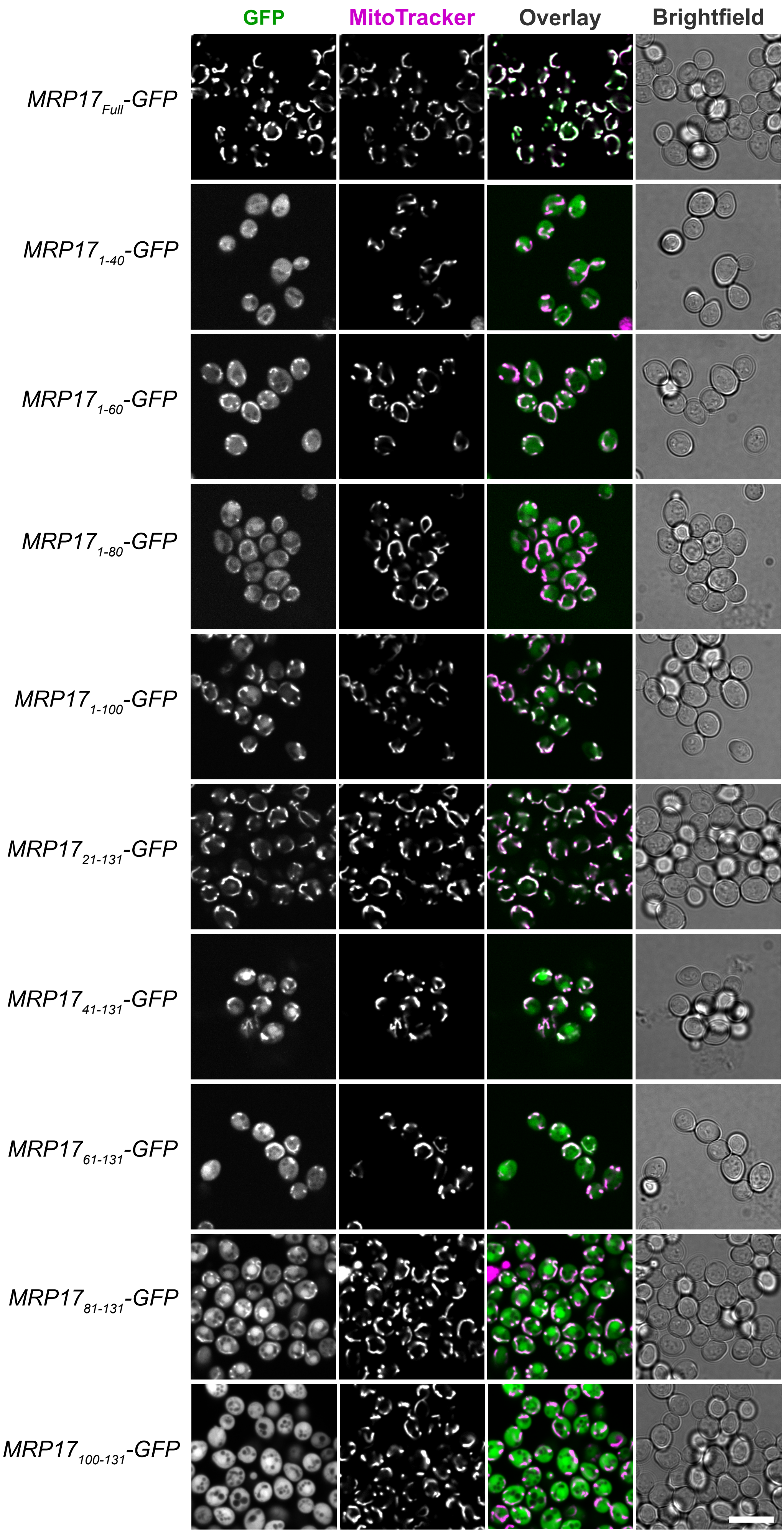

Appendix Figure S5 - continues on the next page.

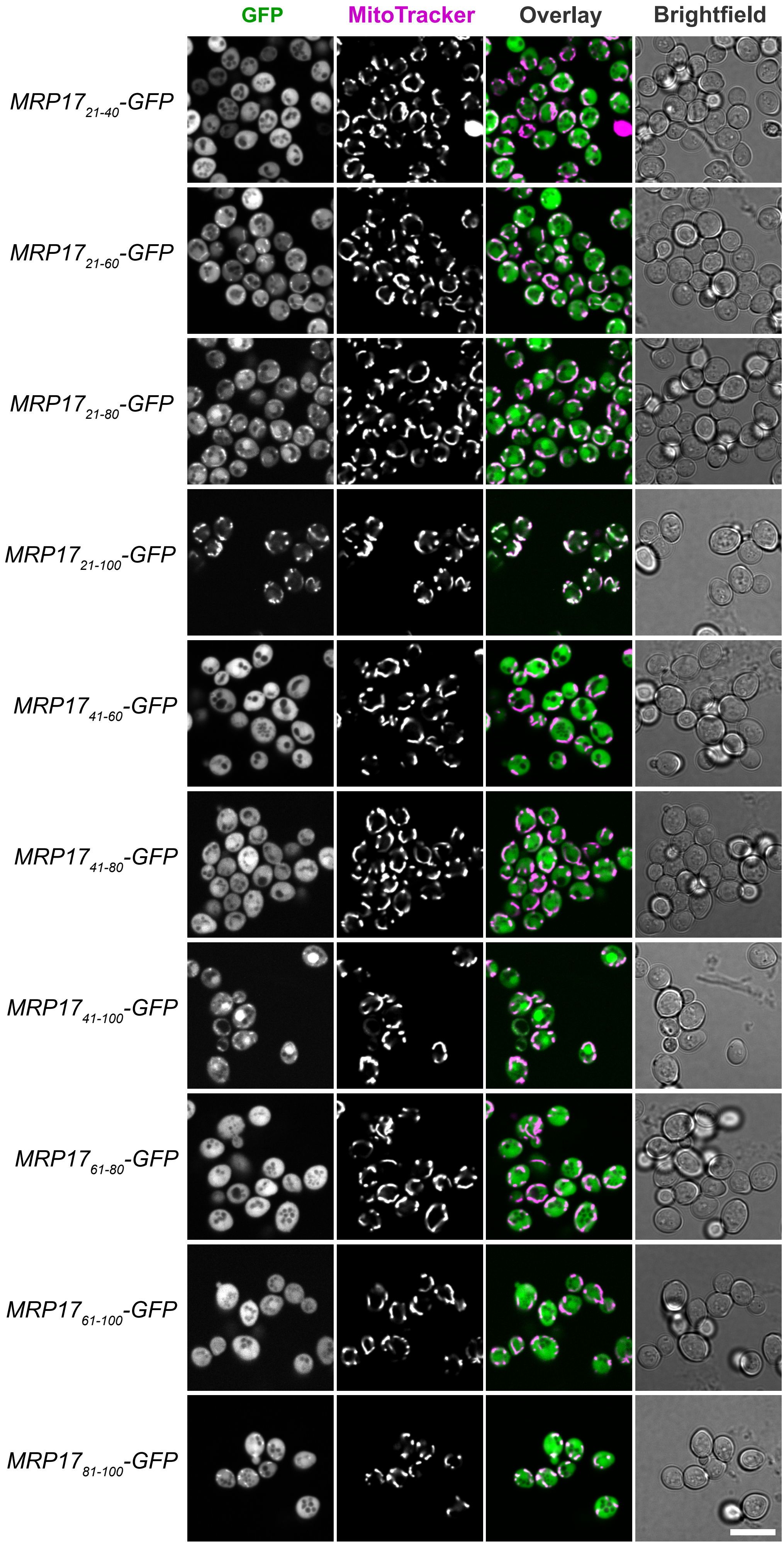

Appendix Figure S5. The ability of different parts of the Mrp17 sequence to support targeting of GFP to mitochondria. Full sets of micrographs in different fluorescent channels for all the studied Mrp17 truncations. Micrographs for truncations $MRP17_{21\text{-}100}$-GFP, $Mrp17_{21\text{-}60}$-GFP, and $Mrp17_{61\text{-}100}$-GFP are the same as shown in Fig. 2, phenotype interpretations are summarized in Fig. EV4. Scale bar 10 μm.

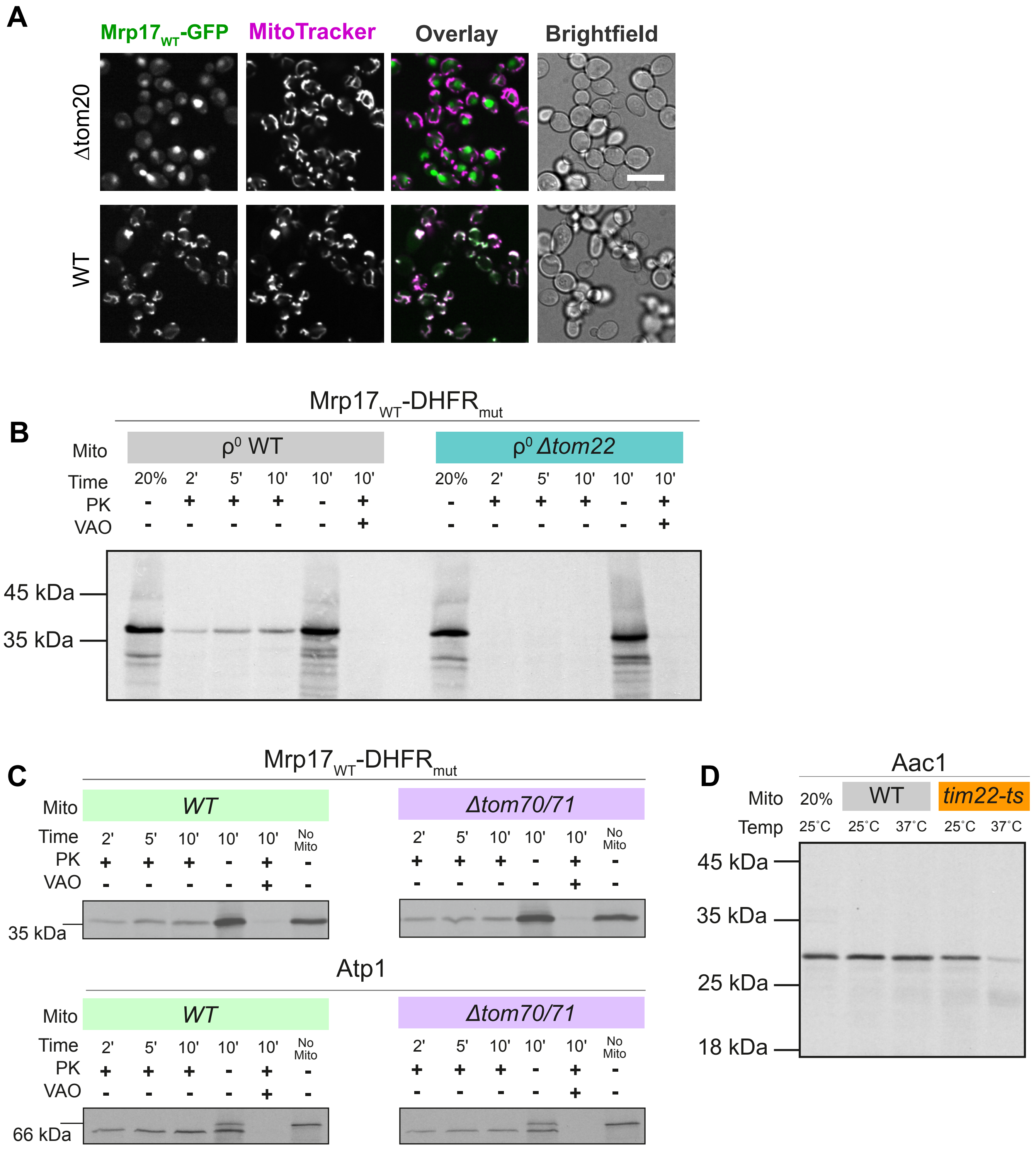

Appendix Figure S6. The translocation pathway of Mrp17 is similar to MTS-containing proteins. (A) Expression of Mrp17-GFP in WT and *Δtom20* yeast cells visualized by fluorescent microscopy. The micrograph for *Δtom20* strain is the same as shown in Fig. 4D. Scale bar is 10 μm. (B) Full autoradiograph for *in vitro* translocation of Mrp17-DHFR$_{mut}$ into mitochondria with WT Tom22 and mitochondria purified from *Δtom22* yeast, same as shown cropped in Fig. 4E, import was performed as described in Fig. 2 legend. (C) *In vitro* translocation of Mrp17-DHFR$_{mut}$ and control protein Atp1 into mitochondria isolated from WT and a *Δtom70/71* mutant showing no Tom70/71-dependence for Mrp17 translocation. (D) Full autoradiograph for Aac1 import into WT and *tim22-ts* mitochondria for Fig. 4H. All *in vitro* import experiments were performed as described in the legend for Fig.2 (panels B,C) or Fig. 4 (panel D).

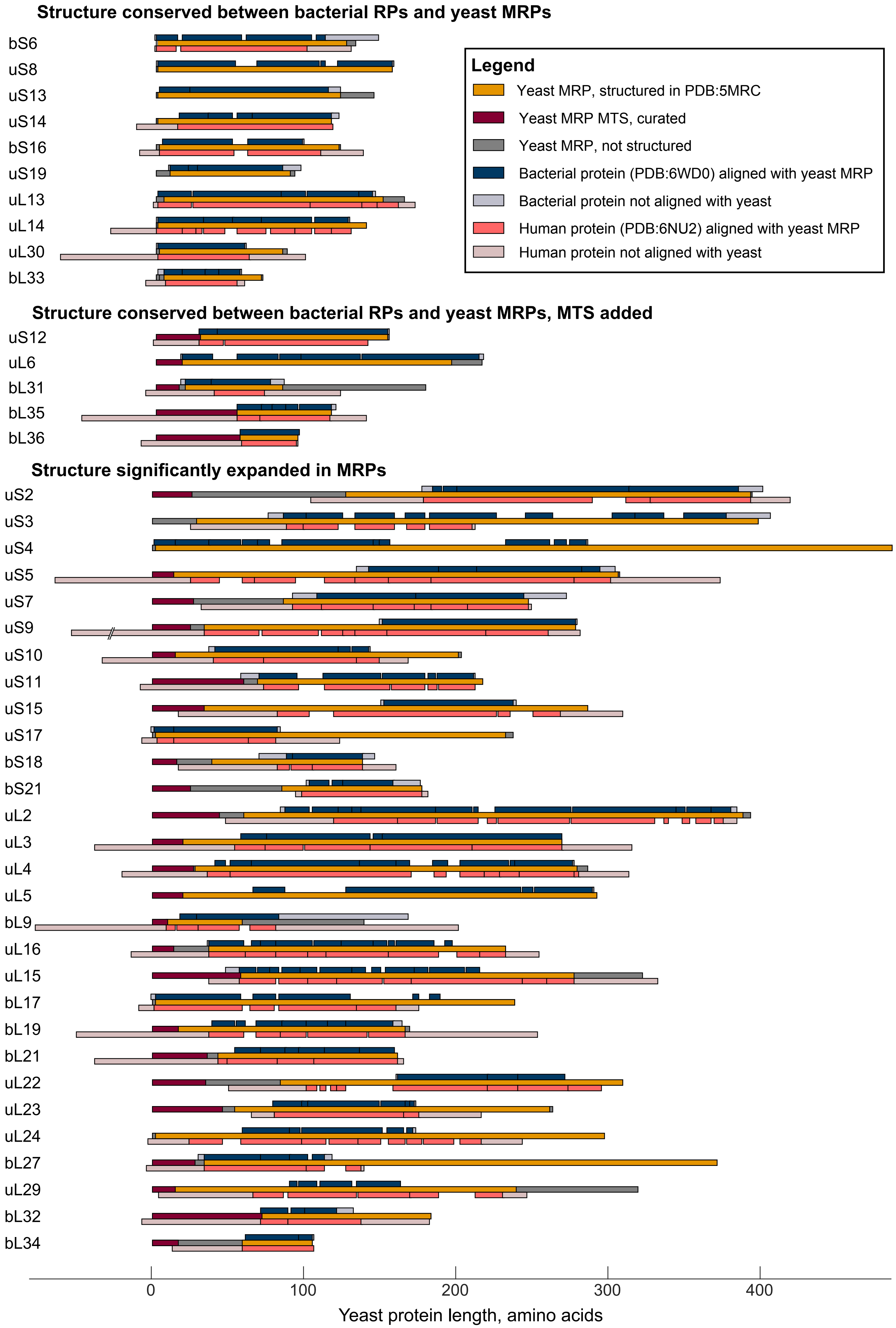

Appendix Figure S7 - legend appears on the next next page.

Appendix Figure S7. Structural alignments of yeast and human MRPs and their bacterial homologs. The structures of yeast MRPs that have bacterial homologs were aligned to their bacterial and human (when present) homologs using flexible structural alignment tool FATCAT (Li *et al.*, 2020), and the resulting alignment was plotted relative to the length of yeast proteins, deletions in the yeast proteins relative to human and bacterial homologs are not plotted and the positions of corresponding insertions within human and bacterial sequences are indicated as solid black lines. MTS of yeast proteins were annotated according to Dataset EV1. MRPs are grouped by structure conservation relative to bacterial homologs and then sorted by name.

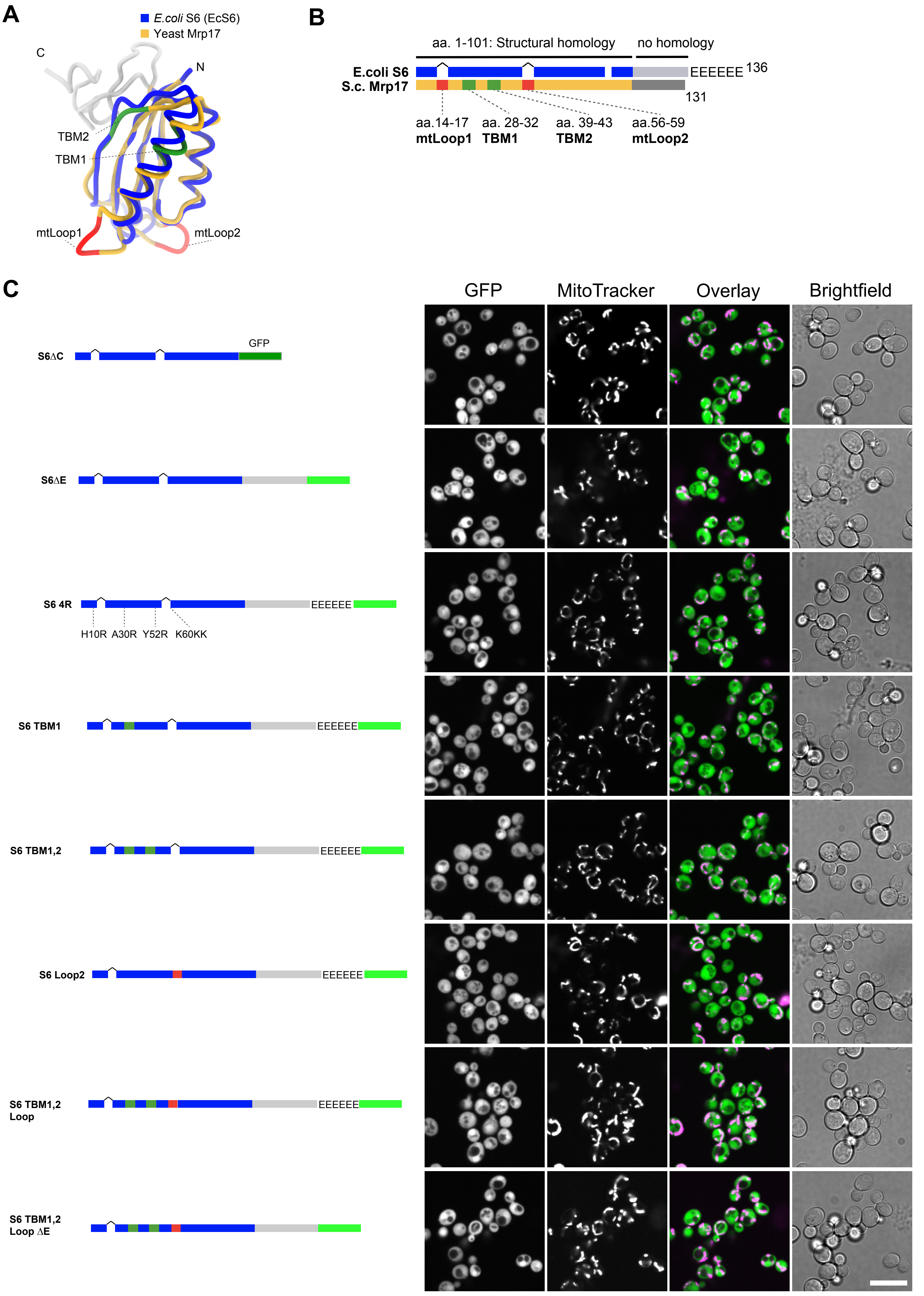

Appendix Figure S8 - continues on the next page.

Appendix Figure S8. Comparison of Mrp17 and its bacterial homolog. (A) Structural alignment of the yeast Mrp17 (from PDB:5MRC) and its E. coli homolog ribosomal protein S6 (from PDB: 6WD0) viewed from its cytosol-facing side, highlighting regions of structural homology (blue and yellow) and non-homologous C-terminal regions (grey), Tom20-binding domains in Mrp17 are highlighted in green, additional loops inserted in Mrp17 are shown in red, schematic summaries of such structural alignments for all MRPs and their bacterial homologs are shown in Appendix Fig. S7. (B) highlighting additional features of Mrp17 structure (two loops and two Tom20-binding motifs) and six glutamate residues (E's) at the C-terminus of bacterial S6. (C) Schematic representations of chimeric constructs incorporating Mrp17 features into EcS6 (corresponds to schematic in panel B) and fluorescence micrographs of yeast expressing each respective construct. Scale bar in all panels is 10 μm.

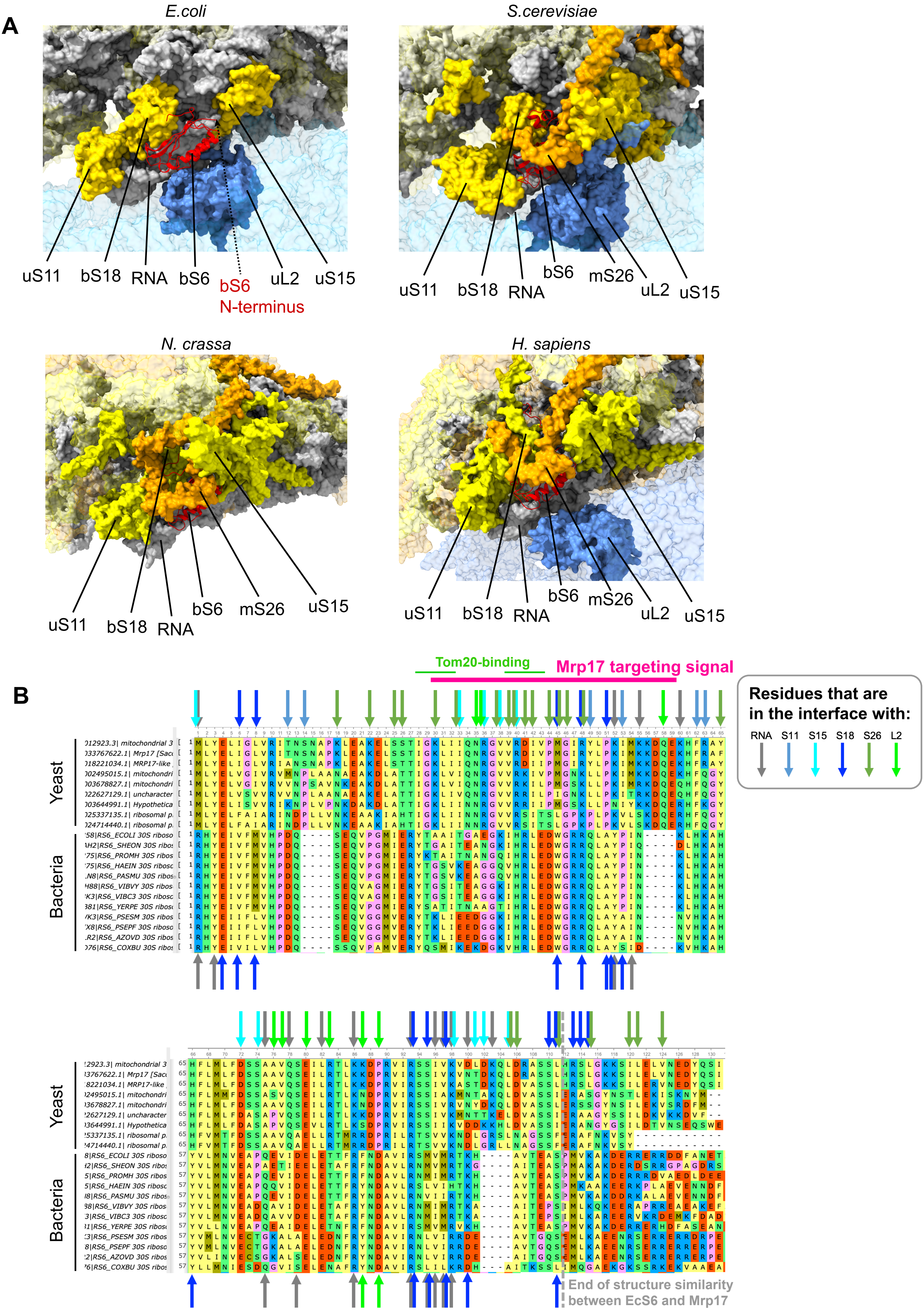

Appendix Figure S9 - legend appears on the next next page.

Appendix Figure S9. Mrp17 and EcS6 in the context of ribosome structures. A) A comparison of the structural environment of the S6 protein in bacterial ribosome (PDB:6WD0, top left), yeast mitoribosome (PDB:5MRC, top right), *N. crassa* mitoribosome (small subunit PDB:6YW5, bottom left), and human mitoribosome (PDB:5NU2, bottom right). Highlighted are the proteins interacting with S6. RNA is shown in grey, small subunit (SSU) proteins in yellow (highlighted are solid and others are transparent), S6 is red, all large subunit (LSU) components are in blue (highlighted are solid and others are transparent), mitochondria-specific protein mS26 is in orange. (B) Structural alignment of Mrp17 (first sequence) and its yeast homologs with EcS6 (first out of bacterial) and its bacterial homologs highlighting involvement of each amino acid in protein-protein and protein-RNA interfaces (arrows, see legend on the figure) in yeast mitoribosome (on top) and bacterial ribosome (in the bottom), amino acids are numbered relative to Mrp17, thus the N-terminal Met in bacterial proteins is not shown.

Appendix Table S1. A list of yeast strains used in this study.

Strain number	Name	Genotype	Comments	Mating type
HHY0003	YPH499	*ura3-52 lys2-801_amber ade2-101_ochre trp1-Δ63 his3-*		a
HHY1104	Δtom70/71	*leu2-3,-112 his3-11,-15 trp1-1 ura3-1 ade2-1 can1-100 tom70Δ::KANMX4, tom71Δ::NATNT2*	Jores et al. 2018	Alpha
HHY1105	W303	*leu2-3,-112 his3-11,-15 trp1-1 ura3-1 ade2-1 can1-100*		a
HHY1819	Δtom20	*leu2-3,-112 his3-11,-15 trp1-1 ura3-1 ade2-1 can1-100*	Müller et al. 2011	a
HHY1854	Δmrp17 - MRP17(K-R)	*YPH499 MRP17::NAT, pYX232-MRP17(K-R)*		a
HHY3181	YPH499 rho0	*YPH499 rho0*	Based on YPH499, control for Δto	a
HHY3182	Δtom22	*YPH499 rho0 TOM22::HIS*	Becker et al. 2011	a
HHY3199	YPH499 - pYX232 ev	YPH499		a
HHY3200	Δmrp17 – pYX232 ev	YPH499, MRP17::NAT		a
HHY3201	Δmrp17 – Mrp17	YPH499, pYX232-Mrp17, MRP17::NAT		a
HHY3202	Δmrp17 – Su9-Mrp17	YPH499, pYX232 - Su9-Mrp17, MRP17::NAT		a
HHY3203	Δmrp17 – Mrp17(K-A)	YPH499, pYX232 - Mrp17(K-A) MRP17::NAT		a
HHY3204	Δmrp17 – Su9-Mrp17(K-A)	YPH499, pYX232 - Su9-Mrp17(K-A), MRP17::NAT		a
YMS0701	BY4741	*his3Δ1 leu2Δ0 met15Δ0 ura3Δ0*	Brachmann et al. 1998	a
YMS0721	YMS721	*his3Δ1 leu2Δ0 met15Δ0 ura3Δ0 can1Δ::STE2pr-sp HIS5 lyp1Δ::STE3pr-LEU2*	Cohen and Schuldiner 2011	Alpha
YMS123	BY4743	*his3Δ1/his3Δ1 leu2Δ0/leu2Δ0 lys2Δ0/+ met15dΔ0/+ ura3Δ0/ura3Δ0 NAT-GPDpr-MRP17-GFP-HIS*	Brachmann et al. 1998	diploid
YMS4317	GPDp:MRP17(full)-GFP	*his3Δ1/his3Δ1 leu2Δ0/leu2Δ0 met15Δ0/met15Δ0 ura3Δ0/ura3Δ0 NAT:GPDpr:MRP17:GFP:His5/MRP17*	Based on BY4743	diploid
YMS4319	GPDp:MRP17(1-40)-GFP	*his3Δ1/his3Δ1 leu2Δ0/leu2Δ0 met15Δ0/met15Δ0 ura3Δ0/ura3Δ0 NAT:GPDpr:MRP17(1-40):GFP:His5/MRP17*	Based on BY4743	diploid
YMS4320	GPDp:MRP17(1-60)-GFP	*his3Δ1/his3Δ1 leu2Δ0/leu2Δ0 met15Δ0/met15Δ0 ura3Δ0/ura3Δ0 NAT:GPDpr:MRP17(1-60):GFP:His5/MRP17*	Based on BY4743	diploid
YMS4321	GPDp:MRP17(1-80)-GFP	*his3Δ1/his3Δ1 leu2Δ0/leu2Δ0 met15Δ0/met15Δ0 ura3Δ0/ura3Δ0 NAT:GPDpr:MRP17(1-80):GFP:His5/MRP17*	Based on BY4743	diploid
YMS4322	GPDp:MRP17(1-100)-GFP	*his3Δ1/his3Δ1 leu2Δ0/leu2Δ0 met15Δ0/met15Δ0 ura3Δ0/ura3Δ0 NAT:GPDpr:MRP17(1-100):GFP:His5/MRP17*	Based on BY4743	diploid
YMS5662	TPIpr-EcS6-GFP	*his3Δ1 leu2Δ0 met15Δ0 ura3Δ0 ho::TPIpr-EcS6-GFP-NAT*	Based on BY4741	a
YMS5663	TPIpr-EcL14-GFP	*his3Δ1 leu2Δ0 met15Δ0 ura3Δ0 ho::TPIpr-EcL14-GFP-NAT*	Based on BY4741	a
YMS5664	TPIpr-EcS16-GFP	*his3Δ1 leu2Δ0 met15Δ0 ura3Δ0 ho::TPIpr-EcS16-GFP-NAT*	Based on BY4741	a
YMS5665	TPIpr-EcL13-GFP	*his3Δ1 leu2Δ0 met15Δ0 ura3Δ0 ho::TPIpr-EcL13-GFP-NAT*	Based on BY4741	a
YMS5671	GPDpr-MRP17-GFP	*his3Δ1/his3Δ1 leu2Δ0/leu2Δ0 lys2Δ0/+ met15dΔ0/+ ura3Δ0/ura3Δ0 NAT-GPDpr-MRP17-GFP-HIS*	Based on BY4743	diploid
YMS5672	GPDpr-MRPL38-GFP	*his3Δ1/his3Δ1 leu2Δ0/leu2Δ0 lys2Δ0/+ met15dΔ0/+ ura3Δ0/ura3Δ0 NAT-GPDpr-MRPL38-GFP-HIS*	Based on BY4743	diploid
YMS5673	GPDpr-RSM25-GFP	*his3Δ1/his3Δ1 leu2Δ0/leu2Δ0 lys2Δ0/+ met15dΔ0/+ ura3Δ0/ura3Δ0 NAT-GPDpr-RSM25-GFP-HIS*	Based on BY4743	diploid
YMS5674	GPDpr-RSM27-GFP	*his3Δ1/his3Δ1 leu2Δ0/leu2Δ0 lys2Δ0/+ met15dΔ0/+ ura3Δ0/ura3Δ0 NAT-GPDpr-RSM27-GFP-HIS*	Based on BY4743	diploid
YMS5675	GPDpr-MRP51-GFP	*his3Δ1/his3Δ1 leu2Δ0/leu2Δ0 lys2Δ0/+ met15dΔ0/+ ura3Δ0/ura3Δ0 NAT-GPDpr-MRP51-GFP-HIS*	Based on BY4743	diploid
YMS5676	GPDpr-MRPL23-GFP	*his3Δ1/his3Δ1 leu2Δ0/leu2Δ0 lys2Δ0/+ met15dΔ0/+ ura3Δ0/ura3Δ0 NAT-GPDpr-MRPL23-GFP-HIS*	Based on BY4743	diploid
YMS5677	GPDpr-MRPL40-GFP	*his3Δ1/his3Δ1 leu2Δ0/leu2Δ0 lys2Δ0/+ met15dΔ0/+ ura3Δ0/ura3Δ0 NAT-GPDpr-MRPL40-GFP-HIS*	Based on BY4743	diploid
YMS5678	GPDpr-MRPS16-GFP	*his3Δ1/his3Δ1 leu2Δ0/leu2Δ0 lys2Δ0/+ met15dΔ0/+ ura3Δ0/ura3Δ0 NAT-GPDpr-MRPS16-GFP-HIS*	Based on BY4743	diploid
YMS5679	GPDpr-PET123-GFP	*his3Δ1/his3Δ1 leu2Δ0/leu2Δ0 lys2Δ0/+ met15dΔ0/+ ura3Δ0/ura3Δ0 NAT-GPDpr-PET123-GFP-HIS*	Based on BY4743	diploid
YMS5680	GPDpr-RSM26-GFP	*his3Δ1/his3Δ1 leu2Δ0/leu2Δ0 lys2Δ0/+ met15dΔ0/+ ura3Δ0/ura3Δ0 NAT-GPDpr-RSM26-GFP-HIS*	Based on BY4743	diploid
YMS5681	GPDpr-MRPL15-GFP	*his3Δ1/his3Δ1 leu2Δ0/leu2Δ0 lys2Δ0/+ met15dΔ0/+ ura3Δ0/ura3Δ0 NAT-GPDpr-MRPL15-GFP-HIS*	Based on BY4743	diploid
YMS5682	GPDpr-MRPL28-GFP	*his3Δ1/his3Δ1 leu2Δ0/leu2Δ0 lys2Δ0/+ met15dΔ0/+ ura3Δ0/ura3Δ0 NAT-GPDpr-MRPL28-GFP-HIS*	Based on BY4743	diploid
YMS5683	GPDpr-RSM18-GFP	*his3Δ1/his3Δ1 leu2Δ0/leu2Δ0 lys2Δ0/+ met15dΔ0/+ ura3Δ0/ura3Δ0 NAT-GPDpr-RSM18-GFP-HIS*	Based on BY4743	diploid
YMS5684	GPDpr-YNL122C-GFP	*his3Δ1/his3Δ1 leu2Δ0/leu2Δ0 lys2Δ0/+ met15dΔ0/+ ura3Δ0/ura3Δ0 NAT-GPDpr-YNL122C-GFP-HIS*	Based on BY4743	diploid
YMS5685	GPDpr-MRP20-GFP	*his3Δ1/his3Δ1 leu2Δ0/leu2Δ0 lys2Δ0/+ met15dΔ0/+ ura3Δ0/ura3Δ0 NAT-GPDpr-MRP20-GFP-HIS*	Based on BY4743	diploid
YMS5686	GPDpr-MRP17Δ30-GFP	*his3Δ1/his3Δ1 leu2Δ0/leu2Δ0 lys2Δ0/+ met15dΔ0/+ ura3Δ0/ura3Δ0 NAT-GPDpr-MRP17Δ30-GFP-HIS*	Based on BY4743	diploid
YMS5687	GPDpr-MRPL38Δ30-GFP	*his3Δ1/his3Δ1 leu2Δ0/leu2Δ0 lys2Δ0/+ met15dΔ0/+ ura3Δ0/ura3Δ0 NAT-GPDpr-MRPL38Δ30-GFP-HIS*	Based on BY4743	diploid
YMS5688	GPDpr-RSM25Δ30-GFP	*his3Δ1/his3Δ1 leu2Δ0/leu2Δ0 lys2Δ0/+ met15dΔ0/+ ura3Δ0/ura3Δ0 NAT-GPDpr-RSM25Δ30-GFP-HIS*	Based on BY4743	diploid
YMS5689	GPDpr-RSM27Δ30-GFP	*his3Δ1/his3Δ1 leu2Δ0/leu2Δ0 lys2Δ0/+ met15dΔ0/+ ura3Δ0/ura3Δ0 NAT-GPDpr-RSM27Δ30-GFP-HIS*	Based on BY4743	diploid
YMS5690	GPDpr-MRP51Δ30-GFP	*his3Δ1/his3Δ1 leu2Δ0/leu2Δ0 lys2Δ0/+ met15dΔ0/+ ura3Δ0/ura3Δ0 NAT-GPDpr-MRP51Δ30-GFP-HIS*	Based on BY4743	diploid

YMS5691	GPDpr-MRPL23Δ30-GFP	*his3Δ1/his3Δ1 leu2Δ0/leu2Δ0 lys2Δ0/+ met15dΔ0/+ ura3Δ0/ura3Δ0 NAT-GPDpr-MRPL23Δ30-GFP-HIS*	Based on BY4743	diploid
YMS5692	GPDpr-MRPL40Δ30-GFP	*his3Δ1/his3Δ1 leu2Δ0/leu2Δ0 lys2Δ0/+ met15dΔ0/+ ura3Δ0/ura3Δ0 NAT-GPDpr-MRPL40Δ30-GFP-HIS*	Based on BY4743	diploid
YMS5693	GPDpr-MRPS16Δ30-GFP	*his3Δ1/his3Δ1 leu2Δ0/leu2Δ0 lys2Δ0/+ met15dΔ0/+ ura3Δ0/ura3Δ0 NAT-GPDpr-MRPS16Δ30-GFP-HIS*	Based on BY4743	diploid
YMS5694	GPDpr-PET123Δ30-GFP	*his3Δ1/his3Δ1 leu2Δ0/leu2Δ0 lys2Δ0/+ met15dΔ0/+ ura3Δ0/ura3Δ0 NAT-GPDpr-PET123Δ30-GFP-HIS*	Based on BY4743	diploid
YMS5695	GPDpr-RSM26Δ30-GFP	*his3Δ1/his3Δ1 leu2Δ0/leu2Δ0 lys2Δ0/+ met15dΔ0/+ ura3Δ0/ura3Δ0 NAT-GPDpr-RSM26Δ30-GFP-HIS*	Based on BY4743	diploid
YMS5696	GPDpr-MRPL15Δ30-GFP	*his3Δ1/his3Δ1 leu2Δ0/leu2Δ0 lys2Δ0/+ met15dΔ0/+ ura3Δ0/ura3Δ0 NAT-GPDpr-MRPL15Δ30-GFP-HIS*	Based on BY4743	diploid
YMS5697	GPDpr-MRPL28Δ30-GFP	*his3Δ1/his3Δ1 leu2Δ0/leu2Δ0 lys2Δ0/+ met15dΔ0/+ ura3Δ0/ura3Δ0 NAT-GPDpr-MRPL28Δ30-GFP-HIS*	Based on BY4743	diploid
YMS5698	GPDpr-RSM18Δ30-GFP	*his3Δ1/his3Δ1 leu2Δ0/leu2Δ0 lys2Δ0/+ met15dΔ0/+ ura3Δ0/ura3Δ0 NAT-GPDpr-RSM18Δ30-GFP-HIS*	Based on BY4743	diploid
YMS5699	GPDpr-YNL122CΔ30-GFP	*his3Δ1/his3Δ1 leu2Δ0/leu2Δ0 lys2Δ0/+ met15dΔ0/+ ura3Δ0/ura3Δ0 NAT-GPDpr-YNL122CΔ30-GFP-HIS*	Based on BY4743	diploid
YMS5700	GPDpr-MRP20Δ30-GFP	*his3Δ1/his3Δ1 leu2Δ0/leu2Δ0 lys2Δ0/+ met15dΔ0/+ ura3Δ0/ura3Δ0 NAT-GPDpr-MRP20Δ30-GFP-HIS*	Based on BY4743	diploid
YMS5717	GPDpr-MRP17(1-30)-GFP	*his3Δ1/his3Δ1 leu2Δ0/leu2Δ0 lys2Δ0/+ met15dΔ0/+ ura3Δ0/ura3Δ0 NAT-GPDpr-MRP17(1-30)-GFP-HIS*	Based on BY4743	diploid
YMS5718	GPDpr-MRPL38(1-30)-GFP	*his3Δ1/his3Δ1 leu2Δ0/leu2Δ0 lys2Δ0/+ met15dΔ0/+ ura3Δ0/ura3Δ0 NAT-GPDpr-MRPL38(1-30)-GFP-HIS*	Based on BY4743	diploid
YMS5719	GPDpr-RSM25(1-30)-GFP	*his3Δ1/his3Δ1 leu2Δ0/leu2Δ0 lys2Δ0/+ met15dΔ0/+ ura3Δ0/ura3Δ0 NAT-GPDpr-RSM25(1-30)-GFP-HIS*	Based on BY4743	diploid
YMS5720	GPDpr-RSM27(1-30)-GFP	*his3Δ1/his3Δ1 leu2Δ0/leu2Δ0 lys2Δ0/+ met15dΔ0/+ ura3Δ0/ura3Δ0 NAT-GPDpr-RSM27(1-30)-GFP-HIS*	Based on BY4743	diploid
YMS5721	GPDpr-MRP51(1-30)-GFP	*his3Δ1/his3Δ1 leu2Δ0/leu2Δ0 lys2Δ0/+ met15dΔ0/+ ura3Δ0/ura3Δ0 NAT-GPDpr-MRP51(1-30)-GFP-HIS*	Based on BY4743	diploid
YMS5722	GPDpr-MRPL23(1-30)-GFP	*his3Δ1/his3Δ1 leu2Δ0/leu2Δ0 lys2Δ0/+ met15dΔ0/+ ura3Δ0/ura3Δ0 NAT-GPDpr-MRPL23(1-30)-GFP-HIS*	Based on BY4743	diploid
YMS5723	GPDpr-MRPL40(1-30)-GFP	*his3Δ1/his3Δ1 leu2Δ0/leu2Δ0 lys2Δ0/+ met15dΔ0/+ ura3Δ0/ura3Δ0 NAT-GPDpr-MRPL40(1-30)-GFP-HIS*	Based on BY4743	diploid
YMS5724	GPDpr-MRPS16(1-30)-GFP	*his3Δ1/his3Δ1 leu2Δ0/leu2Δ0 lys2Δ0/+ met15dΔ0/+ ura3Δ0/ura3Δ0 NAT-GPDpr-MRPS16(1-30)-GFP-HIS*	Based on BY4743	diploid
YMS5725	GPDpr-PET123(1-30)-GFP	*his3Δ1/his3Δ1 leu2Δ0/leu2Δ0 lys2Δ0/+ met15dΔ0/+ ura3Δ0/ura3Δ0 NAT-GPDpr-PET123(1-30)-GFP-HIS*	Based on BY4743	diploid
YMS5726	GPDpr-RSM26(1-30)-GFP	*his3Δ1/his3Δ1 leu2Δ0/leu2Δ0 lys2Δ0/+ met15dΔ0/+ ura3Δ0/ura3Δ0 NAT-GPDpr-RSM26(1-30)-GFP-HIS*	Based on BY4743	diploid
YMS5727	GPDpr-MRPL15(1-30)-GFP	*his3Δ1/his3Δ1 leu2Δ0/leu2Δ0 lys2Δ0/+ met15dΔ0/+ ura3Δ0/ura3Δ0 NAT-GPDpr-MRPL15(1-30)-GFP-HIS*	Based on BY4743	diploid
YMS5728	GPDpr-MRPL28(1-30)-GFP	*his3Δ1/his3Δ1 leu2Δ0/leu2Δ0 lys2Δ0/+ met15dΔ0/+ ura3Δ0/ura3Δ0 NAT-GPDpr-MRPL28(1-30)-GFP-HIS*	Based on BY4743	diploid
YMS5729	GPDpr-RSM18(1-30)-GFP	*his3Δ1/his3Δ1 leu2Δ0/leu2Δ0 lys2Δ0/+ met15dΔ0/+ ura3Δ0/ura3Δ0 NAT-GPDpr-RSM18(1-30)-GFP-HIS*	Based on BY4743	diploid
YMS5730	GPDpr-YNL122C(1-30)-GFP	*his3Δ1/his3Δ1 leu2Δ0/leu2Δ0 lys2Δ0/+ met15dΔ0/+ ura3Δ0/ura3Δ0 NAT-GPDpr-YNL122C(1-30)-GFP-HIS*	Based on BY4743	diploid
YMS5731	GPDpr-MRP20(1-30)-GFP	*his3Δ1/his3Δ1 leu2Δ0/leu2Δ0 lys2Δ0/+ met15dΔ0/+ ura3Δ0/ura3Δ0 NAT-GPDpr-MRP20(1-30)-GFP-HIS*	Based on BY4743	diploid
YMS5801	TPIpr-EcS6(ΔC)-GFP	*his3Δ1 leu2Δ0 met15Δ0 ura3Δ0 ho::TPIpr-EcS6(ΔC)-GFP-*	Based on BY4741	a
YMS5802	TPIpr-EcS6(ΔE)-GFP	*his3Δ1 leu2Δ0 met15Δ0 ura3Δ0 ho::TPIpr-EcS6(ΔE)-GFP-*	Based on BY4741	a
YMS5822	TPIpr-EcS6(4R)-GFP	*his3Δ1 leu2Δ0 met15Δ0 ura3Δ0 ho::TPIpr-EcS6(4R)-GFP-*	Based on BY4741	a
YMS5823	TPIpr-EcS6(TBM1)-GFP	*his3Δ1 leu2Δ0 met15Δ0 ura3Δ0 ho::TPIpr-EcS6(TBM1)-GFP-*	Based on BY4741	a
YMS5824	TPIpr-EcS6(TBM1-2)-GFP	*his3Δ1 leu2Δ0 met15Δ0 ura3Δ0 ho::TPIpr-EcS6(TBM1,2)-*	Based on BY4741	a
YMS5825	TPIpr-EcS6(Loop2)-GFP	*his3Δ1 leu2Δ0 met15Δ0 ura3Δ0 ho::TPIpr-EcS6(Loop2)-GFP-*	Based on BY4741	a
YMS5826	TPIpr-EcS6(TBM1,2-Loop2)-GFP	*his3Δ1 leu2Δ0 met15Δ0 ura3Δ0 ho::TPIpr-EcS6(TBM1,2-*	Based on BY4741	a
YMS5827	TPIpr-EcS6(TBM1,2-Loop2-ΔE)-GFP	*his3Δ1 leu2Δ0 met15Δ0 ura3Δ0 ho::TPIpr-EcS6(TBM1,2-*	Based on BY4741	a
YMS5908	GPDpr-MRP17(21-131)-GFP	*his3Δ1/his3Δ1 leu2Δ0/leu2Δ0 met15Δ0/met15Δ0 ura3Δ0/ura3Δ0 NAT-GPDpr-MRP17(21-131)-GFP-*	Based on BY4743	diploid
YMS5909	GPDpr-MRP17(41-131)-GFP	*his3Δ1/his3Δ1 leu2Δ0/leu2Δ0 met15Δ0/met15Δ0 ura3Δ0/ura3Δ0 NAT-GPDpr-MRP17(41-131)-GFP-*	Based on BY4743	diploid
YMS5910	GPDpr-MRP17(61-131)-GFP	*his3Δ1/his3Δ1 leu2Δ0/leu2Δ0 met15Δ0/met15Δ0 ura3Δ0/ura3Δ0 NAT-GPDpr-MRP17(61-131)-GFP-*	Based on BY4743	diploid
YMS5911	GPDpr-MRP17(81-131)-GFP	*his3Δ1/his3Δ1 leu2Δ0/leu2Δ0 met15Δ0/met15Δ0 ura3Δ0/ura3Δ0 NAT-GPDpr-MRP17(81-131)-GFP-*	Based on BY4743	diploid
YMS5912	GPDpr-MRP17(101-131)-GFP	*his3Δ1/his3Δ1 leu2Δ0/leu2Δ0 met15Δ0/met15Δ0 ura3Δ0/ura3Δ0 NAT-GPDpr-MRP17(101-131)-GFP-*	Based on BY4743	diploid
YMS5913	GPDpr-MRP17(21-40)-GFP	*his3Δ1/his3Δ1 leu2Δ0/leu2Δ0 met15Δ0/met15Δ0 ura3Δ0/ura3Δ0 NAT-GPDpr-MRP17(21-40)-GFP-*	Based on BY4743	diploid
YMS5914	GPDpr-MRP17(21-60)-GFP	*his3Δ1/his3Δ1 leu2Δ0/leu2Δ0 met15Δ0/met15Δ0 ura3Δ0/ura3Δ0 NAT-GPDpr-MRP17(21-60)-GFP-*	Based on BY4743	diploid
YMS5915	GPDpr-MRP17(21-80)-GFP	*his3Δ1/his3Δ1 leu2Δ0/leu2Δ0 met15Δ0/met15Δ0 ura3Δ0/ura3Δ0 NAT-GPDpr-MRP17(21-80)-GFP-*	Based on BY4743	diploid
YMS5916	GPDpr-MRP17(21-100)-GFP	*his3Δ1/his3Δ1 leu2Δ0/leu2Δ0 met15Δ0/met15Δ0 ura3Δ0/ura3Δ0 NAT-GPDpr-MRP17(21-100)-GFP-*	Based on BY4743	diploid

YMS5917	GPDpr-MRP17(41-60)-GFP	*his3Δ1/his3Δ1 leu2Δ0/leu2Δ0 met15Δ0/met15Δ0 ura3Δ0/ura3Δ0 NAT-GPDpr-MRP17(41-60)-GFP-*	Based on BY4743	diploid
YMS5918	GPDpr-MRP17(41-80)-GFP	*his3Δ1/his3Δ1 leu2Δ0/leu2Δ0 met15Δ0/met15Δ0 ura3Δ0/ura3Δ0 NAT-GPDpr-MRP17(41-80)-GFP-*	Based on BY4743	diploid
YMS5919	GPDpr-MRP17(41-100)-GFP	*his3Δ1/his3Δ1 leu2Δ0/leu2Δ0 met15Δ0/met15Δ0 ura3Δ0/ura3Δ0 NAT-GPDpr-MRP17(41-100)-GFP-*	Based on BY4743	diploid
YMS5920	GPDpr-MRP17(61-80)-GFP	*his3Δ1/his3Δ1 leu2Δ0/leu2Δ0 met15Δ0/met15Δ0 ura3Δ0/ura3Δ0 NAT-GPDpr-MRP17(61-80)-GFP-*	Based on BY4743	diploid
YMS5921	GPDpr-MRP17(61-100)-GFP	*his3Δ1/his3Δ1 leu2Δ0/leu2Δ0 met15Δ0/met15Δ0 ura3Δ0/ura3Δ0 NAT-GPDpr-MRP17(61-100)-GFP-*	Based on BY4743	diploid
YMS5922	GPDpr-MRP17(81-100)-GFP	*his3Δ1/his3Δ1 leu2Δ0/leu2Δ0 met15Δ0/met15Δ0 ura3Δ0/ura3Δ0 NAT-GPDpr-MRP17(81-100)-GFP-*	Based on BY4743	diploid
YMS6072	MRPL23Δ30-GFP NOP2-mCherry	*YMS5691 NOP2-mCherry-KAN*	Based on YMS5691	diploid
YMS6073	MRP20Δ30-GFP NOP2-mCherry	*YMS5700 NOP2-mCherry-KAN*	Based on YMS5700	diploid
YMS6074	RSM18Δ30-GFP NOP2-mCherry	*YMS5698 NOP2-mCherry-KAN*	Based on YMS5698	diploid
YMS6134	RSM27Δ30-GFP NOP2-mCherry	*YMS5689 NOP2-mCherry-KAN*	Based on YMS5689	diploid
YMS6135	WT Mrp17-GFP	*W303 ho::TPIpr-MRP17-GFP-NAT*	Based on HHY1105	a
YMS6136	Δtom20 Mrp17-GFP-1	*W303 TOM20::HIS ho::TPIpr-MRP17-GFP-NAT*	Based on HHY1819	a

References

Becker, T., Wenz, L.-S., Krüger, V., Lehmann, W., Müller, J. M., Goroncy, L., Zufall, N., Lithgow, T., Guiard, B., Chacinska, A., Wagner, R., Meisinger, C., & Pfanner, N. (2011). The mitochondrial import protein Mim1 promotes biogenesis of multispanning outer membrane proteins. *Journal of Cell Biology*, *194* (3), 387–395

Brachmann, C. B., Davies, A., Cost, G. J., Caputo, E., Li, J., Hieter, P., & Boeke, J. D. (1998). Designer deletion strains derived from Saccharomyces cerevisiae S288C: A useful set of strains and plasmids for PCR-mediated gene disruption and other applications. *Yeast*, *14* (2), 115–132.

Cohen, Y., & Schuldiner, M. (2011). Advanced methods for high-throughput microscopy screening of genetically modified yeast libraries. *Methods in Molecular Biology (Clifton, N.J.)*, *781*, 127–159.

Jores, T., Lawatscheck, J., Beke, V., Franz-Wachtel, M., Yunoki, K., Fitzgerald, J. C., Macek, B., Endo, T., Kalbacher, H., Buchner, J., & Rapaport, D. (2018). Cytosolic Hsp70 and Hsp40 chaperones enable the biogenesis of mitochondrial β-barrel proteins. *The Journal of Cell Biology*, *217* (9), 3091–3108

Müller, J. E. N., Papic, D., Ulrich, T., Grin, I., Schütz, M., Oberhettinger, P., Tommassen, J., Linke, D., Dimmer, K. S., Autenrieth, I. B., & Rapaport, D. (2011). Mitochondria can recognize and assemble fragments of a β-barrel structure. *Molecular Biology of the Cell*, *22* (10), 1638–1647.

Appendix Table S2. Plasmids used in this study.

Plasmid number	Plasmid Name	Description	Plasmid use	Reference
HHB1249	pGem4-Mrp17	In vitro expression	Radiolabeling, in vitro import	This study
HHB1357	pGem4-Mrp17K-A-DHFRmut	In vitro expression	Radiolabeling, in vitro import	This study
HHB1358	pGem4-Mrp17K-R-DHFRmut	In vitro expression	Radiolabeling, in vitro import	This study
HHB1359	pGem4-Mrp17-DHFRmut (30-60)	In vitro expression	Radiolabeling, in vitro import	This study
HHB1398	pGem4-Atp1	In vitro expression	Radiolabeling, in vitro import	This study
HHB1438	pGem4-Mrp17K-A(1-60)-DHFRmut	In vitro expression	Radiolabeling, in vitro import	This study
HHB1439	pGem4-Mrp17K-A(30-60)-DHFRmut	In vitro expression	Radiolabeling, in vitro import	This study
HHB1440	pGem4-Mrp17TBM1,2mut-DHFRmut	In vitro expression	Radiolabeling, in vitro import	This study
HHB1486	pGem4-EcS6-DHFRmut	In vitro expression	Radiolabeling, in vitro import	This study
HHB1489	pGem4-S6/Mrp17TS	In vitro expression	Radiolabeling, in vitro import	This study
HHB1491	pYX232 - Mrp17	in vivo expression, CEN/ARS plasmid	Growth rescue by plasmid shuffling	This study
HHB1492	pYX232 - Mrp17(K-A)	in vivo expression, CEN/ARS plasmid	Growth rescue by plasmid shuffling	This study
HHB1493	pYX232 - Mrp17(K-R)	in vivo expression, CEN/ARS plasmid	Growth rescue by plasmid shuffling	This study
HHB1647	pYX232 - empty vector	in vivo expression, CEN/ARS plasmid	Growth rescue by plasmid shuffling	Commercial (Novagen)
HHB1648	pGem4-Mrp17-DHFRmut (1-131)	In vitro expression	Radiolabeling, in vitro import	This study
HHB1649	pGem4-Mrp17-DHFRmut (1-10)	In vitro expression	Radiolabeling, in vitro import	This study
HHB1650	pGem4-Mrp17-DHFRmut (1-20)	In vitro expression	Radiolabeling, in vitro import	This study
HHB1651	pGem4-Mrp17-DHFRmut (1-40)	In vitro expression	Radiolabeling, in vitro import	This study
HHB1652	pGem4-Mrp17-DHFRmut (1-60)	In vitro expression	Radiolabeling, in vitro import	This study
HHB1653	pGem4-Mrp17-DHFRmut (21-131)	In vitro expression	Radiolabeling, in vitro import	This study
HHB1654	pGem4-Mrp17-DHFRmut (41-131)	In vitro expression	Radiolabeling, in vitro import	This study
HHB1655	pGem4-Mrp17-DHFRmut (101-131)	In vitro expression	Radiolabeling, in vitro import	This study
HHB2010	pGem4 - S6/Mrp17TS+	Revision, in vitro import		This study
HHB2013	pGem4 - Mrp17TBD1mut-DHFRmut	Revision, in vitro import		This study
HHB2014	pGem4 - Mrp17TBD2mut-DHFRmut	Revision, in vitro import		This study
HHB2016	pYX232 - Su9-Mrp17	Revision, drop dilution		This study
HHB2018	pYX232 - Su9-Mrp17(K-A)	Revision, drop dilution		This study
pMS0021	pFA6-GFP-HIS	For PCR based mutagenesis, C-terminal GFP tagging	For C-terminal truncation and GFP tagging	Longtine et al 1998
pMS207	NLS-tdTomato	Protein expression	Nuclear marker	Kindly provided by Daniel kaganovich*
pMS233	pBS34 mCherry-KAN	For PCR based mutagenesis, C-terminal mCherry tagging		Kindly provided by Naama Barkai**
pMS0351	pYM-N15 NAT-GPDpr	For PCR based mutagenesis, promotor swap	Used to create N-terminal MRP truncations and	Janke et al 2004
pMS901	pFA6 TPIpr-MRP17-GFP-NAT	For cloning and protein expression in yeast	Inserting Mrp17-GFP in the HO locus for expression	This study
pMS1040	pYX142 TPIpr-Mrp17-GFP-LEU	in vivo expression, CEN/ARS plasmid	In vivo expression and targeting	This study
pMS1041	pYX142 TPIpr-Mrp17(Arg)-GFP-LEU	in vivo expression, CEN/ARS plasmid	In vivo expression and targeting	This study
pMS1042	pYX142 TPIpr-Mrp17(Ala)-GFP-LEU	in vivo expression, CEN/ARS plasmid	In vivo expression and targeting	This study
pMS1176	pFA6 TPIpr-EcL13-GFP	For PCR based insertion into genomic locus and expression from there	In vivo expression and targeting	This study
pMS1177	pFA6 TPIpr-EcL14-GFP	For PCR based insertion into genomic locus and expression from there	In vivo expression and targeting	This study
pMS1179	pFA6 TPIpr-EcS6-GFP	For PCR based insertion into genomic locus and expression from there	In vivo expression and targeting	This study
pMS1181	pFA6 TPIpr-EcS16-GFP	For PCR based insertion into genomic locus and expression from there	In vivo expression and targeting	This study
pMS1182	pFA6 TPIpr-EcS6(ΔC)-GFP	For PCR based insertion into genomic locus and expression from there	In vivo expression and targeting	This study
pMS1183	pFA6 TPIpr-EcS6(ΔE)-GFP	For PCR based insertion into genomic locus and expression from there	In vivo expression and targeting	This study
pMS1206	pFA6 TPIpr-EcS6(4R)-GFP	For PCR based insertion into genomic locus and expression from there	In vivo expression and targeting	This study
pMS1207	pFA6 TPIpr-EcS6(TBM1)-GFP	For PCR based insertion into genomic locus and expression from there	In vivo expression and targeting	This study
pMS1208	pFA6 TPIpr-EcS6(TBM1,2)-GFP	For PCR based insertion into genomic locus and expression from there	In vivo expression and targeting	This study

pMS1209	pFA6 TPIpr-EcS6(Loop2)-GFP	For PCR based insertion into genomic locus and expression from there	In vivo expression and targeting	This study
pMS1210	pFA6 TPIpr-EcS6(TBM1,2-Loop2)-GFP	For PCR based insertion into genomic locus and expression from there	In vivo expression and targeting	This study
pMS1211	pFA6 TPIpr-EcS6(TBM1,2-Loop2-ΔE)-GFP	For PCR based insertion into genomic locus and expression from there	In vivo expression and targeting	This study

* Prof. Daniel Kaganovich, Göttingen University, Germany
** Prof. Naama Barkai, Weizmann Institute of Science, Israel

References

Longtine, M.S., A. McKenzie, D.J. Demarini, N.G. Shah, A. Wach, A. Brachat, P. Philippsen, and J.R. Pringle. 1998. Additional modules for versatile and economical PCR-based gene deletion and modification in Saccharomyces cerevisiae. *Yeast* . 14(10):953–961.

Janke, C., M.M. Magiera, N. Rathfelder, C. Taxis, S. Reber, H. Maekawa, A. Moreno-Borchart, G. Doenges, E. Schwob, E. Schiebel, and M. Knop. 2004. A versatile toolbox for PCR-based tagging of yeast genes: New fluorescent proteins, more markers and promoter substitution cassettes. *Yeast* . 21(11):947-962

Appendix Table S3. Primers used in this study for yeast transformation.

Primer number	Primer name	Sequence*	Description
5381	MRP17 Ctag Full F	GTCTATTTTAGAATTAGTGAATGAAGATTATCAATCCATTcggatccccgggttaattaa	For tagging with GFP at the C-terminus usng pMS21
7273	MRPL38 Ctag Full F	GGGATACAATAAGATATGCTCTTTGGCAAGTAGGGTCATAcggatccccgggttaattaa	For tagging with GFP at the C-terminus usng pMS21
7274	RSM25 Ctag Full F	GCAAGAAGAAGAATCCACCACTTCCGAGAACCTCCACTTCcggatccccgggttaattaa	For tagging with GFP at the C-terminus usng pMS21
7275	RSM27 Ctag Full F	TGCAGCTGCAACTGCAAAGGGGAAGGGGAAGAAAAAAAAAcggatccccgggttaattaa	For tagging with GFP at the C-terminus usng pMS21
7276	MRP51 Ctag Full F	TCAAGAGGCCAATAACCTTTTGAATATAATCAAGGGAAATcggatccccgggttaattaa	For tagging with GFP at the C-terminus usng pMS21
5627	MRPL23 Ctag Full F	ACCAGAGCCTCTTAAAGAATCAATTTTTAACCAATTGAAAcggatccccgggttaattaa	For tagging with GFP at the C-terminus usng pMS21
7277	MRPL40 Ctag Full F	GGCGAGGGTTTTTGAATTTTTGGAAAAACAAAAAAGAGAAcggatccccgggttaattaa	For tagging with GFP at the C-terminus usng pMS21
7278	MRPS16 Ctag Full F	GAATAGGAAAGTTGTATTTGAAAGAATGGAAACATTGGAGcggatccccgggttaattaa	For tagging with GFP at the C-terminus usng pMS21
7279	PET123 Ctag Full F	AGCTGGAAATAATGGTAATAATAATAATACAACCAACTTGcggatccccgggttaattaa	For tagging with GFP at the C-terminus usng pMS21
7280	RSM26 Ctag Full F	GTCAGTGGTAAATAATAGGATATTCTCTGGTATATCGAAGcggatccccgggttaattaa	For tagging with GFP at the C-terminus usng pMS21
7281	MRPL15 Ctag Full F	AAAGGCAAAAATATCCGACAAGGAAAATAAAGCATTTCTGcggatccccgggttaattaa	For tagging with GFP at the C-terminus usng pMS21
7282	MRPL28 Ctag Full F	CTTCCCCCCAAATACGTTATGGCATTATAACTTCCGAAAAcggatccccgggttaattaa	For tagging with GFP at the C-terminus usng pMS21
7283	RSM18 Ctag Full F	ATCGAAAGCCATTAGACGGTGCCAGGCAATAGGATTAATGcggatccccgggttaattaa	For tagging with GFP at the C-terminus usng pMS21
7284	MRP35 Ctag Full F	AGCTTATTCAAAGCATCTAAAGCGGTTATTACCCTATCATcggatccccgggttaattaa	For tagging with GFP at the C-terminus usng pMS21
7285	MRP20 Ctag Full F	TAAACTAATTGCCCTGAACAGGTACATAGAAGATCTACATcggatccccgggttaattaa	For tagging with GFP at the C-terminus usng pMS21
7286	MRP17 Ctag (1-30) F	AAAGTTGGAAGCAAAAGAATTGTCTTCCACAATCGGGAAAcggatccccgggttaattaa	For tagging with GFP after amino acid 30 using pMS21
7287	MRPL38 Ctag (1-30) F	ATTAGCAGAATGTATTAAAGTAATAAGGAAAGGGTCCCCCcggatccccgggttaattaa	For tagging with GFP after amino acid 30 using pMS21
7288	RSM25 Ctag (1-30) F	CTTGAAATCAGGATTGTTGAAAGAAACACCTGCGTGGTACcggatccccgggttaattaa	For tagging with GFP after amino acid 30 using pMS21
7289	RSM27 Ctag (1-30) F	GATATTTGATCAAAATTTCAACCCATCAGGTATAAGAACCcggatccccgggttaattaa	For tagging with GFP after amino acid 30 using pMS21
7290	MRP51 Ctag (1-30) F	CAATCATAAACCATTGACATACACTGGCAAAAAATTTCATcggatccccgggttaattaa	For tagging with GFP after amino acid 30 using pMS21
7291	MRPL23 Ctag (1-30) F	CCATGTCGACGTTGCCCGTGATAAGAGGACATTGGGTAGAcggatccccgggttaattaa	For tagging with GFP after amino acid 30 using pMS21
7292	MRPL40 Ctag (1-30) F	AAGATTGGGGAATCGACCCAAAAACTTTCTTCCTCATAGTcggatccccgggttaattaa	For tagging with GFP after amino acid 30 using pMS21
7293	MRPS16 Ctag (1-30) F	TAGTCCGGTCTATAATATCGTAGTGGCTAATTCCCGTAAGcggatccccgggttaattaa	For tagging with GFP after amino acid 30 using pMS21
7294	PET123 Ctag (1-30) F	AACAAGGTCCATTCTGAAGAGCCCCACTACAAAGCAGACTcggatccccgggttaattaa	For tagging with GFP after amino acid 30 using pMS21
7295	RSM26 Ctag (1-30) F	TAAGGCCCTACTGCAAAATGGAGTGCCTAATATACTCAGTcggatccccgggttaattaa	For tagging with GFP after amino acid 30 using pMS21
7296	MRPL15 Ctag (1-30) F	TGCTTTGAACTGCAATTTACAAAGCGTTAGAACAGTCATAcggatccccgggttaattaa	For tagging with GFP after amino acid 30 using pMS21
7297	MRPL28 Ctag (1-30) F	ATCTGGGACCACGGTCTTCATCAGAAATAAGAGAACAAAGcggatccccgggttaattaa	For tagging with GFP after amino acid 30 using pMS21
7298	RSM18 Ctag (1-30) F	ATACAATTTCGGTATCAAAGAAAAGAAGAGTGTCAATATTcggatccccgggttaattaa	For tagging with GFP after amino acid 30 using pMS21
7299	MRP35 Ctag (1-30) F	CATGTCTGTTTTCAATGTACTCAAACCACTACTTAAAGGTcggatccccgggttaattaa	For tagging with GFP after amino acid 30 using pMS21
7300	MRP20 Ctag (1-30) F	AACACTTGCAGTAGGAAGTTGCAAGCCAGAGCAGGTTCCAcggatccccgggttaattaa	For tagging with GFP after amino acid 30 using pMS21
5382	MRP17 Ctag R	GTTTGTAGCGTCCCCAGTCAAAACCTGTGCGCTATACCTAgaattcgagctcgtttaaac	Reverse primer both for full-length C-terminal GFP tagging and for truncation after aa 30,
7301	MRPL38 Ctag R	TAACGTTAATGAATGGAAAATGGGGGTACTTTTATTCTTAgaattcgagctcgtttaaac	Reverse primer both for full-length C-terminal GFP tagging and for truncation after aa 30,
6425	RSM25 Ctag R	GTTGGTTAGGTGACGTTGGAGAAAAGAGGCAGCCCACCTAgaattcgagctcgtttaaac	Reverse primer both for full-length C-terminal GFP tagging and for truncation after aa 30,
7302	RSM27 Ctag R	TACATAATGAATATTTTTTTTAGTAAGGGTGCTCCAATCAgaattcgagctcgtttaaac	Reverse primer both for full-length C-terminal GFP tagging and for truncation after aa 30,
7303	MRP51 Ctag R	AATATACATTGATTGTTCACATTTTTTTTTTTCTCTTCTCAgaattcgagctcgtttaaac	Reverse primer both for full-length C-terminal GFP tagging and for truncation after aa 30,
5628	MRPL23 Ctag R	AAGGATATATAAAAAAATGACAAATAATTTCAATAAGCTAgaattcgagctcgtttaaac	Reverse primer both for full-length C-terminal GFP tagging and for truncation after aa 30,
7304	MRPL40 Ctag R	ATCAAATATAAAAATAGAGTGCATACACTTGGTACATTTAgaattcgagctcgtttaaac	Reverse primer both for full-length C-terminal GFP tagging and for truncation after aa 30,
7305	MRPS16 Ctag R	CCTTTCTTTTTCTTTTTTTTTTTTTTTCCGATCTTTTTCAgaattcgagctcgtttaaac	Reverse primer both for full-length C-terminal GFP tagging and for truncation after aa 30,

7306	PET123 Ctag R	ATACATGACATGTTGAAGTAGTATATATATTAGTTATTTAgaattcgagctcgtttaaac	Reverse primer both for full-length C-terminal GFP tagging and for truncation after aa 30,
7307	RSM26 Ctag R	ATATGAGGGTAAGAGGCTATCGCTTCCTTTCTTTTCATTAgaattcgagctcgtttaaac	Reverse primer both for full-length C-terminal GFP tagging and for truncation after aa 30,
7308	MRPL15 Ctag R	TTTGGCCAGTGAACCATCAACTATATTTTTTGTATCTTCAgaattcgagctcgtttaaac	Reverse primer both for full-length C-terminal GFP tagging and for truncation after aa 30,
7309	MRPL28 Ctag R	TTTACAGCAGGAGACAGTCAAGCGTTCGCTCACGCTATCAgaattcgagctcgtttaaac	Reverse primer both for full-length C-terminal GFP tagging and for truncation after aa 30,
7310	RSM18 Ctag R	AATTATTTCATGTGGAGAAGCATTAGTGTAGTACAGTTCAgaattcgagctcgtttaaac	Reverse primer both for full-length C-terminal GFP tagging and for truncation after aa 30,
7311	MRP35 Ctag R	TGCGTTTGGTTTAGAGGAGAACATAATTATTCCACCTTCAgaattcgagctcgtttaaac	Reverse primer both for full-length C-terminal GFP tagging and for truncation after aa 30,
7312	MRP20 Ctag R	GCGTGTGGGTTAACGAAGGGGTGGAATGGGAAACCTTCTAgaattcgagctcgtttaaac	Reverse primer both for full-length C-terminal GFP tagging and for truncation after aa 30,
5432	MRP17 Ntag F	GAGGAAAGAATAACGCAAAACCACCCTTTTCACTAAGATGcgtacgctgcaggtcgac	Insertion of GPD promotor with optional N-terminal truncation (depends on R primer) for
7361	MRPL38 Ntag F	TGAGATCTGGATAAGCCATATTTTCTAACATCACAACATGcgtacgctgcaggtcgac	Insertion of GPD promotor with optional N-terminal truncation (depends on R primer) for
7362	RSM25 Ntag F	CGCACCATTCAAGAAGAAGATCTTAGAATTCTGAGAAATGcgtacgctgcaggtcgac	Insertion of GPD promotor with optional N-terminal truncation (depends on R primer) for
7363	RSM27 Ntag F	TAGTGATTGCGTGGATTAGTTTATCATAAGACTTTCAATGcgtacgctgcaggtcgac	Insertion of GPD promotor with optional N-terminal truncation (depends on R primer) for
7364	MRP51 Ntag F	TTTTTCTTTTTCTTTATTTTAGAAAAAATTGAACGAAATGcgtacgctgcaggtcgac	Insertion of GPD promotor with optional N-terminal truncation (depends on R primer) for
7365	MRPL23 Ntag F	AAAAGAAAGCAATACCAGCATCAACAATAGCCTATTCATGcgtacgctgcaggtcgac	Insertion of GPD promotor with optional N-terminal truncation (depends on R primer) for
7366	MRPL40 Ntag F	TAAAGAACATTATCAGCAACAAAATTTGTAATCCATCATGcgtacgctgcaggtcgac	Insertion of GPD promotor with optional N-terminal truncation (depends on R primer) for
7367	MRPS16 Ntag F	GTATTCCGTTAAGAAATTGTAATAGAGTTTATCAAGTATGcgtacgctgcaggtcgac	Insertion of GPD promotor with optional N-terminal truncation (depends on R primer) for
7368	PET123 Ntag F	GATTGTCAACAACAATAATAACAAGGATAATTGAAGTATGcgtacgctgcaggtcgac	Insertion of GPD promotor with optional N-terminal truncation (depends on R primer) for
7338	RSM26 Ntag F	TAATATCGTCTTCTTGGCGCAACTAAGACGCAGAGAAATGcgtacgctgcaggtcgac	Insertion of GPD promotor with optional N-terminal truncation (depends on R primer) for
7339	MRPL15 Ntag F	GGTAGATGGAAAGTAAAGAGTAGACAAGCTCGGAAACATGcgtacgctgcaggtcgac	Insertion of GPD promotor with optional N-terminal truncation (depends on R primer) for
7340	MRPL28 Ntag F	ACTGACGAAGACCTACGCTACACAACGAAGATAACACATGcgtacgctgcaggtcgac	Insertion of GPD promotor with optional N-terminal truncation (depends on R primer) for
7369	RSM18 Ntag F	GATGAATGCCACCACAGGCATAAAAATTCAAAACGGTATGcgtacgctgcaggtcgac	Insertion of GPD promotor with optional N-terminal truncation (depends on R primer) for
7370	MRP35 Ntag F	CTGGTCAAATACAAAAAAGATAGTATATATATCTATCATGcgtacgctgcaggtcgac	Insertion of GPD promotor with optional N-terminal truncation (depends on R primer) for
7341	MRP20 Ntag F	AAATAAAAATAAAAAGTACGCAACACCAACACAGAATATGcgtacgctgcaggtcgac	Insertion of GPD promotor with optional N-terminal truncation (depends on R primer) for
5433	MRP17 Ntag R	TGGAGTTGGTTATGCGAACAAGTCCGATCAGCTCATAAAGcatcgatgaattctctgtcg	Insertion of GPD promotor without N-terminal truncation with
7371	MRPL38 Ntag R	CTGAATTGTCGATTACCTTGATGACAGATTTTAGAAATATcatcgatgaattctctgtcg	Insertion of GPD promotor without N-terminal truncation with
7372	RSM25 Ntag R	ACGTCCGTTGAAGGACGTTTACAGCATTTGTTTGTATCTTcatcgatgaattctctgtcg	Insertion of GPD promotor without N-terminal truncation with
7373	RSM27 Ntag R	ATAACTCTGCTACTTTTAGGAGTCTCGCCTTTGGTACGTTcatcgatgaattctctgtcg	Insertion of GPD promotor without N-terminal truncation with
7374	MRP51 Ntag R	CTTGAGCTATTCTCGACCGTCCAAGCAACTCTGCCAATGTcatcgatgaattctctgtcg	Insertion of GPD promotor without N-terminal truncation with
7375	MRPL23 Ntag R	GGCGCGCAAAAGCCAAGCCACTGTGTCCAATCTTCTGCGAcatcgatgaattctctgtcg	Insertion of GPD promotor without N-terminal truncation with
7376	MRPL40 Ntag R	CACGCGATCCAACGTTTGATAAATGTTGATAGCTTCCAGAcatcgatgaattctctgtcg	Insertion of GPD promotor without N-terminal truncation with
7377	MRPS16 Ntag R	TTCCAAATCTAGCTAACCTTATTCGTACTAGACCACAGGTcatcgatgaattctctgtcg	Insertion of GPD promotor without N-terminal truncation with
7378	PET123 Ntag R	AAACCCCACTTTTAAAACCATACTTGGCCGCACCTTTCCCcatcgatgaattctctgtcg	Insertion of GPD promotor without N-terminal truncation with
7342	RSM26 Ntag R	GTAGTTTAGGTACTACGTGAATACCCCTTTTGAAAACAAGcatcgatgaattctctgtcg	Insertion of GPD promotor without N-terminal truncation with

7343	MRPL15 Ntag R	GTCTCCGCAAGGAACGCGATATAAACATCATGCTATTTTCcatcgatgaattctctgtcg	Insertion of GPD promotor without N-terminal truncation with
7344	MRPL28 Ntag R	CTAGAACGGCTCTGTGTGGTTTTTTGAATGTTTGTGCCAGcatcgatgaattctctgtcg	Insertion of GPD promotor without N-terminal truncation with
7379	RSM18 Ntag R	GTTTAAATGTGGAGCTTACTGCTCCCTTGATGATAGGTTGcatcgatgaattctctgtcg	Insertion of GPD promotor without N-terminal truncation with
7380	MRP35 Ntag R	TTTGGTCTCCTCGTTGCCGTTTGTTATGCAAGCTAATTTTcatcgatgaattctctgtcg	Insertion of GPD promotor without N-terminal truncation with
7345	MRP20 Ntag R	AAGGATACAACATATTCTTCGTTCCAACCGTCAATCGTGGcatcgatgaattctctgtcg	Insertion of GPD promotor without N-terminal truncation with
7346	MRP17 NtagΔ30 R	GTACAATGTCCCTCACCACACCTCTGTTTTGAATAATCAGcatcgatgaattctctgtcg	Insertion of GPD promotor with N-terminal truncation of 30 aa with
7347	MRPL38 NtagΔ30 R	TAACACAGACTATTCTGTCTCCAACCATTGCAGGACTCTTcatcgatgaattctctgtcg	Insertion of GPD promotor with N-terminal truncation of 30 aa with
7348	RSM25 NtagΔ30 R	GTGTAAACTTGGTTGATGGTGGGATAGACGCTACAACGTTcatcgatgaattctctgtcg	Insertion of GPD promotor with N-terminal truncation of 30 aa with
7349	RSM27 NtagΔ30 R	CAGAAGGGCCCTTTAATCGCTCATTTAGAATCTTAGAACCcatcgatgaattctctgtcg	Insertion of GPD promotor with N-terminal truncation of 30 aa with
7350	MRP51 NtagΔ30 R	ATAGTGTCGATGGCTTAGTTTCGATGATTTGATGTGTTGGcatcgatgaattctctgtcg	Insertion of GPD promotor with N-terminal truncation of 30 aa with
7351	MRPL23 NtagΔ30 R	TATGTCTACCAATTAAGGTAATTGCAATTGCTGAAGCCAAcatcgatgaattctctgtcg	Insertion of GPD promotor with N-terminal truncation of 30 aa with
7352	MRPL40 NtagΔ30 R	ACTTCATGAACTCAGGCGTAGATTTTTTAATGAACTTTTCcatcgatgaattctctgtcg	Insertion of GPD promotor with N-terminal truncation of 30 aa with
7353	MRPS16 NtagΔ30 R	CGTAGGTTCCTAGTACCTCGATCGGTTTAGCATCCCTCGCcatcgatgaattctctgtcg	Insertion of GPD promotor with N-terminal truncation of 30 aa with
7354	PET123 NtagΔ30 R	CACCCTTGGGCTTGGGTGATTTTACCTTATTAATAATATCcatcgatgaattctctgtcg	Insertion of GPD promotor with N-terminal truncation of 30 aa with
7355	RSM26 NtagΔ30 R	ATCGCTGGTAGTCGAACCACACCGTCTTGAATCCCGAAGAcatcgatgaattctctgtcg	Insertion of GPD promotor with N-terminal truncation of 30 aa with
7356	MRPL15 NtagΔ30 R	CTCGTCTCAAACCATTAATTCTCGGACCCTTATGAAGGTAcatcgatgaattctctgtcg	Insertion of GPD promotor with N-terminal truncation of 30 aa with
7357	MRPL28 NtagΔ30 R	TGACGACCCTTTGTGCCAAAGGTGACAGTGAGCTCTTGCTcatcgatgaattctctgtcg	Insertion of GPD promotor with N-terminal truncation of 30 aa with
7358	RSM18 NtagΔ30 R	ACTGATCGATTTTCTTTGTTTGTTGCGTTCTCCCCATTTCcatcgatgaattctctgtcg	Insertion of GPD promotor with N-terminal truncation of 30 aa with
7359	MRP35 NtagΔ30 R	TGAATAGAAAGCCGTTGAGCTTCACTTTGAAACTATTTGAcatcgatgaattctctgtcg	Insertion of GPD promotor with N-terminal truncation of 30 aa with
7360	MRP20 NtagΔ30 R	TTTTGGAGGAACTCTCCACCACTGAGGCTAGTGATCTTATcatcgatgaattctctgtcg	Insertion of GPD promotor with N-terminal truncation of 30 aa with
5383	MRP17 1-20 Ctag F	ACTTGTTCGCATAACCAACTCCAATGCGCCAAAGTTGGAAcggatccccgggttaattaa	For tagging with GFP after amino acid 20 using pMS21
5385	MRP17 1-40 Ctag F	AATCGGGAAACTGATTATTCAAAACAGAGGTGTGGTGAGGcggatccccgggttaattaa	For tagging with GFP after amino acid 40 using pMS21
5387	MRP17 1-60 Ctag F	AAGGTATCTTCCTAAAATAATGAAGAAAGACCAGGAAAAAcggatccccgggttaattaa	For tagging with GFP after amino acid 60 using pMS21
5388	MRP17 1-80 Ctag F	TTTAATGCTGTTTGATTCATCAGCTGCGGTACAGTCTGAAcggatccccgggttaattaa	For tagging with GFP after amino acid 80 using pMS21
5389	MRP17 1-100 Ctag F	AGATCCCCGTGTCATAAGATCATCCATCGTTAAAGTTGATcggatccccgggttaattaa	For tagging with GFP after amino acid 100 using pMS21
5614	Mrp17 NtagΔ20 R	GAATAATCAGTTTCCCGATTGTGGAAGACAATTCTTTTGCcatcgatgaattctctgtcg	Insertion of GPD promotor with N-terminal truncation of 20 aa with pMS351
5615	Mrp17 NtagΔ40 R	TTATTTTAGGAAGATACCTTATGCCCATGGGTACAATGTCcatcgatgaattctctgtcg	Insertion of GPD promotor with N-terminal truncation of 40 aa with pMS351
5616	Mrp17 NtagΔ60 R	ATGAATCAAACAGCATTAAAAAGTGATATGCTCGAAAATGcatcgatgaattctctgtcg	Insertion of GPD promotor with N-terminal truncation of 60 aa with pMS351
5617	Mrp17 NtagΔ80 R	ATCTTATGACACGGGGATCTTTCTTTAAAGTTCTTAGAATcatcgatgaattctctgtcg	Insertion of GPD promotor with N-terminal truncation of 80 aa with pMS351
5618	Mrp17 NtagΔ100 R	AACGGTGTAACGATGAGGCTCTATCCAGCTGCTTATCTAAcatcgatgaattctctgtcg	Insertion of GPD promotor with N-terminal truncation of 100 aa with pMS351
3469	HO locus pFA F	AAATCCATATCCTCATAAGCAGCAATCAATTCTATCTATAcggatccccgggttaattaa	Insertion of pFA6 plasmid-based expression constructs into the HO locus
3470	HO locus pFA R	AAATTTTACTTTTATTACATACAACTTTTTAAACTAATATgaattcgagctcgtttaaac	Insertion of pFA6 plasmid-based expression constructs into the HO locus
4892	NOP2 Ctag pFA6 F	GGGTGTCAATCCAAAAGCTAAAAGACCTTCTAACGAAAAAcggatccccgggttaattaa	For C-terminal tagging with mCherry
4893	NOP2 Ctag pFA6 R	AACTATGCTAACATGATGCCACTACGTTTGTGGGAACTTAgaattcgagctcgtttaaac	For C-terminal tagging with mCherry

*Capital letters denote sequences complementrary to the genomic DNA and small letters denote sequences complementary to the amplified cassette
