# Supplementary material for: Widespread use of unconventional targeting signals in mitochondrial ribosome proteins
Source: EMBO J. 2021 Nov 17;41(1):e109519. doi: 10.15252/embj.2021109519 (PMC8724765; doi:10.15252/embj.2021109519)
Supplement: Supplementary file 2 — Expanded View Figures PDF [file EMBJ-41-e109519-s003.pdf]

Expanded View Figures

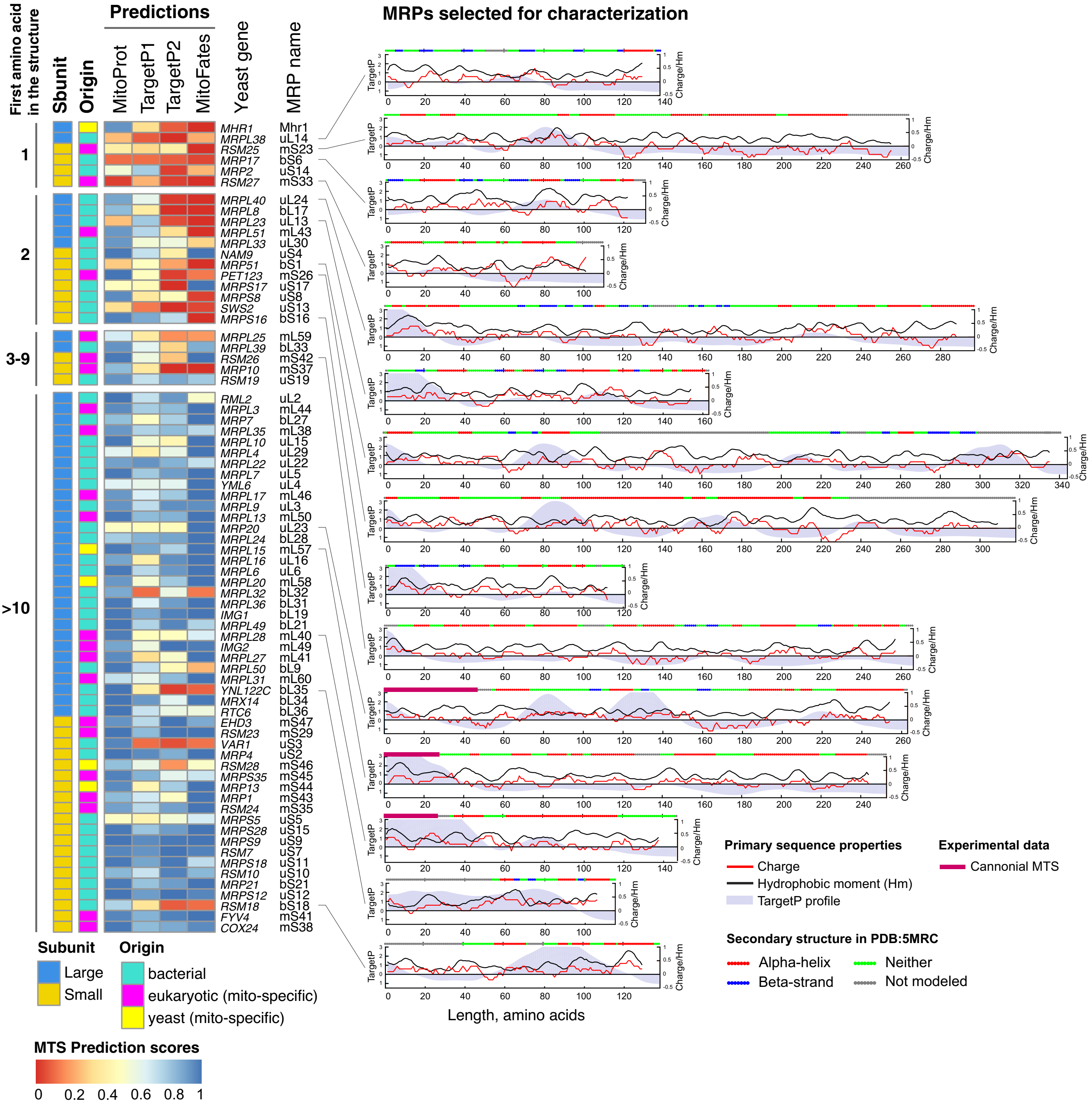

Figure EV1. MRPs can be classified according to the presence of their most N-terminus inside the mitoribosome structure.

Left—MTS prediction scores for yeast MRPs first sorted in groups by the first amino acid with reported atomic coordinates in the structure PDB:5MRC, then by subunit and then by length with protein origin and subunit noted for each MRP. Right—primary and secondary sequence properties for 15 MRPs selected for further characterization showing a variety of N-terminal and internal targeting signal predictions, overall positive charge, presence of documented cleavable MTS, and a variety of N-terminal secondary structures. Universal ribosomal protein nomenclature is used (Ban *et al*, 2014), except for Mhr1 which is a yeast-specific MRP.

© 2021 The Authors
16 of 15 *The EMBO Journal* 41: e109519 | 2021 © 2021 The Authors

Figure EV2. MRPs have N-termini with various targeting properties.

A MRP-GFP truncations mistargeted to the nucleus were transformed with NLS (nuclear localization signal)-tdTomato plasmid, stained with MitoView 405 dye and visualized by fluorescent microscopy. Scale bar is 10 μm.

B MRP-GFP truncations mistargeted to the nucleus and enriched in the nucleolus with nucleolar protein Nop2 genomically tagged using mCherry, stained with MitoView 405 dye and visualized by fluorescent microscopy. Observed MRP-GFP aggregates are highlighted with white arrowheads. Scale bar is 10 μm.

C Summary of mistargeting locations for one or more truncations of each MRP, if the location is observed for any of the MRP truncations, it is marked with a black circle.

© 2021 The Authors

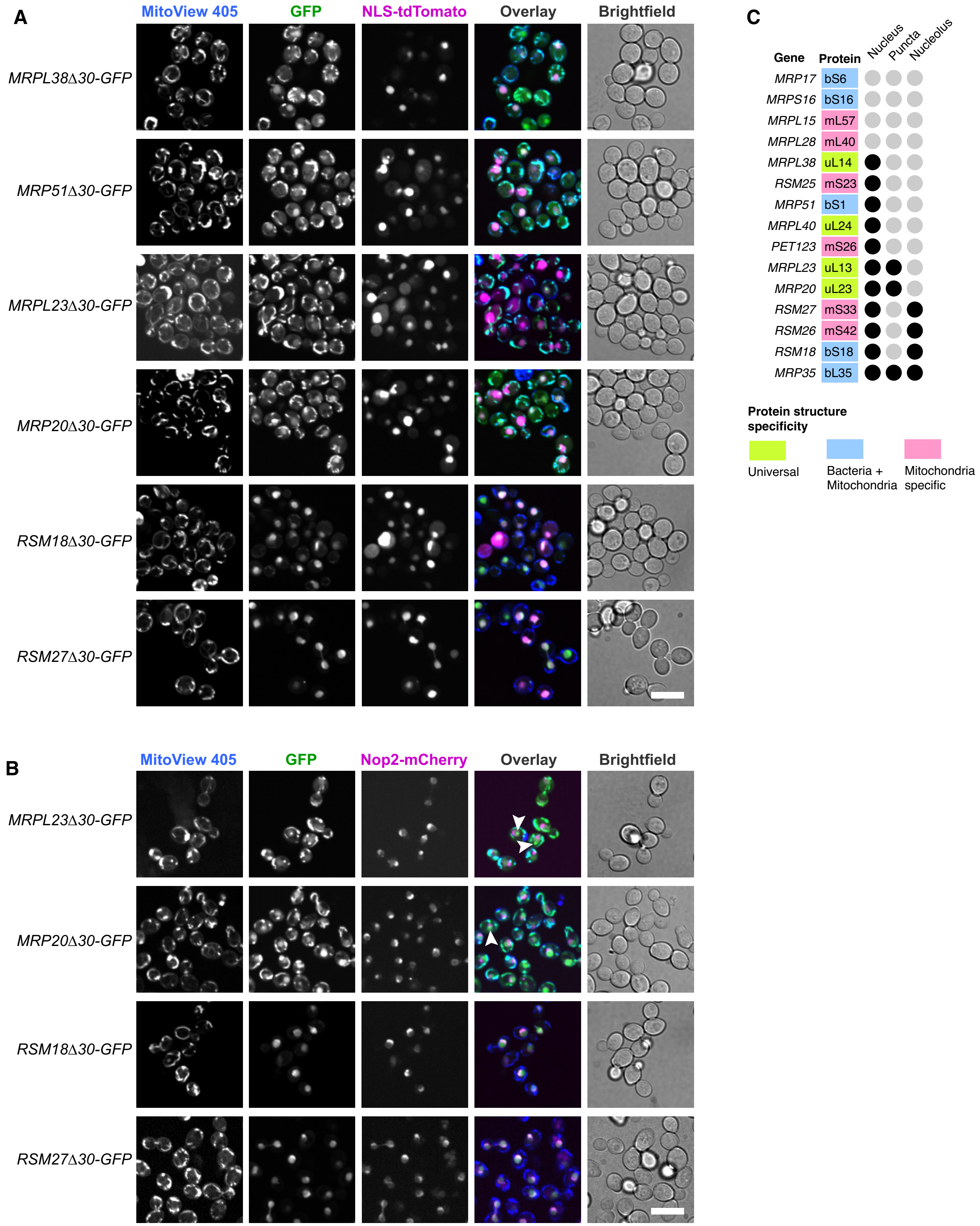

Figure EV2.

 © 2021 The Authors

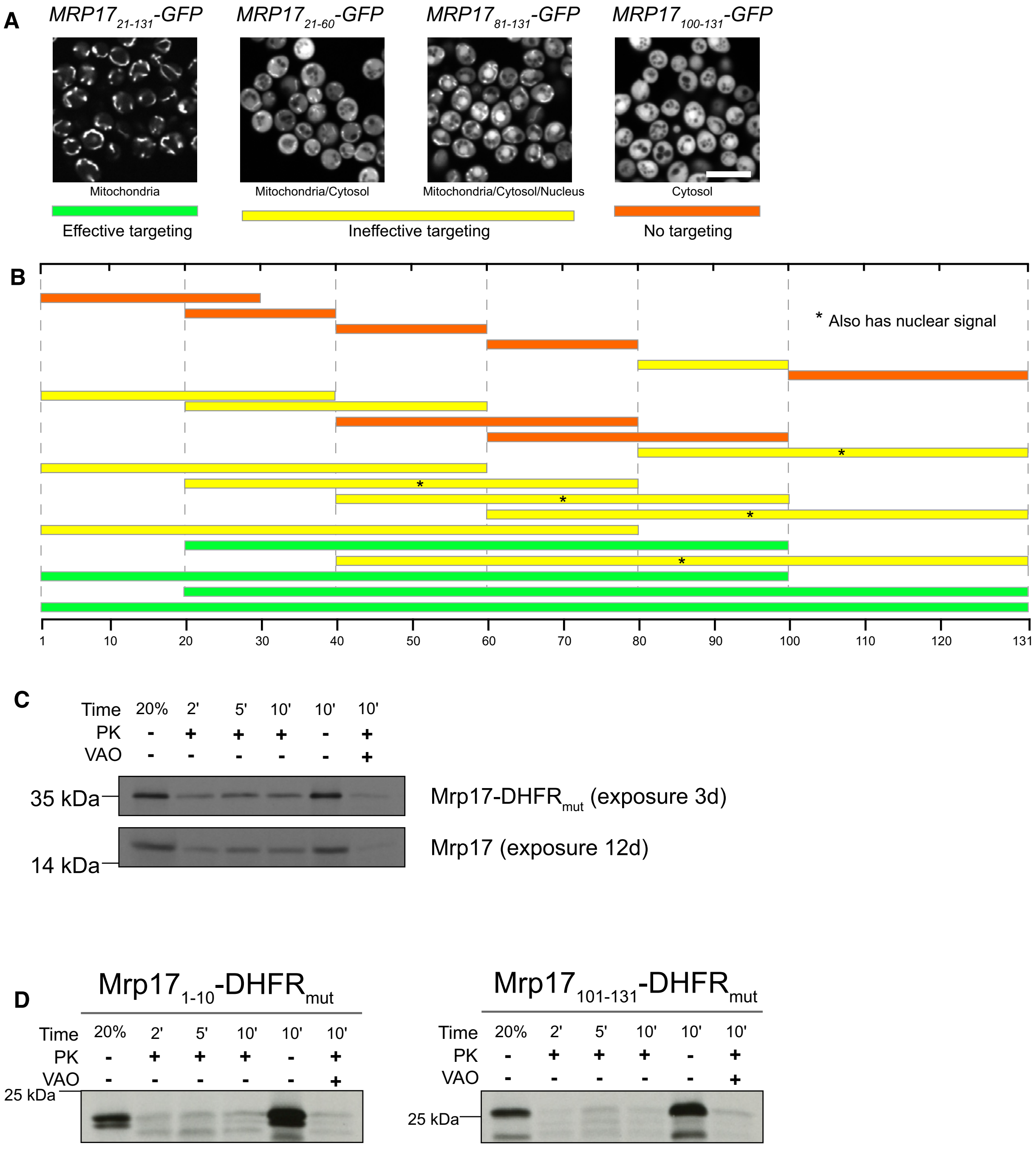

Figure EV3. The noncanonical targeting and translocation signal of Mrp17 is located between amino acids 30 and 60.

A, B *In vivo* characterization of mitochondrial targeting capacity of different Mrp17 truncations fused to GFP: (A) GFP localization examples for truncations, and demonstrating effective targeting with only mitochondrial GFP signal (Mrp17$_{21–131}$-GFP, same data as in Appendix Fig S5); ineffective targeting with mitochondrial GFP signal accompanied by strong cytosolic signal (Mrp17$_{21–60}$-GFP, same data as in Fig 2A and Appendix Fig S5); ineffective targeting with additional nuclear signal (Mrp17$_{81–131}$-GFP, same data as in Appendix Fig S5); and no detectable mitochondrial targeting with exclusively cytosolic GFP (Mrp17$_{100–131}$-GFP, same data as in Appendix Fig S5). Scale bar is 10 μm. (B) localization summary of different Mrp17 truncations fused to GFP and colored according to the color-code for effective, ineffective, and no targeting introduced in panel (A), truncations additionally targeted to the nucleus are marked with asterisks.

C Mrp17-DHFR$_{mut}$ is translocated into isolated mitochondria at the same rate as WT Mrp17 but gives better signal in the autoradiograph.

D *In vitro* import assays for additional truncations of Mrp17 fused to DHFR$_{mut}$ not shown in Fig 2B, import was performed as described in the legend for Fig 2.

© 2021 The Authors

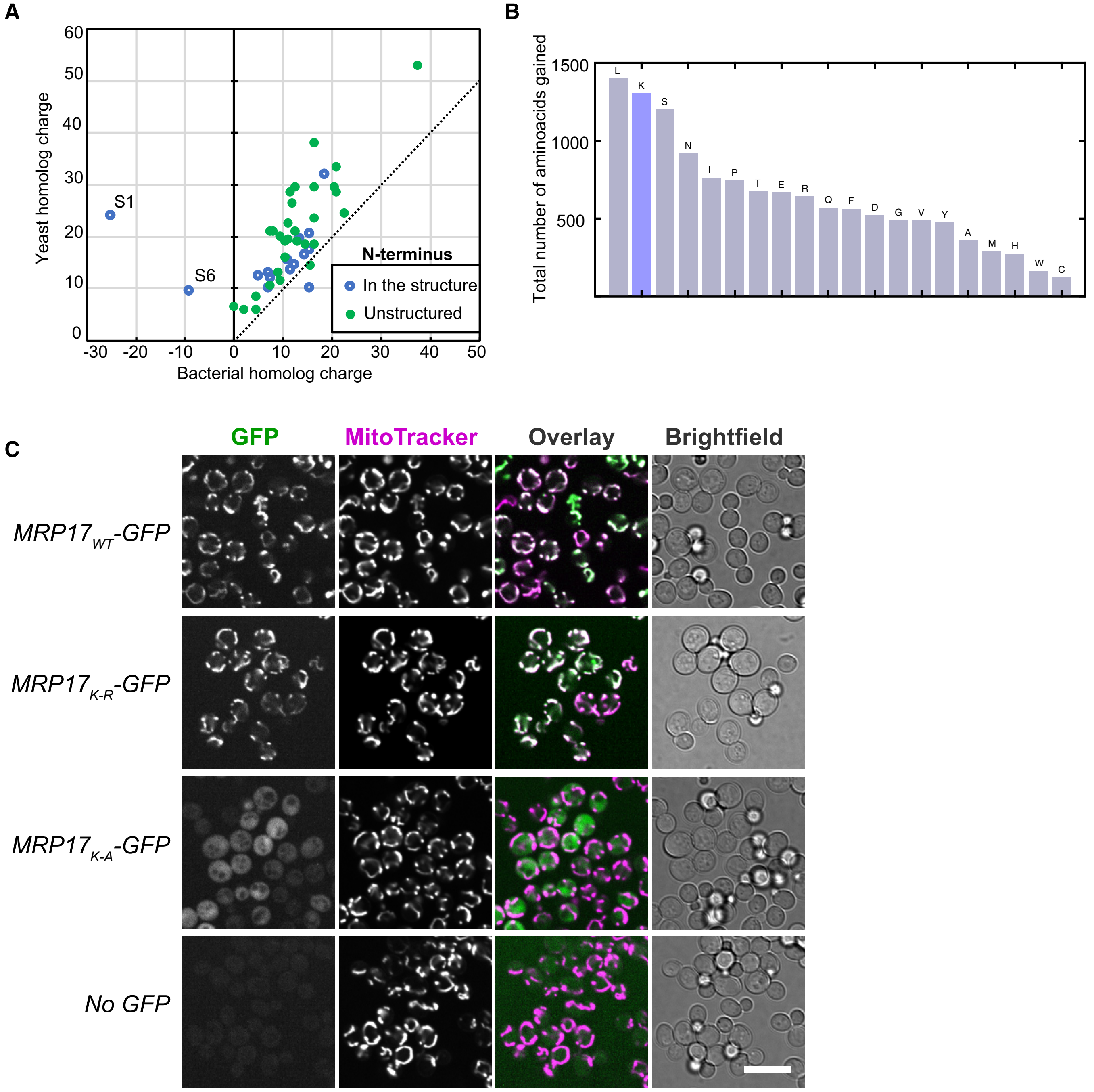

Figure EV4. Mrp17 sequence features important for targeting and translocation to mitochondria.

A Ribosomal proteins are positively charged and mitochondrial proteins acquired even more positive net charge compared with their bacterial homologs.

B Total amino acid gain of MRPs (calculated as the difference between total count of each amino acid in all yeast MRPs, including mitochondria-specific, and all bacterial RPs) compared with bacterial RPs shows over-representation of lysines (K).

C Lysines in Mrp17 are not important for mitochondrial targeting and can be substituted with arginines, same micrographs for constructs MRP17$_{WT}$-GFP, MRP17$_{K\text{-}R}$-GFP, and MRP17$_{K\text{-}A}$-GFP as in Fig 3C shown in all channels beside micrographs of yeast not expressing any GFP (bottom row) as control for autofluorescence relative to cytosolic signal. All micrographs in the GFP channel are shown at the same contrast and brightness for comparison; Scale bar is 10 µm.

© 2021 The Authors

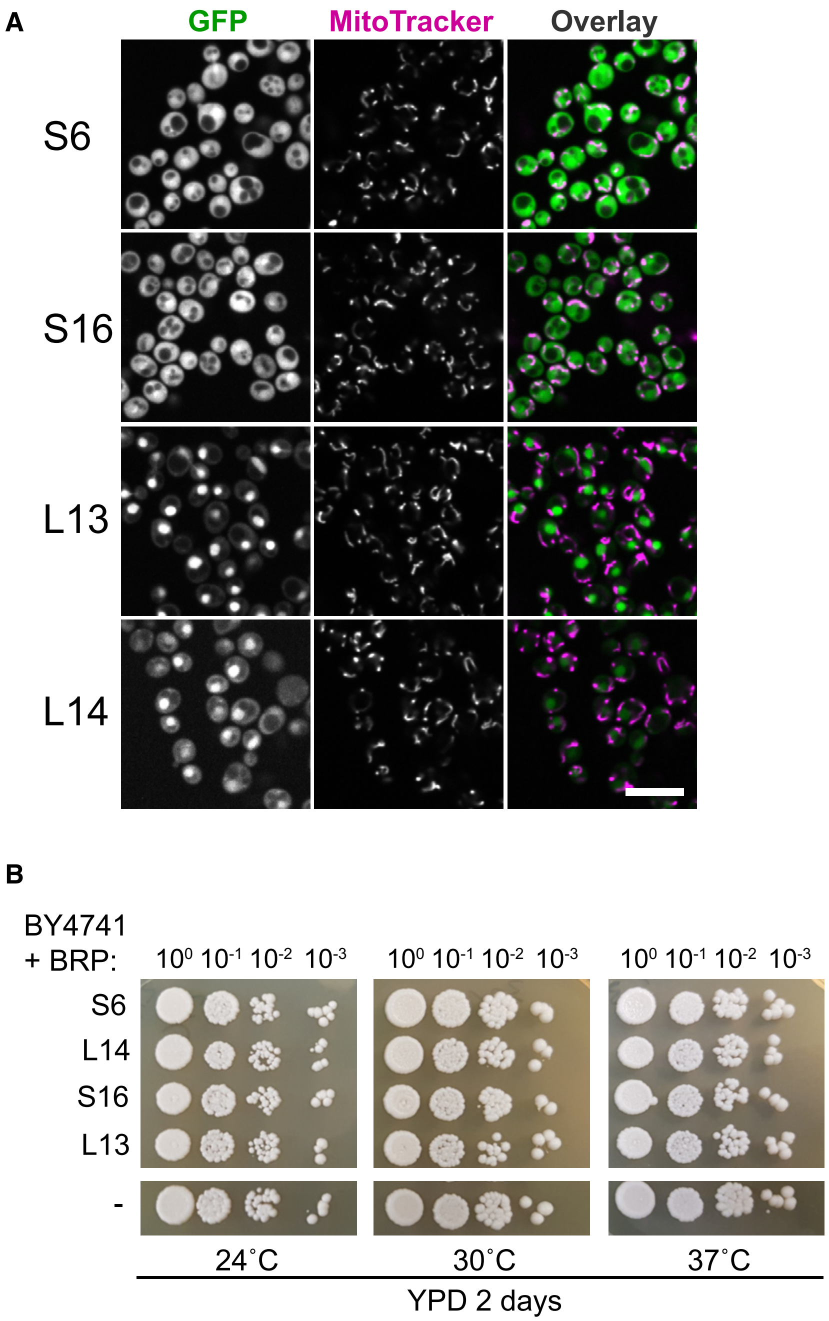

Figure EV5. Comparison of Mrp17 and its bacterial homolog.

A Expression of bacterial homologs of MRPs in yeast, same micrographs as in Fig 5C shown in all channels.

B Drop dilution growth assay for all the strains from panel A and WT control performed on rich fermentative media at different temperatures.

© 2021 The Authors
