# Supplementary figures and images for: Widespread use of unconventional targeting signals in mitochondrial ribosome proteins
Source: EMBO J. 2021 Nov 17;41(1):e109519. doi: 10.15252/embj.2021109519 (PMC8724765; doi:10.15252/embj.2021109519)

Source data for Fig. 4F

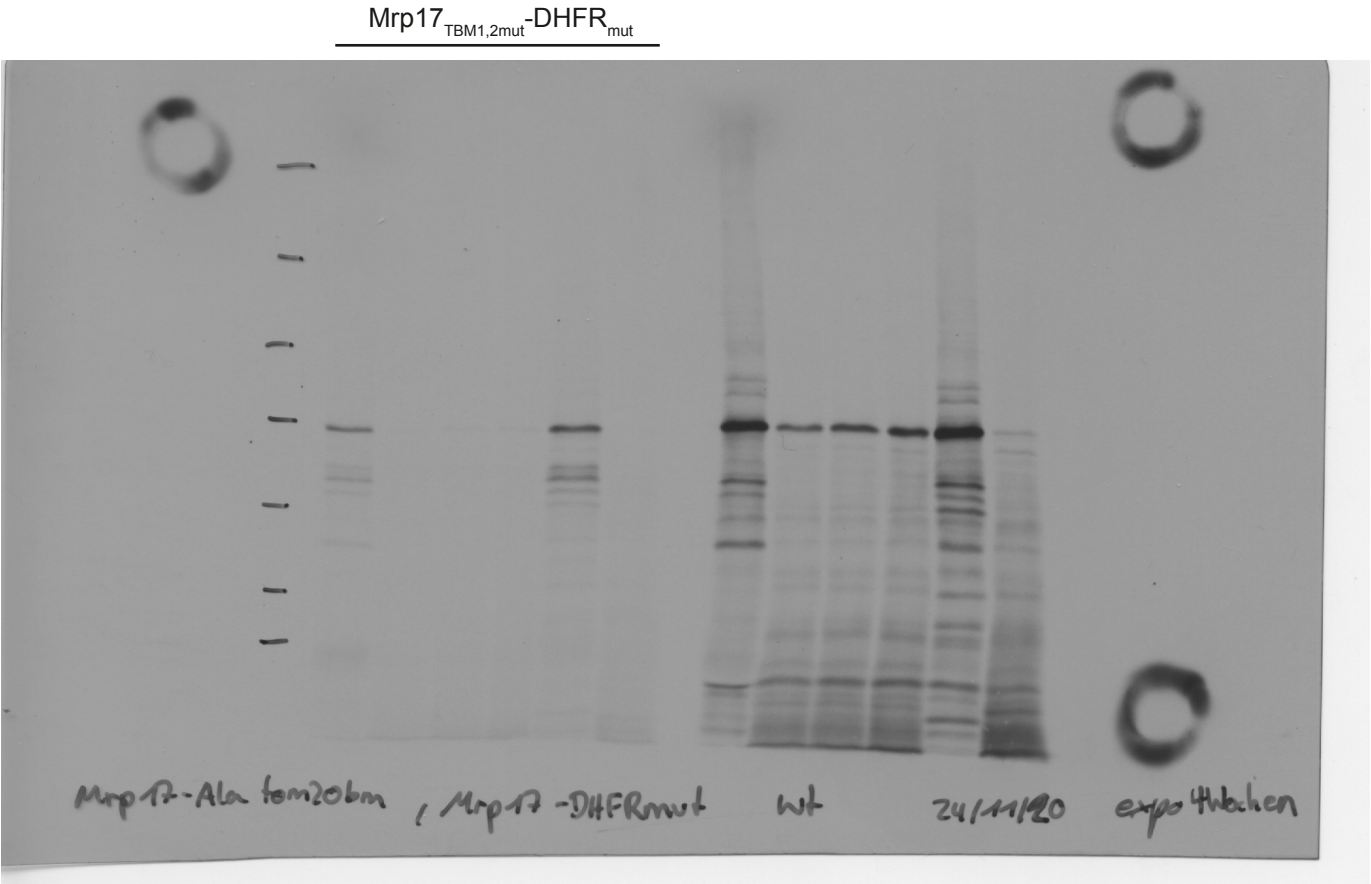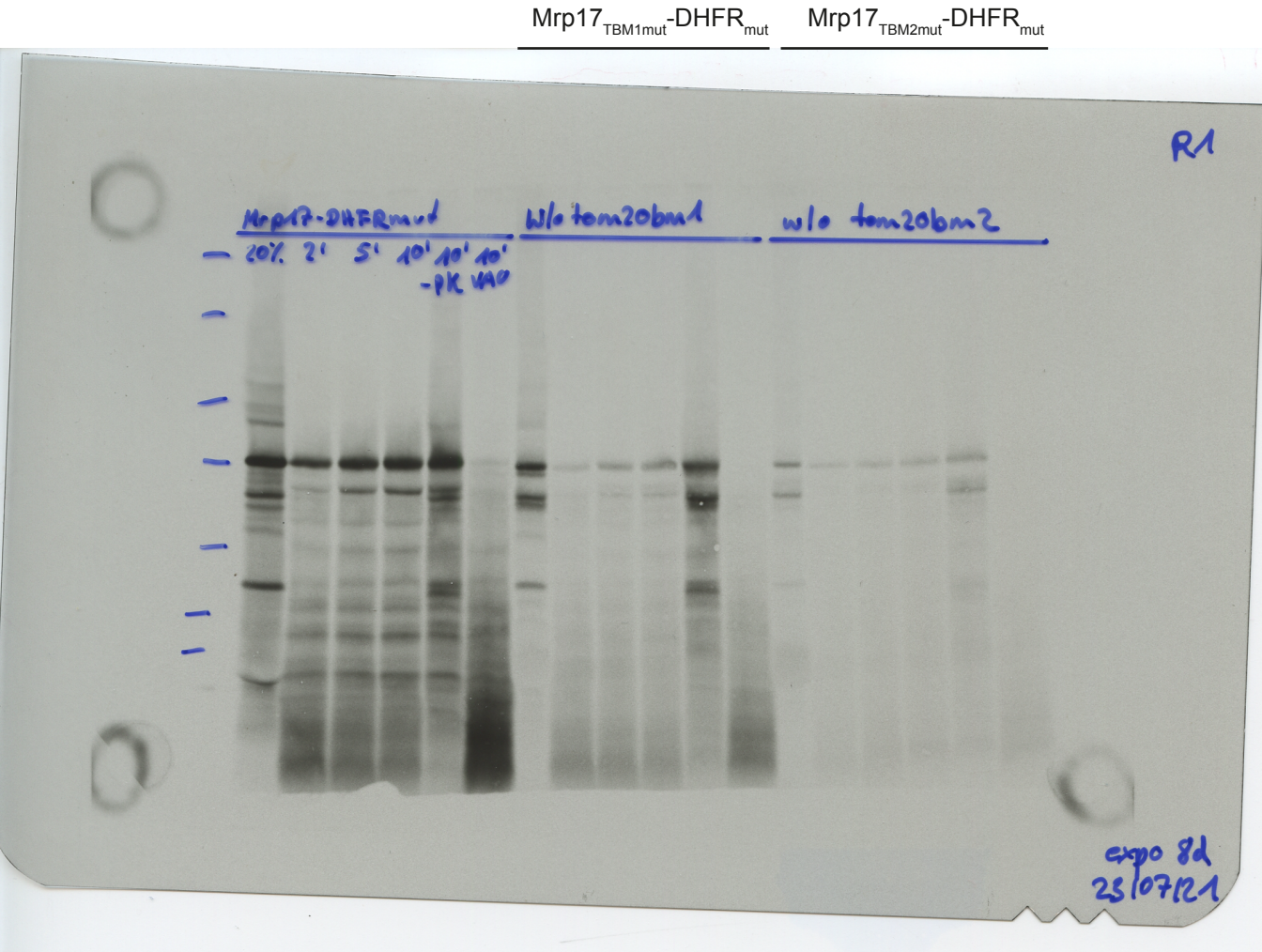

Supplement: Supplementary file 5 — Source Data for Figure 4 [file EMBJ-41-e109519-s006.pdf]
