# Supplementary material for: Widespread use of unconventional targeting signals in mitochondrial ribosome proteins
Source: EMBO J. 2021 Nov 17;41(1):e109519. doi: 10.15252/embj.2021109519 (PMC8724765; doi:10.15252/embj.2021109519)
Supplement: Supplementary file 6 [file EMBJ-41-e109519-s002.pdf]

THE EMBO JOURNAL

Widespread use of unconventional targeting signals in mitochondrial ribosome proteins

Yury Bykov, Tamara Flohr, Felix Boos, Naama Zung, Johannes Herrmann, and Maya Schuldiner
DOI: 10.15252/embj.2021109519

Corresponding author(s): Maya Schuldiner (maya.schuldiner@weizmann.ac.il) , Johannes Herrmann (hannes.herrmann@biologie.uni-kl.de)

Review Timeline:

Transfer from Review Commons:	22nd Aug 21
Editorial Decision:	21st Sep 21
Revision Received:	4th Oct 21
Accepted:	11th Oct 21

Editor: Stefanie Boehm

Transaction Report: This manuscript was transferred to The EMBO Journal following peer review at Review Commons.

Review COMMONS

(Note: With the exception of the correction of typographical or spelling errors that could be a source of ambiguity, letters and reports are not edited. Depending on transfer agreements, referee reports obtained elsewhere may or may not be included in this compilation. Referee reports are anonymous unless the Referee chooses to sign their reports.)

Review #1 -

Bykov, Flohr et al. interrogated unconventional mitochondrial targeting signals present in mitochondrial ribosomal proteins. Perplexingly, approximately a quarter of these exclusively mitochondrial proteins do not contain clear mitochondria targeting sequences, therefore, the guiding principles directing their localization and subsequent import and assembly into a functional ribosome have remained unknown. They find a subset of MRPs contain internal sequences that function as targeting signals instead. Focusing on Mrp17, they identify internal sequences both necessary and sufficient for mitochondrial targeting. They find these sequences are not an intrinsic property of the ancient bacterial protein but rather acquired during evolution. Additionally, they identify residues required for interaction with Tom20 and import into a mitochondrion. Lastly, they discuss the evolutionary constraints that may have prevented the addition of N-terminal targeting sequences.

Major comments:

The data is of high quality and is presented in a clear and convincing manner. None the less, there are a few areas that I think the authors can address to rule out several alternative hypotheses, further support their conclusions, and in some cases increase the impact.
1) Is it proper to equate localization and mitochondrial import, or should those be considered as two independent steps: one that perhaps brings the RNA, nascent chain, or complete protein to the mitochondrion and another method that permits the protein to be imported?

2) Can the authors rule out that protein-protein interactions- either with other ribosomal subunits or with Tom20 -- are not required for Mrp17 localization. That is, could co-translational assembly of multiple ribosomal proteins partially inform Mrp17 localization? Along these lines, does the deletion of Tom20 disrupt the localization and/or import of Mrp17-GFP?

3) How common are the appearance of the patches of positively charged residues across other MRPs with internal sequences identified by the authors?

4) As the authors point out, the structure of many species ribosomal subunits have now been published from cryo-EM data. There is little discussion of how well either the structure (and hence evolutionary constraints) and identified targeting sequences are conserved in other eukaryotes. The authors brilliantly use expression of the bacterial homolog to show the essential, but I am curious if the homologues protein from an evolutionarily distant yeast, or human homolog behave similarly? This would certainly support the author's conclusion of the internal evolutionary requirement of these sequences and would expand the significance of the manuscript.

5) Can the authors show that an N-terminal addition to Mrp17 would be impossible to accommodate? Can mislocalization of Mrp17K-A-GFP be rescued by fusing with an N-terminal targeting motif, but will this fusion construct not rescue the fitness of MRP17 deficient strains. Alternatively, a GFP-Mrp17 fusion would be expected to also not complement MRP17 null cells.

6) The title: it seems rather than structural constraints it is functional constraints that preclude the addition of the MTS. Additionally as this manuscript revolves around characterization of

Mrp17, I feel the title should better reflect this.

Minor comments

1) In text reference callout appear in alphabetical order rather than publication date order.

2) Line 22: Unclear if this refers to the yeast proteome only or if this true across organisms.

3) Paragraph from line 60-66, particularly line 60 and 63 is confusingly phrased when describing the contents of ribosomal genome in eukaryotes and "animals". Perhaps refer to Kingdom animalia?

4) Line 94-95: I'm not sure the authors characterize the role in assembly of a complete ribosome complex/subunit.

5) Figure 4 panel E: The blot on the right is missing a molecular weight label.

6) Figure 4 figure legend. I think TOM40/TIM23 are referring to proteins Tom40, Tim23.

7) Figure 6 panel B: It is unclear what this panel is trying to show.

8) Figure 6 panel D: Which statistical test was used and what p value does " ** " represent?

9) Figure S2: I think it would be helpful to annotate those proteins that were selected for analysis.

10) Figure S4 B: It would be useful to have a nuclear marker in these images. Are these puncta associated with the nucleolus?

11) Figure S10: Several of the labels on the left overlap with the cartoon which makes them difficult to read.

12) Are these internal targeting sequences exclusive to MRPs or could other mitochondrial proteins be targeted via similar mechanisms?

Many mitochondria ribosomal proteins (MRPs) do not contain N-terminal mitochondrial target sequences. How they are able to correctly localize to mitochondria has remained largely unknown. Verifying that many MRPs can target to mitochondria in the absence of their N-terminal domains and subsequently identifying the properties of internal sequences required for the localization of one representative MRP (Mrp17), is an interesting advance to the understating of mitochondria protein localization and import. I find this work to be of broad interest to cell, structural and evolutionary biologists. The work is novel, and the data is of excellent quality.

Review #2 -

Summary

The study by Bykov et al shows that some mitoribosomal proteins (MRPs) have unconventional internal targeting signals. Specifically, the study analyzed available data on N-terminal mitochondrial targeting signals (MTSs) of yeast MRPs and confirmed that up to 25% of MRPs do not have a predictable conventional MTS. A structure-function analysis of 15 MRPs determined 3 principal modes of "unconventional" mitochondrial targeting: MRPs with an 30 amino acid long N-terminal MTS that typically exhibit low MTS prediction scores; MRPs for which the targeting information is either distributed over the entire protein or contained in an N-terminal segment longer than 30 amino acids; and MRPs with internal targeting signals after the first 30 N-terminal amino acids. Of the latter group, one MRP, Mrp17 (bS6), was further analyzed because it is one of the most conserved proteins across all mitoribosomal structures but lacks any predictable targeting signal. Further analysis of Mrp17 showed that it's main mitochondrial targeting signal is located between amino acids 30 to 60 while additional unidentified signals in the C-terminal half improve targeting efficiency or stability. Finally, the studies indicates that two TOM20-binding motifs and a set of positive charges in the internal targeting sequence are critical for mitochondrial targeting and import. Accordingly, Mrp17's import pathway is similar to proteins containing conventional MTSs.

Major comments

The study is interesting but unfortunately somewhat preliminary, which limits its impact. I have a number of comments.

1) The study suggests that Mrp17's internal targeting sequence is a bona fide MTS, even though prediction algorithms do not recognize it. While this is an interesting result, the study falls short determining any additional new features that make this sequence an internal MTS next to the already known features like positive charges and TOM20-binding motifs. A more detailed analysis of Mrp17's internal targeting sequence should reveal additional features that are currently not considered by MTS prediction algorithms.

2) The study suggests that lysine-based positive charges are required for Mrp17's mitochondrial targeting.
However, I am confused whether all lysines of Mrp17 were mutated or only the lysines within residues 30-60. Please, clarify this issue.

If indeed all lysines were mutated, it is critical to differentiate between lysine-based positive charges in the identified internal targeting sequence (residues 30-60) and the positive charges outside this domain, which might not be required at all.

3) The study suggests that Mrp17's internal targeting sequence shares critical features with conventional MTSs and mitochondrial import mechanisms. However, the current analysis may be misleading since the used MRP17-K-A and K-R mutants are potentially double mutants affecting both the first TOM20 binding domain and all of the positive charges in the internal targeting domain or the entire protein (see above). Therefore, the authors need to test a K-A mutant that substitutes all lysine residues except the one in the first TOM20 binding motif.

3) The study used a double mutant affecting both TOM20-binding motifs. However, it remains unclear whether only one of these motifs or both are required for mitochondrial targeting/import. Therefore, mutants affecting only individual TOM20-binding motifs should be tested.

4) The study suggests that the residual translocation of Mrp17-DHFR in Δtom20 mutants may be facilitated
by TOM22. This should be and can be easily tested.

5) The study identified a number of MRPs containing internal mitochondrial targeting sequences and further characterized one of them, the internal targeting sequence of Mrp17. An obvious question is whether the internal targeting sequence of Mrp17 is unique to MRP17, or whether it is more broadly used, especially in PET123, MRPL23, MRP35, or MRP20?

The conceptual significance of the study is limited for a number of reasons. First, it is already known that many MRPs do not have a predictable conventional N-terminal MTS. Second, the study fails to identify any novel motifs that make Mrp17's internal targeting sequence functional and/or explain its internal nature. Third, it remains unclear whether Mrp17's internal targeting sequence is unique for Mrp17, or has a broader application.

22nd Aug 2021

Authors' submitted point-by-point response to Review Commons reports.

Reviewer 1

1) Is it proper to equate localization and mitochondrial import, or should those be considered as two independent steps: one that perhaps brings the RNA, nascent chain, or complete protein to the mitochondrion and another method that permits the protein to be imported?

We agree with the reviewer that targeting and translocation shouldn't be equated. In our manuscript we separate these two processes conceptually by defining them in the Introduction (lines 68-73).

However, experimental separation of targeting and translocation is more challenging. Here we tried to do it by using two complimentary techniques – fluorescent microscopy of living cells and import into isolated mitochondria. Fluorescent microscopy provides information on targeting together with translocation but conceals if translocation actually happened or GFP is just located on mitochondrial surface. Also, *in vivo* assay is hurdled by instability of truncated proteins both in the cytosol when they are not targeted and in mitochondria after targeting which complicates assay interpretation. *In vitro* import assay allows to study just translocation step while the targeting steps are absent due to the absence of native yeast cytosol and other organelles in the assay (rabbit reticulocyte lysate is used).

To make sure that this is clear to our readers we have now changed the text on lines 200-203 to read like this:

> The microscopic analysis does not allow us to discriminate between targeting to the mitochondrial surface from complete translocation into the matrix as well is strongly affected by truncated MRPs stability *in vivo*. To elucidate the translocation efficiency of different Mrp17 regions we used in vitro import assays into isolated yeast mitochondria.

Having said that - thanks to the points raised by the reviewers - we have actually now partially addressed this point experimentally by dissecting the role of charged residues and Tom20-binding motifs (TBM) in Mrp17 import in the newly added experiments. In particular, we confirm the requirement of both TBMs for the import by testing individual mutants of these motifs (newly added data in Fig. 4F). We also showed previously that they do not work without positive charges by testing the import of alanine mutant Mrp17K-A where all lysines were substituted with arginines, but now we also show that the lysines play no other specific function in respiration and their function can be complemented by adding a strong presequence (newly added Fig. 3E). Finally, we confirm the importance of positive charges by adding a new chimeric construct of Mrp17 and EcS6 that has more positive charges and is able to be translocated into mitochondria with better efficiency (newly added Fig. 5E). Based on this, we propose a model for Mrp17 targeting and translocation where different import steps are mediated by different sequence features (TBMs and charge) in newly added Fig. 5F.

2) Can the authors rule out that protein-protein interactions- either with other ribosomal subunits or with Tom20 -- are not required for Mrp17 localization. That is, could co-translational assembly of multiple ribosomal proteins partially inform Mrp17 localization? Along these lines, does the deletion of Tom20 disrupt the localization and/or import of Mrp17-GFP?

Co-import (or piggy back import) of protein complexes is known for protein translocation into peroxisomes or the nucleus, where folded protein and complexes are translocated. Since mitochondrial import requires unfolded polypeptide chains, this hypothesis seems less likely and was never described for any other protein before. However, we think we can rule this out completely due to the high consistency between our *in vitro* import experiments and our *in vivo* data with GFP fusions since in the *in vitro* assays only the tested proteins were added.

Following the suggestion to test Mrp17-GFP localization in Δ*tom20* cells, we have now performed this experiment (newly added Fig. 4D) and found that loss of *TOM20* results in the mislocalization of Mrp17-GFP to the nucleus, in line with our conclusions from the *in vitro* import experiments. This newly added experiment nicely confirms the crucial nature of Tom20 (and the Tom20-binding sites) for Mrp17 targeting.

3) How common are the appearance of the patches of positively charged residues across other MRPs with internal sequences identified by the authors?

There is no significant difference in the content of positively charged amino acids (arginine+lysine) between MTS-containing MRPs (analyzed separately for MTS and mature part), MRPs without MTS and cytosolic ribosomal proteins (control). See figure below. Since the manuscript is already very data-heavy we suggest not to put this data into the manuscript.

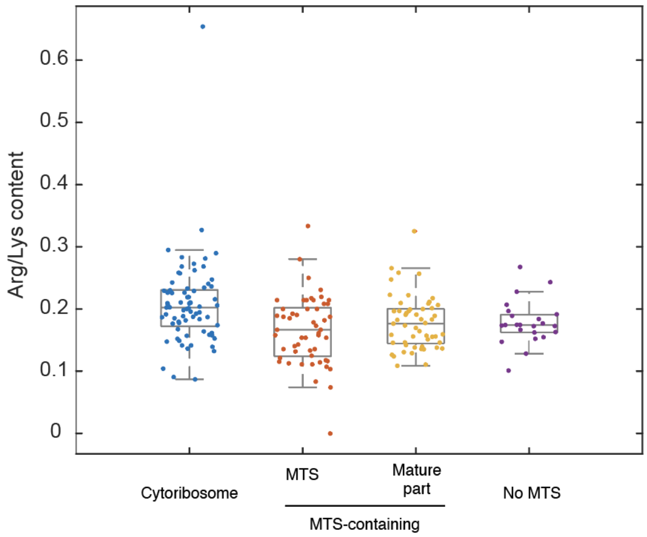

However, if only the MRP portions acquired over the course of evolution in addition to bacterial proteins are analyzed, lysines are one of the most popular newly acquired amino acids (Fig. S8B). Based on newly added experiment, where we show that Mrp17 lysines are mainly important for protein import but not mitoribosome function (newly added Fig. 3E), we suggest that positive charges played an important role in the evolution of targeting signals in MRPs.

4) As the authors point out, the structure of many species ribosomal subunits have now been published from cryo-EM data. There is little discussion of how well either the structure (and hence evolutionary constraints) and identified targeting sequences are conserved in other eukaryotes. The authors brilliantly use expression of the bacterial homolog to show the essential, but I am curious if the homologues protein from an evolutionarily distant yeast, or human homolog behave similarly? This would certainly support the author's conclusion of the internal evolutionary requirement of these sequences and would expand the significance of the manuscript.

To address this important point, we studied Mrp17 homologs from the fungus, *Neurospora crassa,* and from humans in more detail. The fungal homolog was imported into isolated yeast mitochondria, while the human homolog was not (please see figure below).

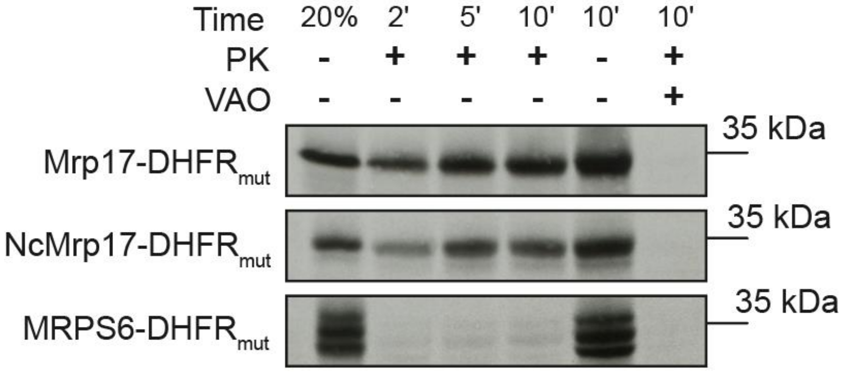

Thus, the use of internal signal is a conserved feature but signal properties recognized by the import machinery can differ between remote relatives like yeast and humans. The nature of the signal in human MRPS6 requires further study that cannot be done in the yeast system, so we considered this data to be out of scope of this paper and did not include it in the revised manuscript.

To further emphasize conservation of the N-terminal interface of Mrp17 homologs with mitoribosomes we added snapshots of *N. crassa* and human mitoribosome structures to Fig. S12A.

5) Can the authors show that an N-terminal addition to Mrp17 would be impossible to accommodate? Can mislocalization of Mrp17K-A-GFP be rescued by fusing with an N-terminal targeting motif, but will this fusion construct not rescue the fitness of MRP17 deficient strains. Alternatively, a GFP-Mrp17 fusion would be expected to also not complement MRP17 null cells.

We thank the referee for this excellent suggestion. We now performed functional complementation experiments in which we replaced the endogenous Mrp17 protein by versions in which Mrp17 is imported via the strong presequence of *N. crassa* ATP-synthase subunit 9 (Su9). We constructed the protein in a manner which resulted in an almost unaltered N-terminus of the MPP-cleaved, mature, Mrp17 protein. Interestingly, this version <u>was</u> able to replace Mrp17. We were even more surprised to see that the presequence also conferred the import of the alanine mutant which even resulted in the complementation of the *MRP17* deletion strain. This nicely confirms that the addition of lysines is caused by their role in protein import rather than in their necessity for ribosome function. This newly added experiment is now shown in Fig 3E.

We also constructed an N-terminal fusion of Mrp17 with GFP in its native genomic location. This yeast strain lost the capacity to grow on respiratory media and had a significant growth defect on fermentative media (see figure below). The GFP signal was detected in the nucleus but not in mitochondria, suggesting that such mutant is not even imported into mitochondria and thus unable to answer the question whether the N-terminal extension disturbs Mrp17 function. It was also unclear whether the mitochondrial import was inhibited due to the presence of a folded domain (GFP) or because the targeting signal of Mrp17 needs to be within a certain distance of the N-terminus. Due to this ambiguity we did not include this data in the revised manuscript. See figure below: (top) growth assay of WT yeast and GFP-Mrp17 yeast on respiratory and fermentative media, (bottom) fluorescent microscopy of GFP-Mrp17 strain (scale bar 10 µm).

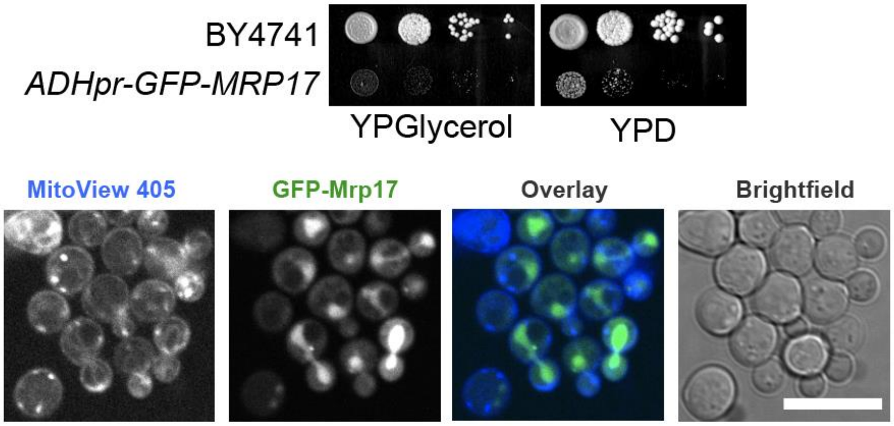

6) The title: it seems rather than structural constraints it is functional constraints that preclude the addition of the MTS. Additionally as this manuscript revolves around characterization of Mrp17, I feel the title should better reflect this.

About a third of all MRPs have unconventional targeting sequences, and we show many examples in the study. We use Mrp17 as specific case to unravel its import properties in detail. Still, our manuscript sheds light on the targeting and import of MRPs in general. Therefore, we feel that our general title reflects better the content of our study and would prefer not to change it. However, we leave this up to editorial decision.

We also think that the experiment with adding a strong synthetic MTS to Mrp17 (newly added Fig. 3E) suggests that it's rather structural than functional constraints precluded the evolution of an N-terminal MTS.

Minor comments

1) In text reference callout appear in alphabetical order rather than publication date order.

This is now corrected in the revised version.

2) Line 22: Unclear if this refers to the yeast proteome only or if this true across organisms.

This particular estimate of 25% was made for yeast, so we added this clarification to the abstract.

3) Paragraph from line 60-66, particularly line 60 and 63 is confusingly phrased when describing the contents of ribosomal genome in eukaryotes and "animals". Perhaps refer to Kingdom animalia?

We meant multicellular animals and now refer to this group as "Metazoa" which is less ambiguous.

4) Line 94-95: I'm not sure the authors characterize the role in assembly of a complete ribosome complex/subunit.

This notion was now removed and this sentence now reads (Lines 102-103):

> In this work, we studied the mechanisms by which MRPs are targeted and translocated into mitochondria.

5) Figure 4 panel E: The blot on the right is missing a molecular weight label.

To make the figure look consistent we cropped all the gel scans to the same size and there was no molecular weight marker close enough to the band. To address this disadvantage, we added the full scan of this gel with all molecular weight markers to figure S9E and a reference to it into the Fig. 4 figure legend. We hope that this is an acceptable solution.

6) Figure 4 figure legend. I think TOM40/TIM23 are referring to proteins Tom40, Tim23.

We thank the reviewer for catching this mistake and have now changed labels in the figure and figure legends so that they refer to particular yeast proteins Tom40, and Tim17/23.

7) Figure 6 panel B: It is unclear what this panel is trying to show.

This panel is a legend that explains different MRP classes that we analyzed in Fig. 6. To make this clearer, we have added titles to the graphs and to this panel, moved the panel below the graphs and updated the figure legend and the manuscript text. We hope that this now is easier to understand.

*8) Figure 6 panel D: Which statistical test was used and what p value does " ** " represent?*

This was Mann-Whitney test and ** represented $p<0.01$. This information was added to Fig. 6 legend.

9) Figure S2: I think it would be helpful to annotate those proteins that were selected for analysis.

Proteins selected for analysis were marked with red rectangles in Fig. S2.

10) Figure S4 B: It would be useful to have a nuclear marker in these images. Are these puncta associated with the nucleolus?

To address this point, we have performed microscopy of 6 strains transformed with a nuclear marker NLS-tdTomato and of 4 strains with Nop2-mCherry, a nucleolar marker. This data is added to Fig. S4 as panels B and C. The GFP puncta were positioned randomly relatively to the nucleoli visualized using Nop2-mCherry hence we do not believe that there is some preferred relative localization of puncta and nucleoli.

11) Figure S10: Several of the labels on the left overlap with the cartoon which makes them difficult to read.

This is corrected in the revised version.

12) Are these internal targeting sequences exclusive to MRPs or could other mitochondrial proteins be targeted via similar mechanisms?

Yes, to our knowledge, these targeting sequences are exclusive to MRPs. All other matrix proteins contain MTSs either at their N-termini or their very C-terminus (Hmi1, for reverse

import). However, there are other matrix proteins which have non-cleaved MTSs such as Hsp10. However, the MRPs are really unique as here, many have no sequences that would reveal the mitochondrial localization on basis of prediction algorithms. As shown by us now, this is not due to the fact that the algorithms are bad, but due to the very different nature of the targeting information that is located within the sequence.

It is also possible to make a candidate list of matrix proteins without cleavable targeting sequences from existing data, but there is a lot of uncertainty in it. We tried to do it the following way: take the N-terminal proteomics data (Vögtle et al. 2009), select proteins with uncleaved N-termini, then use proteomics data from (Vögtle et al. 2017) or (Morgenstern et al. 2017) to select only matrix proteins, then additionally remove proteins with trans-membrane domains (TMD) using TMD prediction software to get only soluble proteins. Then it is possible to use MTS prediction software to estimate if the targeting signal is at the N-terminus or it can be internal. Such final filtered list usually contains 30-50 proteins of which MRPs are always the dominant category. However, on closer examination it appears that many other proteins in the list are either membrane proteins with poorly predicted TMDs (Mir1, Sfc1), peroxisome proteins (Tes1), proteins with experimentally determined cleavable MTS (Hem15), or likely to be uncleavable but probably still have targeting signal at the N-terminus (Mtg1, Mrs1). The candidate list changes considerably depending on which proteomics dataset and TMD prediction algorithm is used. For these reasons we have decided not to put any of these estimates in our manuscript.

Reviewer 2

1) The study suggests that Mrp17's internal targeting sequence is a bona fide MTS, even though prediction algorithms do not recognize it. While this is an interesting result, the study falls short determining any additional new features that make this sequence an internal MTS next to the already known features like positive charges and TOM20-binding motifs. A more detailed analysis of Mrp17's internal targeting sequence should reveal additional features that are currently not considered by MTS prediction algorithms.

We are grateful to this reviewer for challenging us to add a more detailed analysis of the features that guide Mrp17 targeting. Indeed, we have now added several pieces of data that we believe address this point. We now show that the Mrp17 targeting signal requires a combination of Tom20-binding motifs with positive charges (both internal and flanking) and that these two signals can be dissected. Our new Fig. 3E demonstrates the particular importance of Mrp17 lysines for import, but not mitoribosome function. New Fig. 4F dissects the relative importance of Tom20-binding motifs. Finally, new S6/Mrp17 chimeric constructs (new Fig. 5E) emphasize the importance of charged residues. We also partially address this issue by computational analysis, see our reply to your point 6.

We understand the reviewer’s concern that our model of the Mrp17 targeting signal cannot be easily formalized and used on other proteins to predict targeting. However, defining the conserved features that serve a broader range of proteins depends on there being such a

singular feature (which is not a necessity) as well as significant experimental effort on multiple additional MTS-less proteins, similar to those applied over the years to the studies of regular MTS. Such extensive experiments are beyond the scope of this work.

2) The study suggests that lysine-based positive charges are required for Mrp17's mitochondrial targeting. However, I am confused whether all lysines of Mrp17 were mutated or only the lysines within residues 30-60. Please, clarify this issue.

If indeed all lysines were mutated, it is critical to differentiate between lysine-based positive charges in the identified internal targeting sequence (residues 30-60) and the positive charges outside this domain, which might not be required at all.

We agree with the reviewer that this experiment was not clearly described. We added a schematic to Fig. 3D that explains that we did the mutant mentioned by the reviewer.

3) The study suggests that Mrp17's internal targeting sequence shares critical features with conventional MTSs and mitochondrial import mechanisms. However, the current analysis may be misleading since the used MRP17-K-A and K-R mutants are potentially double mutants affecting both the first TOM20 binding domain and all of the positive charges in the internal targeting domain or the entire protein (see above). Therefore, the authors need to test a K-A mutant that substitutes all lysine residues except the one in the first TOM20 binding motif.

To address this point, we tested such a mutant where all lysines were mutated to alanines except the lysine in the first Tom20-binding motif. This mutant was not imported into isolated mitochondria suggested that many positive charges around the Tom20-binding motif are also required for translocation (see figure below).

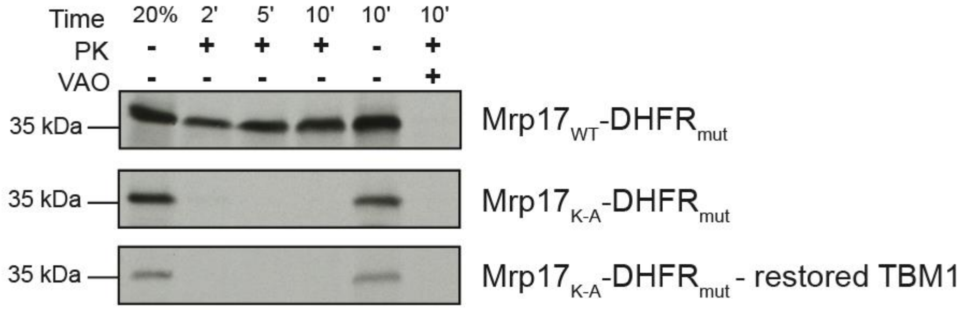

4) The study used a double mutant affecting both TOM20-binding motifs. However, it remains unclear whether only one of these motifs or both are required for mitochondrial targeting/import. Therefore, mutants affecting only individual TOM20-binding motifs should be tested.

To address this point, we constructed two mutants that had either motif 1 or motif 2 substituted with alanines. We found that both mutants had some import capacity compared to the double mutant concluding that both motifs are required for efficient import. We have added this data to Fig. 4, panel F. We wish to thank the referee for this suggestion! This new experiment improved our understanding because it showed that both TBS are necessary and that they cooperate in the targeting of Mrp17 to the mitochondrial outer membrane. The positive charges throughout the sequence further confer translocation through the TOM and TIM23 channels.

5) The study suggests that the residual translocation of Mrp17-DHFR in Δtom20 mutants may be facilitated by TOM22. This should be and can be easily tested.

We tested this possibility by performing in Mrp17-$DHFR_{mut}$ import into mitochondria isolated from Δ*tom22* strain. The import in these mitochondria was abolished. We added this data as Fig. 4E.

6) The study identified a number of MRPs containing internal mitochondrial targeting sequences and further characterized one of them, the internal targeting sequence of Mrp17. An obvious question is whether the internal targeting sequence of Mrp17 is unique to MRP17, or whether it is more broadly used, especially in PET123, MRPL23, MRP35, or MRP20?

To answer this question, the unique properties of Pet123, Mrpl23, Mrp35 and Mrp20 targeting signals should be investigated experimentally in a more detailed way which is outside the scope of this current paper.

However, to get some idea regarding this question we analyzed the presence of positively charged amino acids (see reply to point 3 raised by Reviewer #1) and Tom20-binding motifs (see figure below) in MRPs with and without MTS. We could find no difference between MRPs without MTS and other analyzed proteins. Indeed, in Mrp17 itself there are additional Tom20-binding motifs that are positioned beyond the targeting signal (aa 30-60) and seem irrelevant for targeting. Hence we decided not to include this data in the current, already data heavy, manuscript. Computational identification of the relevant Tom20-binding motifs for internal targeting sequences can be a subject for further research.

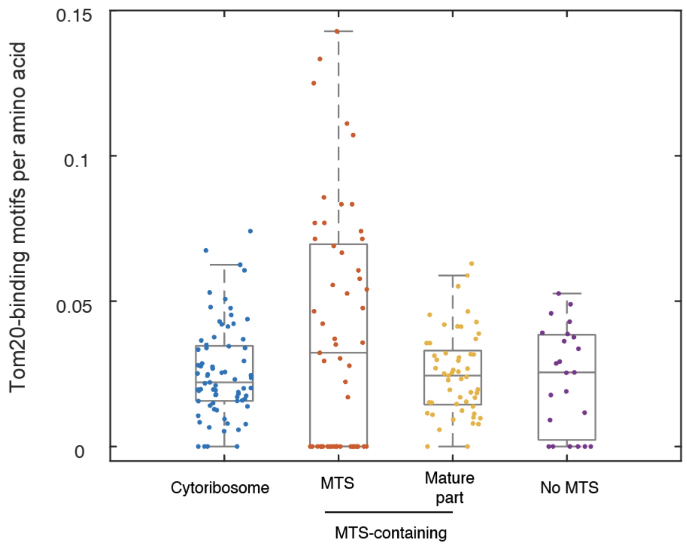

We also looked for other linear motifs using the MEME suite (https://meme-suite.org/meme/tools/meme) to ensure that we are not missing an interesting targeting motif. We compared MRPs with MTS to those without an MTS, and to cytosolic ribosomal proteins, but could not find any motifs in the Mrp17 targeting signal that are also found in other proteins.

Thank you for submitting your manuscript, which had been reviewed at Review Commons, to The EMBO Journal. We sent this revised version back to the initial referees and have now received their reports (please see comments below). I am pleased to say that they overall find that their concerns have been satisfactorily addressed and support publication of the revised version in The EMBO Journal. Therefore, I would ask you to now address a number of editorial and formatting issues that are listed in detail below. Once these issues are resolved, we will be happy to formally accept the manuscript for publication.

Thank you again for giving us the chance to consider your manuscript at The EMBO Journal. I look forward to receiving your final revision. Please feel free to contact me if you have further questions regarding the revision or any of the specific points listed below.

REFEREE REPORTS

Referee #1:

I remain enthusiastic about the general interest and significance of the manuscript. The authors have done really everything that was necessary to make the conclusions more robust. The manuscript is in great shape now.

Referee #2:

The additional experiments provided by the authors significantly improved the study and adequately support the main conclusions. I have no further concerns.

The authors have made all requested editorial changes.

ACCEPTED **11th Oct 2021**

Dear Dr. Schuldiner,

Thank you again for submitting the final revised version of your manuscript. I am pleased to inform you that we have now accepted it for publication in The EMBO Journal. However, please remember that the dataset GSE172017 must be public at the time of publication.

EMBO PRESS

YOU MUST COMPLETE ALL CELLS WITH A PINK BACKGROUND

PLEASE NOTE THAT THIS CHECKLIST WILL BE PUBLISHED ALONGSIDE YOUR PAPER

Corresponding Author Name: Maya Schuldiner, Johannes M. Herrmann
Journal Submitted to: The EMBO Journal
Manuscript Number: EMBOJ-2021-109519

Reporting Checklist For Life Sciences Articles (Rev. June 2017)

This checklist is used to ensure good reporting standards and to improve the reproducibility of published results. These guidelines are consistent with the Principles and Guidelines for Reporting Preclinical Research issued by the NIH in 2014. Please follow the journal's authorship guidelines in preparing your manuscript.

A- Figures
1. Data
The data shown in figures should satisfy the following conditions:

- the data were obtained and processed according to the field's best practice and are presented to reflect the results of the experiments in an accurate and unbiased manner.
- figure panels include only data points, measurements or observations that can be compared to each other in a scientifically meaningful way.
- graphs include clearly labeled error bars for independent experiments and sample sizes. Unless justified, error bars should not be shown for technical replicates.
- if n< 5, the individual data points from each experiment should be plotted and any statistical test employed should be justified
- Source Data should be included to report the data underlying graphs. Please follow the guidelines set out in the author ship guidelines on Data Presentation.

2. Captions
Each figure caption should contain the following information, for each panel where they are relevant:

- a specification of the experimental system investigated (eg cell line, species name).
- the assay(s) and method(s) used to carry out the reported observations and measurements
- an explicit mention of the biological and chemical entity(ies) that are being measured.
- an explicit mention of the biological and chemical entity(ies) that are altered/varied/perturbed in a controlled manner.

- the exact sample size (n) for each experimental group/condition, given as a number, not a range;
- a description of the sample collection allowing the reader to understand whether the samples represent technical or biological replicates (including how many animals, litters, cultures, etc.).
- a statement of how many times the experiment shown was independently replicated in the laboratory.
- definitions of statistical methods and measures:
  - common tests, such as t-test (please specify whether paired vs. unpaired), simple χ2 tests, Wilcoxon and Mann-Whitney tests, can be unambiguously identified by name only, but more complex techniques should be described in the methods section;
  - are tests one-sided or two-sided?
  - are there adjustments for multiple comparisons?
  - exact statistical test results, e.g., P values = x but not P values < x;
  - definition of 'center values' as median or average;
  - definition of error bars as s.d. or s.e.m.

Any descriptions too long for the figure legend should be included in the methods section and/or with the source data.

In the pink boxes below, please ensure that the answers to the following questions are reported in the manuscript itself. Every question should be answered. If the question is not relevant to your research, please write NA (non applicable). We encourage you to include a specific subsection in the methods section for statistics, reagents, animal models and human subjects.

USEFUL LINKS FOR COMPLETING THIS FORM

http://www.antibodypedia.com
http://1degreebio.org
http://www.equator-network.org/reporting-guidelines/improving-bioscience-research-repor
http://grants.nih.gov/grants/olaw/olaw.htm
http://www.mrc.ac.uk/Ourresearch/Ethicsresearchguidance/Useofanimals/index.htm
http://ClinicalTrials.gov
http://www.consort-statement.org
http://www.consort-statement.org/checklists/view/32-consort/66-title
http://www.equator-network.org/reporting-guidelines/reporting-recommendations-for-tumo
http://datadryad.org
http://figshare.com
http://www.ncbi.nlm.nih.gov/gap
http://www.ebi.ac.uk/ega
http://biomodels.net/
http://biomodels.net/miriam/
http://jjj.biochem.sun.ac.za
https://osp.od.nih.gov/biosafety-biosecurity-and-emerging-biotechnology/
http://www.selectagents.gov/

B- Statistics and general methods

Please fill out these boxes (Do not worry if you cannot see all your text once you press return)

1.a. How was the sample size chosen to ensure adequate power to detect a pre-specified effect size?	No statistical test was used to determine sample size
1.b. For animal studies, include a statement about sample size estimate even if no statistical methods were used.	Doesn't apply
2. Describe inclusion/exclusion criteria if samples or animals were excluded from the analysis. Were the criteria pre-established?	No samples were excluded
3. Were any steps taken to minimize the effects of subjective bias when allocating animals/samples to treatment (e.g. randomization procedure)? If yes, please describe.	No randomization was performed
For animal studies, include a statement about randomization even if no randomization was used.	Doesn't apply
4.a. Were any steps taken to minimize the effects of subjective bias during group allocation or/and when assessing results (e.g. blinding of the investigator)? If yes please describe.	No blinding was performed
4.b. For animal studies, include a statement about blinding even if no blinding was done	Doesn't apply
5. For every figure, are statistical tests justified as appropriate?	Yes
Do the data meet the assumptions of the tests (e.g., normal distribution)? Describe any methods used to assess it.	Mann-Whitney test was used (Fig. 6B), no normal ditribution of the data is required.
Is there an estimate of variation within each group of data?	Variance was not estimated

Is the variance similar between the groups that are being statistically compared?	Doesn't apply

C- Reagents

6. To show that antibodies were profiled for use in the system under study (assay and species), provide a citation, catalog number and/or clone number, supplementary information or reference to an antibody validation profile. e.g., Antibodypedia (see link list at top right), 1DegreeBio (see link list at top right).	No antibodies were used
7. Identify the source of cell lines and report if they were recently authenticated (e.g., by STR profiling) and tested for mycoplasma contamination.	No cell lines were used

* for all hyperlinks, please see the table at the top right of the document

D- Animal Models

8. Report species, strain, gender, age of animals and genetic modification status where applicable. Please detail housing and husbandry conditions and the source of animals.	Doesn't apply
9. For experiments involving live vertebrates, include a statement of compliance with ethical regulations and identify the committee(s) approving the experiments.	Doesn't apply
10. We recommend consulting the ARRIVE guidelines (see link list at top right) (PLoS Biol. 8(6), e1000412, 2010) to ensure that other relevant aspects of animal studies are adequately reported. See author guidelines, under 'Reporting Guidelines'. See also: NIH (see link list at top right) and MRC (see link list at top right) recommendations. Please confirm compliance.	Doesn't apply

E- Human Subjects

11. Identify the committee(s) approving the study protocol.	Doesn't apply
12. Include a statement confirming that informed consent was obtained from all subjects and that the experiments conformed to the principles set out in the WMA Declaration of Helsinki and the Department of Health and Human Services Belmont Report.	Doesn't apply
13. For publication of patient photos, include a statement confirming that consent to publish was obtained.	Doesn't apply
14. Report any restrictions on the availability (and/or on the use) of human data or samples.	Doesn't apply
15. Report the clinical trial registration number (at ClinicalTrials.gov or equivalent), where applicable.	Doesn't apply
16. For phase II and III randomized controlled trials, please refer to the CONSORT flow diagram (see link list at top right) and submit the CONSORT checklist (see link list at top right) with your submission. See author guidelines, under 'Reporting Guidelines'. Please confirm you have submitted this list.	Doesn't apply
17. For tumor marker prognostic studies, we recommend that you follow the REMARK reporting guidelines (see link list at top right). See author guidelines, under 'Reporting Guidelines'. Please confirm you have followed these guidelines.	Doesn't apply

F- Data Accessibility

18: Provide a "Data Availability" section at the end of the Materials & Methods, listing the accession codes for data generated in this study and deposited in a public database (e.g. RNA-Seq data: Gene Expression Omnibus GSE39462, Proteomics data: PRIDE PXD000208 etc.) Please refer to our author guidelines for 'Data Deposition'.<br><br>Data deposition in a public repository is mandatory for:<br>a. Protein, DNA and RNA sequences<br>b. Macromolecular structures<br>c. Crystallographic data for small molecules<br>d. Functional genomics data<br>e. Proteomics and molecular interactions	Riboseq dataset ID is provided
19. Deposition is strongly recommended for any datasets that are central and integral to the study; please consider the journal's data policy. If no structured public repository exists for a given data type, we encourage the provision of datasets in the manuscript as a Supplementary Document (see author guidelines under 'Expanded View' or in unstructured repositories such as Dryad (see link list at top right) or Figshare (see link list at top right).	Doesn't apply
20. Access to human clinical and genomic datasets should be provided with as few restrictions as possible while respecting ethical obligations to the patients and relevant medical and legal issues. If practically possible and compatible with the individual consent agreement used in the study, such data should be deposited in one of the major public access-controlled repositories such as dbGAP (see link list at top right) or EGA (see link list at top right).	Doesn't apply
21. Computational models that are central and integral to a study should be shared without restrictions and provided in a machine-readable form. The relevant accession numbers or links should be provided. When possible, standardized format (SBML, CellML) should be used instead of scripts (e.g. MATLAB). Authors are strongly encouraged to follow the MIRIAM guidelines (see link list at top right) and deposit their model in a public database such as Biomodels (see link list at top right) or JWS Online (see link list at top right). If computer source code is provided with the paper, it should be deposited in a public repository or included in supplementary information.	Doesn't apply

G- Dual use research of concern

22. Could your study fall under dual use research restrictions? Please check biosecurity documents (see link list at top right) and list of select agents and toxins (APHIS/CDC) (see link list at top right). According to our biosecurity guidelines, provide a statement only if it could.	The study doesn't fall under dual use research restrictions.
